# Search for heavy neutrinos and $\mathrm {W}$ bosons with right-handed couplings in proton–proton collisions at $\sqrt{s} = 8\,\text {TeV}$

**DOI:** 10.1140/epjc/s10052-014-3149-z

**Published:** 2014-11-26

**Authors:** CMS Collaboration, V. Khachatryan, A. M. Sirunyan, A. Tumasyan, W. Adam, T. Bergauer, M. Dragicevic, J. Erö, C. Fabjan, M. Friedl, R. Frühwirth, V. M. Ghete, C. Hartl, N. Hörmann, J. Hrubec, M. Jeitler, W. Kiesenhofer, V. Knünz, M. Krammer, I. Krätschmer, D. Liko, I. Mikulec, D. Rabady, B. Rahbaran, H. Rohringer, R. Schöfbeck, J. Strauss, A. Taurok, W. Treberer-Treberspurg, W. Waltenberger, C.-E. Wulz, V. Mossolov, N. Shumeiko, J. Suarez Gonzalez, S. Alderweireldt, M. Bansal, S. Bansal, T. Cornelis, E. A. De Wolf, X. Janssen, A. Knutsson, S. Luyckx, S. Ochesanu, B. Roland, R. Rougny, M. Van De Klundert, H. Van Haevermaet, P. Van Mechelen, N. Van Remortel, A. Van Spilbeeck, F. Blekman, S. Blyweert, J. D’Hondt, N. Daci, N. Heracleous, J. Keaveney, S. Lowette, M. Maes, A. Olbrechts, Q. Python, D. Strom, S. Tavernier, W. Van Doninck, P. Van Mulders, G. P. Van Onsem, I. Villella, C. Caillol, B. Clerbaux, G. De Lentdecker, D. Dobur, L. Favart, A. P. R. Gay, A. Grebenyuk, A. Léonard, A. Mohammadi, L. Perniè, T. Reis, T. Seva, L. Thomas, C. Vander Velde, P. Vanlaer, J. Wang, V. Adler, K. Beernaert, L. Benucci, A. Cimmino, S. Costantini, S. Crucy, S. Dildick, A. Fagot, G. Garcia, J. Mccartin, A. A. Ocampo Rios, D. Ryckbosch, S. Salva Diblen, M. Sigamani, N. Strobbe, F. Thyssen, M. Tytgat, E. Yazgan, N. Zaganidis, S. Basegmez, C. Beluffi, G. Bruno, R. Castello, A. Caudron, L. Ceard, G. G. Da Silveira, C. Delaere, T. du Pree, D. Favart, L. Forthomme, A. Giammanco, J. Hollar, P. Jez, M. Komm, V. Lemaitre, C. Nuttens, D. Pagano, L. Perrini, A. Pin, K. Piotrzkowski, A. Popov, L. Quertenmont, M. Selvaggi, M. Vidal Marono, J. M. Vizan Garcia, N. Beliy, T. Caebergs, E. Daubie, G. H. Hammad, W. L. Aldá Júnior, G. A. Alves, L. Brito, M. Correa Martins Junior, M. E. Pol, W. Carvalho, J. Chinellato, A. Custódio, E. M. Da Costa, D. De Jesus Damiao, C. De Oliveira Martins, S. Fonseca De Souza, H. Malbouisson, D. Matos Figueiredo, L. Mundim, H. Nogima, W. L. Prado Da Silva, J. Santaolalla, A. Santoro, A. Sznajder, E. J. Tonelli Manganote, A. Vilela Pereira, C. A. Bernardes, T. R. Fernandez Perez Tomei, E. M. Gregores, P. G. Mercadante, S. F. Novaes, Sandra S. Padula, A. Aleksandrov, V. Genchev, P. Iaydjiev, A. Marinov, S. Piperov, M. Rodozov, G. Sultanov, M. Vutova, A. Dimitrov, I. Glushkov, R. Hadjiiska, V. Kozhuharov, L. Litov, B. Pavlov, P. Petkov, J. G. Bian, G. M. Chen, H. S. Chen, M. Chen, R. Du, C. H. Jiang, D. Liang, S. Liang, R. Plestina, J. Tao, X. Wang, Z. Wang, C. Asawatangtrakuldee, Y. Ban, Y. Guo, Q. Li, W. Li, S. Liu, Y. Mao, S. J. Qian, D. Wang, L. Zhang, W. Zou, C. Avila, L. F. Chaparro Sierra, C. Florez, J. P. Gomez, B. Gomez Moreno, J. C. Sanabria, N. Godinovic, D. Lelas, D. Polic, I. Puljak, Z. Antunovic, M. Kovac, V. Brigljevic, K. Kadija, J. Luetic, D. Mekterovic, L. Sudic, A. Attikis, G. Mavromanolakis, J. Mousa, C. Nicolaou, F. Ptochos, P. A. Razis, M. Bodlak, M. Finger, M. Finger, Y. Assran, S. Elgammal, M. A. Mahmoud, A. Radi, M. Kadastik, M. Murumaa, M. Raidal, A. Tiko, P. Eerola, G. Fedi, M. Voutilainen, J. Härkönen, V. Karimäki, R. Kinnunen, M. J. Kortelainen, T. Lampén, K. Lassila-Perini, S. Lehti, T. Lindén, P. Luukka, T. Mäenpää, T. Peltola, E. Tuominen, J. Tuominiemi, E. Tuovinen, L. Wendland, T. Tuuva, M. Besancon, F. Couderc, M. Dejardin, D. Denegri, B. Fabbro, J. L. Faure, C. Favaro, F. Ferri, S. Ganjour, A. Givernaud, P. Gras, G. Hamel de Monchenault, P. Jarry, E. Locci, J. Malcles, J. Rander, A. Rosowsky, M. Titov, S. Baffioni, F. Beaudette, P. Busson, C. Charlot, T. Dahms, M. Dalchenko, L. Dobrzynski, N. Filipovic, A. Florent, R. Granier de Cassagnac, L. Mastrolorenzo, P. Miné, C. Mironov, I. N. Naranjo, M. Nguyen, C. Ochando, P. Paganini, R. Salerno, J. b. Sauvan, Y. Sirois, C. Veelken, Y. Yilmaz, A. Zabi, J.-L. Agram, J. Andrea, A. Aubin, D. Bloch, J.-M. Brom, E. C. Chabert, C. Collard, E. Conte, J.-C. Fontaine, D. Gelé, U. Goerlach, C. Goetzmann, A.-C. Le Bihan, P. Van Hove, S. Gadrat, S. Beauceron, N. Beaupere, G. Boudoul, S. Brochet, C. A. Carrillo Montoya, J. Chasserat, R. Chierici, D. Contardo, P. Depasse, H. El Mamouni, J. Fan, J. Fay, S. Gascon, M. Gouzevitch, B. Ille, T. Kurca, M. Lethuillier, L. Mirabito, S. Perries, J. D. Ruiz Alvarez, D. Sabes, L. Sgandurra, V. Sordini, M. Vander Donckt, P. Verdier, S. Viret, H. Xiao, I. Bagaturia, C. Autermann, S. Beranek, M. Bontenackels, M. Edelhoff, L. Feld, O. Hindrichs, K. Klein, A. Ostapchuk, A. Perieanu, F. Raupach, J. Sammet, S. Schael, H. Weber, B. Wittmer, V. Zhukov, M. Ata, E. Dietz-Laursonn, D. Duchardt, M. Erdmann, R. Fischer, A. Güth, T. Hebbeker, C. Heidemann, K. Hoepfner, D. Klingebiel, S. Knutzen, P. Kreuzer, M. Merschmeyer, A. Meyer, P. Millet, M. Olschewski, K. Padeken, P. Papacz, H. Reithler, S. A. Schmitz, L. Sonnenschein, D. Teyssier, S. Thüer, M. Weber, V. Cherepanov, Y. Erdogan, G. Flügge, H. Geenen, M. Geisler, W. Haj Ahmad, F. Hoehle, B. Kargoll, T. Kress, Y. Kuessel, J. Lingemann, A. Nowack, I. M. Nugent, L. Perchalla, O. Pooth, A. Stahl, I. Asin, N. Bartosik, J. Behr, W. Behrenhoff, U. Behrens, A. J. Bell, M. Bergholz, A. Bethani, K. Borras, A. Burgmeier, A. Cakir, L. Calligaris, A. Campbell, S. Choudhury, F. Costanza, C. Diez Pardos, S. Dooling, T. Dorland, G. Eckerlin, D. Eckstein, T. Eichhorn, G. Flucke, J. Garay Garcia, A. Geiser, P. Gunnellini, J. Hauk, G. Hellwig, M. Hempel, D. Horton, H. Jung, A. Kalogeropoulos, M. Kasemann, P. Katsas, J. Kieseler, C. Kleinwort, D. Krücker, W. Lange, J. Leonard, K. Lipka, A. Lobanov, W. Lohmann, B. Lutz, R. Mankel, I. Marfin, I.-A. Melzer-Pellmann, A. B. Meyer, J. Mnich, A. Mussgiller, S. Naumann-Emme, A. Nayak, O. Novgorodova, F. Nowak, E. Ntomari, H. Perrey, D. Pitzl, R. Placakyte, A. Raspereza, P. M. Ribeiro Cipriano, E. Ron, M. Ö. Sahin, J. Salfeld-Nebgen, P. Saxena, R. Schmidt, T. Schoerner-Sadenius, M. Schröder, C. Seitz, S. Spannagel, A. D. R. Vargas Trevino, R. Walsh, C. Wissing, M. Aldaya Martin, V. Blobel, M. Centis Vignali, A. r. Draeger, J. Erfle, E. Garutti, K. Goebel, M. Görner, J. Haller, M. Hoffmann, R. S. Höing, H. Kirschenmann, R. Klanner, R. Kogler, J. Lange, T. Lapsien, T. Lenz, I. Marchesini, J. Ott, T. Peiffer, N. Pietsch, T. Pöhlsen, D. Rathjens, C. Sander, H. Schettler, P. Schleper, E. Schlieckau, A. Schmidt, M. Seidel, J. Sibille, V. Sola, H. Stadie, G. Steinbrück, D. Troendle, E. Usai, L. Vanelderen, C. Barth, C. Baus, J. Berger, C. Böser, E. Butz, T. Chwalek, W. De Boer, A. Descroix, A. Dierlamm, M. Feindt, F. Frensch, M. Giffels, F. Hartmann, T. Hauth, U. Husemann, I. Katkov, A. Kornmayer, E. Kuznetsova, P. Lobelle Pardo, M. U. Mozer, Th. Müller, A. Nürnberg, G. Quast, K. Rabbertz, F. Ratnikov, S. Röcker, H. J. Simonis, F. M. Stober, R. Ulrich, J. Wagner-Kuhr, S. Wayand, T. Weiler, R. Wolf, G. Anagnostou, G. Daskalakis, T. Geralis, V. A. Giakoumopoulou, A. Kyriakis, D. Loukas, A. Markou, C. Markou, A. Psallidas, I. Topsis-Giotis, A. Panagiotou, N. Saoulidou, E. Stiliaris, X. Aslanoglou, I. Evangelou, G. Flouris, C. Foudas, P. Kokkas, N. Manthos, I. Papadopoulos, E. Paradas, G. Bencze, C. Hajdu, P. Hidas, D. Horvath, F. Sikler, V. Veszpremi, G. Vesztergombi, A. J. Zsigmond, N. Beni, S. Czellar, J. Karancsi, J. Molnar, J. Palinkas, Z. Szillasi, P. Raics, Z. L. Trocsanyi, B. Ujvari, S. K. Swain, S. B. Beri, V. Bhatnagar, N. Dhingra, R. Gupta, U. Bhawandeep, A. K. Kalsi, M. Kaur, M. Mittal, N. Nishu, J. B. Singh, Ashok Kumar, Arun Kumar, S. Ahuja, A. Bhardwaj, B. C. Choudhary, A. Kumar, S. Malhotra, M. Naimuddin, K. Ranjan, V. Sharma, S. Banerjee, S. Bhattacharya, K. Chatterjee, S. Dutta, B. Gomber, Sa. Jain, Sh. Jain, R. Khurana, A. Modak, S. Mukherjee, D. Roy, S. Sarkar, M. Sharan, A. Abdulsalam, D. Dutta, S. Kailas, V. Kumar, A. K. Mohanty, L. M. Pant, P. Shukla, A. Topkar, T. Aziz, S. Banerjee, S. Bhowmik, R. M. Chatterjee, R. K. Dewanjee, S. Dugad, S. Ganguly, S. Ghosh, M. Guchait, A. Gurtu, G. Kole, S. Kumar, M. Maity, G. Majumder, K. Mazumdar, G. B. Mohanty, B. Parida, K. Sudhakar, N. Wickramage, H. Bakhshiansohi, H. Behnamian, S. M. Etesami, A. Fahim, R. Goldouzian, A. Jafari, M. Khakzad, M. Mohammadi Najafabadi, M. Naseri, S. Paktinat Mehdiabadi, B. Safarzadeh, M. Zeinali, M. Felcini, M. Grunewald, M. Abbrescia, L. Barbone, C. Calabria, S. S. Chhibra, A. Colaleo, D. Creanza, N. De Filippis, M. De Palma, L. Fiore, G. Iaselli, G. Maggi, M. Maggi, S. My, S. Nuzzo, A. Pompili, G. Pugliese, R. Radogna, G. Selvaggi, L. Silvestris, G. Singh, R. Venditti, P. Verwilligen, G. Zito, G. Abbiendi, A. C. Benvenuti, D. Bonacorsi, S. Braibant-Giacomelli, L. Brigliadori, R. Campanini, P. Capiluppi, A. Castro, F. R. Cavallo, G. Codispoti, M. Cuffiani, G. M. Dallavalle, F. Fabbri, A. Fanfani, D. Fasanella, P. Giacomelli, C. Grandi, L. Guiducci, S. Marcellini, G. Masetti, A. Montanari, F. L. Navarria, A. Perrotta, F. Primavera, A. M. Rossi, T. Rovelli, G. P. Siroli, N. Tosi, R. Travaglini, S. Albergo, G. Cappello, M. Chiorboli, S. Costa, F. Giordano, R. Potenza, A. Tricomi, C. Tuve, G. Barbagli, V. Ciulli, C. Civinini, R. D’Alessandro, E. Focardi, E. Gallo, S. Gonzi, V. Gori, P. Lenzi, M. Meschini, S. Paoletti, G. Sguazzoni, A. Tropiano, L. Benussi, S. Bianco, F. Fabbri, D. Piccolo, F. Ferro, M. Lo Vetere, E. Robutti, S. Tosi, M. E. Dinardo, P. Dini, S. Fiorendi, S. Gennai, R. Gerosa, A. Ghezzi, P. Govoni, M. T. Lucchini, S. Malvezzi, R. A. Manzoni, A. Martelli, B. Marzocchi, D. Menasce, L. Moroni, M. Paganoni, S. Ragazzi, N. Redaelli, T. Tabarelli de Fatis, S. Buontempo, N. Cavallo, S. Di Guida, F. Fabozzi, A. O. M. Iorio, L. Lista, S. Meola, M. Merola, P. Paolucci, P. Azzi, N. Bacchetta, D. Bisello, A. Branca, R. Carlin, P. Checchia, M. Dall’Osso, T. Dorigo, M. Galanti, F. Gasparini, U. Gasparini, P. Giubilato, F. Gonella, A. Gozzelino, K. Kanishchev, S. Lacaprara, M. Margoni, A. T. Meneguzzo, F. Montecassiano, J. Pazzini, N. Pozzobon, P. Ronchese, F. Simonetto, E. Torassa, M. Tosi, P. Zotto, A. Zucchetta, M. Gabusi, S. P. Ratti, C. Riccardi, P. Salvini, P. Vitulo, M. Biasini, G. M. Bilei, D. Ciangottini, L. Fanò, P. Lariccia, G. Mantovani, M. Menichelli, F. Romeo, A. Saha, A. Santocchia, A. Spiezia, K. Androsov, P. Azzurri, G. Bagliesi, J. Bernardini, T. Boccali, G. Broccolo, R. Castaldi, M. A. Ciocci, R. Dell’Orso, S. Donato, F. Fiori, L. Foà, A. Giassi, M. T. Grippo, F. Ligabue, T. Lomtadze, L. Martini, A. Messineo, C. S. Moon, F. Palla, A. Rizzi, A. Savoy-Navarro, A. T. Serban, P. Spagnolo, P. Squillacioti, R. Tenchini, G. Tonelli, A. Venturi, P. G. Verdini, C. Vernieri, L. Barone, F. Cavallari, D. Del Re, M. Diemoz, M. Grassi, C. Jorda, E. Longo, F. Margaroli, P. Meridiani, F. Micheli, S. Nourbakhsh, G. Organtini, R. Paramatti, S. Rahatlou, C. Rovelli, F. Santanastasio, L. Soffi, P. Traczyk, N. Amapane, R. Arcidiacono, S. Argiro, M. Arneodo, R. Bellan, C. Biino, N. Cartiglia, S. Casasso, M. Costa, A. Degano, N. Demaria, L. Finco, C. Mariotti, S. Maselli, E. Migliore, V. Monaco, M. Musich, M. M. Obertino, G. Ortona, L. Pacher, N. Pastrone, M. Pelliccioni, G. L. Pinna Angioni, A. Potenza, A. Romero, M. Ruspa, R. Sacchi, A. Solano, A. Staiano, U. Tamponi, S. Belforte, V. Candelise, M. Casarsa, F. Cossutti, G. Della Ricca, B. Gobbo, C. La Licata, M. Marone, D. Montanino, A. Schizzi, T. Umer, A. Zanetti, T. J. Kim, S. Chang, A. Kropivnitskaya, S. K. Nam, D. H. Kim, G. N. Kim, M. S. Kim, M. S. Kim, D. J. Kong, S. Lee, Y. D. Oh, H. Park, A. Sakharov, D. C. Son, J. Y. Kim, S. Song, S. Choi, D. Gyun, B. Hong, M. Jo, H. Kim, Y. Kim, B. Lee, K. S. Lee, S. K. Park, Y. Roh, M. Choi, J. H. Kim, I. C. Park, S. Park, G. Ryu, M. S. Ryu, Y. Choi, Y. K. Choi, J. Goh, D. Kim, E. Kwon, J. Lee, H. Seo, I. Yu, A. Juodagalvis, J. R. Komaragiri, M.A.B. Md Ali, H. Castilla-Valdez, E. De La Cruz-Burelo, I. Heredia-de La Cruz, R. Lopez-Fernandez, A. Sanchez-Hernandez, S. Carrillo Moreno, F. Vazquez Valencia, I. Pedraza, H. A. Salazar Ibarguen, E. Casimiro Linares, A. Morelos Pineda, D. Krofcheck, P. H. Butler, S. Reucroft, A. Ahmad, M. Ahmad, Q. Hassan, H. R. Hoorani, S. Khalid, W. A. Khan, T. Khurshid, M. A. Shah, M. Shoaib, H. Bialkowska, M. Bluj, B. Boimska, T. Frueboes, M. Górski, M. Kazana, K. Nawrocki, K. Romanowska-Rybinska, M. Szleper, P. Zalewski, G. Brona, K. Bunkowski, M. Cwiok, W. Dominik, K. Doroba, A. Kalinowski, M. Konecki, J. Krolikowski, M. Misiura, M. Olszewski, W. Wolszczak, P. Bargassa, C. Beirão Da Cruz E Silva, P. Faccioli, P. G. Ferreira Parracho, M. Gallinaro, F. Nguyen, J. Rodrigues Antunes, J. Seixas, J. Varela, P. Vischia, P. Bunin, M. Gavrilenko, I. Golutvin, A. Kamenev, V. Karjavin, V. Konoplyanikov, A. Lanev, A. Malakhov, V. Matveev, P. Moisenz, V. Palichik, V. Perelygin, M. Savina, S. Shmatov, S. Shulha, N. Skatchkov, V. Smirnov, A. Zarubin, V. Golovtsov, Y. Ivanov, V. Kim, P. Levchenko, V. Murzin, V. Oreshkin, I. Smirnov, V. Sulimov, L. Uvarov, S. Vavilov, A. Vorobyev, An. Vorobyev, Yu. Andreev, A. Dermenev, S. Gninenko, N. Golubev, M. Kirsanov, N. Krasnikov, A. Pashenkov, D. Tlisov, A. Toropin, V. Epshteyn, V. Gavrilov, N. Lychkovskaya, V. Popov, G. Safronov, S. Semenov, A. Spiridonov, V. Stolin, E. Vlasov, A. Zhokin, V. Andreev, M. Azarkin, I. Dremin, M. Kirakosyan, A. Leonidov, G. Mesyats, S. V. Rusakov, A. Vinogradov, A. Belyaev, E. Boos, V. Bunichev, M. Dubinin, L. Dudko, A. Ershov, A. Gribushin, V. Klyukhin, O. Kodolova, I. Lokhtin, S. Obraztsov, S. Petrushanko, V. Savrin, I. Azhgirey, I. Bayshev, S. Bitioukov, V. Kachanov, A. Kalinin, D. Konstantinov, V. Krychkine, V. Petrov, R. Ryutin, A. Sobol, L. Tourtchanovitch, S. Troshin, N. Tyurin, A. Uzunian, A. Volkov, P. Adzic, M. Ekmedzic, J. Milosevic, V. Rekovic, J. Alcaraz Maestre, C. Battilana, E. Calvo, M. Cerrada, M. Chamizo Llatas, N. Colino, B. De La Cruz, A. Delgado Peris, D. Domínguez Vázquez, A. Escalante Del Valle, C. Fernandez Bedoya, J. P. Fernández Ramos, J. Flix, M. C. Fouz, P. Garcia-Abia, O. Gonzalez Lopez, S. Goy Lopez, J. M. Hernandez, M. I. Josa, G. Merino, E. Navarro De Martino, A. Pérez-Calero Yzquierdo, J. Puerta Pelayo, A. Quintario Olmeda, I. Redondo, L. Romero, M. S. Soares, C. Albajar, J. F. de Trocóniz, M. Missiroli, D. Moran, H. Brun, J. Cuevas, J. Fernandez Menendez, S. Folgueras, I. Gonzalez Caballero, L. Lloret Iglesias, J. A. Brochero Cifuentes, I. J. Cabrillo, A. Calderon, J. Duarte Campderros, M. Fernandez, G. Gomez, A. Graziano, A. Lopez Virto, J. Marco, R. Marco, C. Martinez Rivero, F. Matorras, F. J. Munoz Sanchez, J. Piedra Gomez, T. Rodrigo, A. Y. Rodríguez-Marrero, A. Ruiz-Jimeno, L. Scodellaro, I. Vila, R. Vilar Cortabitarte, D. Abbaneo, E. Auffray, G. Auzinger, M. Bachtis, P. Baillon, A. H. Ball, D. Barney, A. Benaglia, J. Bendavid, L. Benhabib, J. F. Benitez, C. Bernet, G. Bianchi, P. Bloch, A. Bocci, A. Bonato, O. Bondu, C. Botta, H. Breuker, T. Camporesi, G. Cerminara, S. Colafranceschi, M. D’Alfonso, D. d’Enterria, A. Dabrowski, A. David, F. De Guio, A. De Roeck, S. De Visscher, M. Dobson, M. Dordevic, N. Dupont-Sagorin, A. Elliott-Peisert, J. Eugster, G. Franzoni, W. Funk, D. Gigi, K. Gill, D. Giordano, M. Girone, F. Glege, R. Guida, S. Gundacker, M. Guthoff, R. Guida, J. Hammer, M. Hansen, P. Harris, J. Hegeman, V. Innocente, P. Janot, K. Kousouris, K. Krajczar, P. Lecoq, C. Lourenço, N. Magini, L. Malgeri, M. Mannelli, J. Marrouche, L. Masetti, F. Meijers, S. Mersi, E. Meschi, F. Moortgat, S. Morovic, M. Mulders, P. Musella, L. Orsini, L. Pape, E. Perez, L. Perrozzi, A. Petrilli, G. Petrucciani, A. Pfeiffer, M. Pierini, M. Pimiä, D. Piparo, M. Plagge, A. Racz, G. Rolandi, M. Rovere, H. Sakulin, C. Schäfer, C. Schwick, A. Sharma, P. Siegrist, P. Silva, M. Simon, P. Sphicas, D. Spiga, J. Steggemann, B. Stieger, M. Stoye, D. Treille, A. Tsirou, G. I. Veres, J. R. Vlimant, N. Wardle, H. K. Wöhri, H. Wollny, W. D. Zeuner, W. Bertl, K. Deiters, W. Erdmann, R. Horisberger, Q. Ingram, H. C. Kaestli, S. König, D. Kotlinski, U. Langenegger, D. Renker, T. Rohe, F. Bachmair, L. Bäni, L. Bianchini, P. Bortignon, M. A. Buchmann, B. Casal, N. Chanon, A. Deisher, G. Dissertori, M. Dittmar, M. Donegà, M. Dünser, P. Eller, C. Grab, D. Hits, W. Lustermann, B. Mangano, A. C. Marini, P. Martinez Ruiz del Arbol, D. Meister, N. Mohr, C. Nägeli, F. Nessi-Tedaldi, F. Pandolfi, F. Pauss, M. Peruzzi, M. Quittnat, L. Rebane, M. Rossini, A. Starodumov, M. Takahashi, K. Theofilatos, R. Wallny, H. A. Weber, C. Amsler, M. F. Canelli, V. Chiochia, A. De Cosa, A. Hinzmann, T. Hreus, B. Kilminster, B. Millan Mejias, J. Ngadiuba, P. Robmann, F. J. Ronga, S. Taroni, M. Verzetti, Y. Yang, M. Cardaci, K. H. Chen, C. Ferro, C. M. Kuo, W. Lin, Y. J. Lu, R. Volpe, S. S. Yu, P. Chang, Y. H. Chang, Y. W. Chang, Y. Chao, K. F. Chen, P. H. Chen, C. Dietz, U. Grundler, W.-S. Hou, K. Y. Kao, Y. J. Lei, Y. F. Liu, R.-S. Lu, D. Majumder, E. Petrakou, Y. M. Tzeng, R. Wilken, B. Asavapibhop, N. Srimanobhas, N. Suwonjandee, A. Adiguzel, M. N. Bakirci, S. Cerci, C. Dozen, I. Dumanoglu, E. Eskut, S. Girgis, G. Gokbulut, E. Gurpinar, I. Hos, E. E. Kangal, A. Kayis Topaksu, G. Onengut, K. Ozdemir, S. Ozturk, A. Polatoz, K. Sogut, D. Sunar Cerci, B. Tali, H. Topakli, M. Vergili, I. V. Akin, B. Bilin, S. Bilmis, H. Gamsizkan, G. Karapinar, K. Ocalan, S. Sekmen, U. E. Surat, M. Yalvac, M. Zeyrek, E. Gülmez, B. Isildak, M. Kaya, O. Kaya, H. Bahtiyar, E. Barlas, K. Cankocak, F. I. Vardarlı, M. Yücel, L. Levchuk, P. Sorokin, J. J. Brooke, E. Clement, D. Cussans, H. Flacher, R. Frazier, J. Goldstein, M. Grimes, G. P. Heath, H. F. Heath, J. Jacob, L. Kreczko, C. Lucas, Z. Meng, D. M. Newbold, S. Paramesvaran, A. Poll, S. Senkin, V. J. Smith, T. Williams, K. W. Bell, A. Belyaev, C. Brew, R. M. Brown, D. J. A. Cockerill, J. A. Coughlan, K. Harder, S. Harper, E. Olaiya, D. Petyt, C. H. Shepherd-Themistocleous, A. Thea, I. R. Tomalin, W. J. Womersley, S. D. Worm, M. Baber, R. Bainbridge, O. Buchmuller, D. Burton, D. Colling, N. Cripps, M. Cutajar, P. Dauncey, G. Davies, M. Della Negra, P. Dunne, W. Ferguson, J. Fulcher, D. Futyan, A. Gilbert, G. Hall, G. Iles, M. Jarvis, G. Karapostoli, M. Kenzie, R. Lane, R. Lucas, L. Lyons, A.-M. Magnan, S. Malik, B. Mathias, J. Nash, A. Nikitenko, J. Pela, M. Pesaresi, K. Petridis, D. M. Raymond, S. Rogerson, A. Rose, C. Seez, P. Sharp, A. Tapper, M. Vazquez Acosta, T. Virdee, J. E. Cole, P. R. Hobson, A. Khan, P. Kyberd, D. Leggat, D. Leslie, W. Martin, I. D. Reid, P. Symonds, L. Teodorescu, M. Turner, J. Dittmann, K. Hatakeyama, A. Kasmi, H. Liu, T. Scarborough, O. Charaf, S. I. Cooper, C. Henderson, P. Rumerio, A. Avetisyan, T. Bose, C. Fantasia, A. Heister, P. Lawson, C. Richardson, J. Rohlf, D. Sperka, J. St. John, L. Sulak, J. Alimena, E. Berry, S. Bhattacharya, G. Christopher, D. Cutts, Z. Demiragli, A. Ferapontov, A. Garabedian, U. Heintz, G. Kukartsev, E. Laird, G. Landsberg, M. Luk, M. Narain, M. Segala, T. Sinthuprasith, T. Speer, J. Swanson, R. Breedon, G. Breto, M. Calderon De La Barca Sanchez, S. Chauhan, M. Chertok, J. Conway, R. Conway, P. T. Cox, R. Erbacher, M. Gardner, W. Ko, R. Lander, T. Miceli, M. Mulhearn, D. Pellett, J. Pilot, F. Ricci-Tam, M. Searle, S. Shalhout, J. Smith, M. Squires, D. Stolp, M. Tripathi, S. Wilbur, R. Yohay, R. Cousins, P. Everaerts, C. Farrell, J. Hauser, M. Ignatenko, G. Rakness, E. Takasugi, V. Valuev, M. Weber, J. Babb, K. Burt, R. Clare, J. Ellison, J. W. Gary, G. Hanson, J. Heilman, M. Ivova Rikova, P. Jandir, E. Kennedy, F. Lacroix, H. Liu, O. R. Long, A. Luthra, M. Malberti, H. Nguyen, M. Olmedo Negrete, A. Shrinivas, S. Sumowidagdo, S. Wimpenny, W. Andrews, J. G. Branson, G. B. Cerati, S. Cittolin, R. T. D’Agnolo, D. Evans, A. Holzner, R. Kelley, D. Klein, M. Lebourgeois, J. Letts, I. Macneill, D. Olivito, S. Padhi, C. Palmer, M. Pieri, M. Sani, V. Sharma, S. Simon, E. Sudano, M. Tadel, Y. Tu, A. Vartak, C. Welke, F. Würthwein, A. Yagil, J. Yoo, D. Barge, J. Bradmiller-Feld, C. Campagnari, T. Danielson, A. Dishaw, K. Flowers, M. Franco Sevilla, P. Geffert, C. George, F. Golf, L. Gouskos, J. Incandela, C. Justus, N. Mccoll, J. Richman, D. Stuart, W. To, C. West, A. Apresyan, A. Bornheim, J. Bunn, Y. Chen, E. Di Marco, J. Duarte, A. Mott, H. B. Newman, C. Pena, C. Rogan, M. Spiropulu, V. Timciuc, R. Wilkinson, S. Xie, R. Y. Zhu, V. Azzolini, A. Calamba, T. Ferguson, Y. Iiyama, M. Paulini, J. Russ, H. Vogel, I. Vorobiev, J. P. Cumalat, W. T. Ford, A. Gaz, E. Luiggi Lopez, U. Nauenberg, J. G. Smith, K. Stenson, K. A. Ulmer, S. R. Wagner, J. Alexander, A. Chatterjee, J. Chu, S. Dittmer, N. Eggert, N. Mirman, G. Nicolas Kaufman, J. R. Patterson, A. Ryd, E. Salvati, L. Skinnari, W. Sun, W. D. Teo, J. Thom, J. Thompson, J. Tucker, Y. Weng, L. Winstrom, P. Wittich, D. Winn, S. Abdullin, M. Albrow, J. Anderson, G. Apollinari, L. A. T. Bauerdick, A. Beretvas, J. Berryhill, P. C. Bhat, K. Burkett, J. N. Butler, H. W. K. Cheung, F. Chlebana, S. Cihangir, V. D. Elvira, I. Fisk, J. Freeman, Y. Gao, E. Gottschalk, L. Gray, D. Green, S. Grünendahl, O. Gutsche, J. Hanlon, D. Hare, R. M. Harris, J. Hirschauer, B. Hooberman, S. Jindariani, M. Johnson, U. Joshi, K. Kaadze, B. Klima, B. Kreis, S. Kwan, J. Linacre, D. Lincoln, R. Lipton, T. Liu, J. Lykken, K. Maeshima, J. M. Marraffino, V. I. Martinez Outschoorn, S. Maruyama, D. Mason, P. McBride, K. Mishra, S. Mrenna, Y. Musienko, S. Nahn, C. Newman-Holmes, V. O’Dell, O. Prokofyev, E. Sexton-Kennedy, S. Sharma, A. Soha, W. J. Spalding, L. Spiegel, L. Taylor, S. Tkaczyk, N. V. Tran, L. Uplegger, E. W. Vaandering, R. Vidal, A. Whitbeck, J. Whitmore, F. Yang, D. Acosta, P. Avery, D. Bourilkov, M. Carver, T. Cheng, D. Curry, S. Das, M. De Gruttola, G. P. Di Giovanni, R. D. Field, M. Fisher, I. K. Furic, J. Hugon, J. Konigsberg, A. Korytov, T. Kypreos, J. F. Low, K. Matchev, P. Milenovic, G. Mitselmakher, L. Muniz, A. Rinkevicius, L. Shchutska, N. Skhirtladze, M. Snowball, J. Yelton, M. Zakaria, S. Hewamanage, S. Linn, P. Markowitz, G. Martinez, J. L. Rodriguez, T. Adams, A. Askew, J. Bochenek, B. Diamond, J. Haas, S. Hagopian, V. Hagopian, K. F. Johnson, H. Prosper, V. Veeraraghavan, M. Weinberg, M. M. Baarmand, M. Hohlmann, H. Kalakhety, F. Yumiceva, M. R. Adams, L. Apanasevich, V. E. Bazterra, D. Berry, R. R. Betts, I. Bucinskaite, R. Cavanaugh, O. Evdokimov, L. Gauthier, C. E. Gerber, D. J. Hofman, S. Khalatyan, P. Kurt, D. H. Moon, C. O’Brien, C. Silkworth, P. Turner, N. Varelas, E. A. Albayrak, B. Bilki, W. Clarida, K. Dilsiz, F. Duru, M. Haytmyradov, J.-P. Merlo, H. Mermerkaya, A. Mestvirishvili, A. Moeller, J. Nachtman, H. Ogul, Y. Onel, F. Ozok, A. Penzo, R. Rahmat, S. Sen, P. Tan, E. Tiras, J. Wetzel, T. Yetkin, K. Yi, B. A. Barnett, B. Blumenfeld, S. Bolognesi, D. Fehling, A. V. Gritsan, P. Maksimovic, C. Martin, M. Swartz, P. Baringer, A. Bean, G. Benelli, C. Bruner, J. Gray, R. P. Kenny, M. Malek, M. Murray, D. Noonan, S. Sanders, J. Sekaric, R. Stringer, Q. Wang, J. S. Wood, A. F. Barfuss, I. Chakaberia, A. Ivanov, S. Khalil, M. Makouski, Y. Maravin, L. K. Saini, S. Shrestha, I. Svintradze, J. Gronberg, D. Lange, F. Rebassoo, D. Wright, A. Baden, A. Belloni, B. Calvert, S. C. Eno, J. A. Gomez, N. J. Hadley, R. G. Kellogg, T. Kolberg, Y. Lu, M. Marionneau, A. C. Mignerey, K. Pedro, A. Skuja, M. B. Tonjes, S. C. Tonwar, A. Apyan, R. Barbieri, G. Bauer, W. Busza, I. A. Cali, M. Chan, L. Di Matteo, V. Dutta, G. Gomez Ceballos, M. Goncharov, D. Gulhan, M. Klute, Y. S. Lai, Y.-J. Lee, A. Levin, P. D. Luckey, T. Ma, C. Paus, D. Ralph, C. Roland, G. Roland, G. S. F. Stephans, F. Stöckli, K. Sumorok, D. Velicanu, J. Veverka, B. Wyslouch, M. Yang, A. S. Yoon, M. Zanetti, V. Zhukova, B. Dahmes, A. De Benedetti, A. Gude, S. C. Kao, K. Klapoetke, Y. Kubota, J. Mans, N. Pastika, R. Rusack, A. Singovsky, N. Tambe, J. Turkewitz, J. G. Acosta, L. M. Cremaldi, R. Kroeger, S. Oliveros, L. Perera, D. A. Sanders, D. Summers, E. Avdeeva, K. Bloom, S. Bose, D. R. Claes, A. Dominguez, R. Gonzalez Suarez, J. Keller, D. Knowlton, I. Kravchenko, J. Lazo-Flores, S. Malik, F. Meier, G. R. Snow, J. Dolen, A. Godshalk, I. Iashvili, S. Jain, A. Kharchilava, A. Kumar, S. Rappoccio, G. Alverson, E. Barberis, D. Baumgartel, M. Chasco, J. Haley, A. Massironi, D. Nash, T. Orimoto, D. Trocino, R. j. Wang, D. Wood, J. Zhang, A. Anastassov, K. A. Hahn, A. Kubik, L. Lusito, N. Mucia, N. Odell, B. Pollack, A. Pozdnyakov, M. Schmitt, S. Stoynev, K. Sung, M. Velasco, S. Won, A. Brinkerhoff, K. M. Chan, A. Drozdetskiy, M. Hildreth, C. Jessop, D. J. Karmgard, N. Kellams, K. Lannon, W. Luo, S. Lynch, N. Marinelli, T. Pearson, M. Planer, R. Ruchti, N. Valls, M. Wayne, M. Wolf, A. Woodard, L. Antonelli, J. Brinson, B. Bylsma, L. S. Durkin, S. Flowers, C. Hill, R. Hughes, K. Kotov, T. Y. Ling, D. Puigh, M. Rodenburg, G. Smith, C. Vuosalo, B. L. Winer, H. Wolfe, H. W. Wulsin, O. Driga, P. Elmer, P. Hebda, A. Hunt, S. A. Koay, P. Lujan, D. Marlow, T. Medvedeva, M. Mooney, J. Olsen, P. Piroué, X. Quan, H. Saka, D. Stickland, C. Tully, J. S. Werner, S. C. Zenz, A. Zuranski, E. Brownson, H. Mendez, J. E. Ramirez Vargas, E. Alagoz, V. E. Barnes, D. Benedetti, G. Bolla, D. Bortoletto, M. De Mattia, Z. Hu, M. K. Jha, M. Jones, K. Jung, M. Kress, N. Leonardo, D. Lopes Pegna, V. Maroussov, P. Merkel, D. H. Miller, N. Neumeister, B. C. Radburn-Smith, X. Shi, I. Shipsey, D. Silvers, A. Svyatkovskiy, F. Wang, W. Xie, L. Xu, H. D. Yoo, J. Zablocki, Y. Zheng, N. Parashar, J. Stupak, A. Adair, B. Akgun, K. M. Ecklund, F. J. M. Geurts, W. Li, B. Michlin, B. P. Padley, R. Redjimi, J. Roberts, J. Zabel, B. Betchart, A. Bodek, R. Covarelli, P. de Barbaro, R. Demina, Y. Eshaq, T. Ferbel, A. Garcia-Bellido, P. Goldenzweig, J. Han, A. Harel, A. Khukhunaishvili, G. Petrillo, D. Vishnevskiy, R. Ciesielski, L. Demortier, K. Goulianos, G. Lungu, C. Mesropian, S. Arora, A. Barker, J. P. Chou, C. Contreras-Campana, E. Contreras-Campana, D. Duggan, D. Ferencek, Y. Gershtein, R. Gray, E. Halkiadakis, D. Hidas, A. Lath, S. Panwalkar, M. Park, R. Patel, S. Salur, S. Schnetzer, S. Somalwar, R. Stone, S. Thomas, P. Thomassen, M. Walker, K. Rose, S. Spanier, A. York, O. Bouhali, R. Eusebi, W. Flanagan, J. Gilmore, T. Kamon, V. Khotilovich, V. Krutelyov, R. Montalvo, I. Osipenkov, Y. Pakhotin, A. Perloff, J. Roe, A. Rose, A. Safonov, T. Sakuma, I. Suarez, A. Tatarinov, N. Akchurin, C. Cowden, J. Damgov, C. Dragoiu, P. R. Dudero, J. Faulkner, K. Kovitanggoon, S. Kunori, S. W. Lee, T. Libeiro, I. Volobouev, E. Appelt, A. G. Delannoy, S. Greene, A. Gurrola, W. Johns, C. Maguire, Y. Mao, A. Melo, M. Sharma, P. Sheldon, B. Snook, S. Tuo, J. Velkovska, M. W. Arenton, S. Boutle, B. Cox, B. Francis, J. Goodell, R. Hirosky, A. Ledovskoy, H. Li, C. Lin, C. Neu, J. Wood, R. Harr, P. E. Karchin, C. Kottachchi Kankanamge Don, P. Lamichhane, J. Sturdy, D. A. Belknap, D. Carlsmith, M. Cepeda, S. Dasu, S. Duric, E. Friis, R. Hall-Wilton, M. Herndon, A. Hervé, P. Klabbers, A. Lanaro, C. Lazaridis, A. Levine, R. Loveless, A. Mohapatra, I. Ojalvo, T. Perry, G. A. Pierro, G. Polese, I. Ross, T. Sarangi, A. Savin, W. H. Smith, N. Woods

**Affiliations:** 1 Yerevan Physics Institute, Yerevan, Armenia; 2 Institut für Hochenergiephysik der OeAW, Vienna, Austria; 3 National Centre for Particle and High Energy Physics, Minsk, Belarus; 4 Universiteit Antwerpen, Antwerp, Belgium; 5 Vrije Universiteit Brussel, Brussels, Belgium; 6 Université Libre de Bruxelles, Brussels, Belgium; 7 Ghent University, Ghent, Belgium; 8 Université Catholique de Louvain, Louvain-la-Neuve, Belgium; 9 Université de Mons, Mons, Belgium; 10 Centro Brasileiro de Pesquisas Fisicas, Rio de Janeiro, Brazil; 11 Universidade do Estado do Rio de Janeiro, Rio de Janeiro, Brazil; 12 Universidade Estadual Paulista, Universidade Federal do ABC, São Paulo, Brazil; 13 Institute for Nuclear Research and Nuclear Energy, Sofia, Bulgaria; 14 University of Sofia, Sofia, Bulgaria; 15 Institute of High Energy Physics, Beijing, China; 16 State Key Laboratory of Nuclear Physics and Technology, Peking University, Beijing, China; 17 Universidad de Los Andes, Bogotá, Colombia; 18 Technical University of Split, Split, Croatia; 19 University of Split, Split, Croatia; 20 Institute Rudjer Boskovic, Zagreb, Croatia; 21 University of Cyprus, Nicosia, Cyprus; 22 Charles University, Prague, Czech Republic; 23 Academy of Scientific Research and Technology of the Arab Republic of Egypt, Egyptian Network of High Energy Physics, Cairo, Egypt; 24 National Institute of Chemical Physics and Biophysics, Tallinn, Estonia; 25 Department of Physics, University of Helsinki, Helsinki, Finland; 26 Helsinki Institute of Physics, Helsinki, Finland; 27 Lappeenranta University of Technology, Lappeenranta, Finland; 28 DSM/IRFU, CEA/Saclay, Gif-sur-Yvette, France; 29 Laboratoire Leprince-Ringuet, Ecole Polytechnique, IN2P3-CNRS, Palaiseau, France; 30 Institut Pluridisciplinaire Hubert Curien, Université de Strasbourg, Université de Haute Alsace Mulhouse, CNRS/IN2P3, Strasbourg, France; 31 Centre de Calcul de l’Institut National de Physique Nucleaire et de Physique des Particules, CNRS/IN2P3, Villeurbanne, France; 32 Institut de Physique Nucléaire de Lyon, Université de Lyon, Université Claude Bernard Lyon 1, CNRS-IN2P3, Villeurbanne, France; 33 Institute of High Energy Physics and Informatization, Tbilisi State University, Tbilisi, Georgia; 34 RWTH Aachen University, I. Physikalisches Institut, Aachen, Germany; 35 RWTH Aachen University, III. Physikalisches Institut A, Aachen, Germany; 36 RWTH Aachen University, III. Physikalisches Institut B, Aachen, Germany; 37 Deutsches Elektronen-Synchrotron, Hamburg, Germany; 38 University of Hamburg, Hamburg, Germany; 39 Institut für Experimentelle Kernphysik, Karlsruhe, Germany; 40 Institute of Nuclear and Particle Physics (INPP), NCSR Demokritos, Aghia Paraskevi, Greece; 41 University of Athens, Athens, Greece; 42 University of Ioánnina, Ioannina, Greece; 43 Wigner Research Centre for Physics, Budapest, Hungary; 44 Institute of Nuclear Research ATOMKI, Debrecen, Hungary; 45 University of Debrecen, Debrecen, Hungary; 46 National Institute of Science Education and Research, Bhubaneswar, India; 47 Panjab University, Chandigarh, India; 48 University of Delhi, Delhi, India; 49 Saha Institute of Nuclear Physics, Kolkata, India; 50 Bhabha Atomic Research Centre, Mumbai, India; 51 Tata Institute of Fundamental Research, Mumbai, India; 52 Institute for Research in Fundamental Sciences (IPM), Tehran, Iran; 53 University College Dublin, Dublin, Ireland; 54 INFN Sezione di Bari, Università di Bari, Politecnico di Bari, Bari, Italy; 55 INFN Sezione di Bologna, Università di Bologna, Bologna, Italy; 56 INFN Sezione di Catania, Università di Catania, CSFNSM, Catania, Italy; 57 INFN Sezione di Firenze, Università di Firenze, Florence, Italy; 58 INFN Laboratori Nazionali di Frascati, Frascati, Italy; 59 INFN Sezione di Genova, Università di Genova, Genoa, Italy; 60 INFN Sezione di Milano-Bicocca, Università di Milano-Bicocca, Milan, Italy; 61 INFN Sezione di Napoli, Università di Napoli ’Federico II’, Università della Basilicata (Potenza), Università G. Marconi (Roma), Naples, Italy; 62 INFN Sezione di Padova, Università di Padova, Università di Trento (Trento), Padua, Italy; 63 INFN Sezione di Pavia, Università di Pavia, Pavia, Italy; 64 INFN Sezione di Perugia, Università di Perugia, Perugia, Italy; 65 INFN Sezione di Pisa, Università di Pisa, Scuola Normale Superiore di Pisa, Pisa, Italy; 66 INFN Sezione di Roma, Università di Rome, Rome, Italy; 67 INFN Sezione di Torino, Università di Torino, Università del Piemonte Orientale (Novara), Turin, Italy; 68 INFN Sezione di Trieste, Università di Trieste, Trieste, Italy; 69 Chonbuk National University, Chonju, Korea; 70 Kangwon National University, Chunchon, Korea; 71 Kyungpook National University, Daegu, Korea; 72 Chonnam National University, Institute for Universe and Elementary Particles, Kwangju, Korea; 73 Korea University, Seoul, Korea; 74 University of Seoul, Seoul, Korea; 75 Sungkyunkwan University, Suwon, Korea; 76 Vilnius University, Vilnius, Lithuania; 77 National Centre for Particle Physics, Universiti Malaya, Kuala Lumpur, Malaysia; 78 Centro de Investigacion y de Estudios Avanzados del IPN, Mexico City, Mexico; 79 Universidad Iberoamericana, Mexico City, Mexico; 80 Benemerita Universidad Autonoma de Puebla, Puebla, Mexico; 81 Universidad Autónoma de San Luis Potosí, San Luis Potosí, Mexico; 82 University of Auckland, Auckland, New Zealand; 83 University of Canterbury, Christchurch, New Zealand; 84 National Centre for Physics, Quaid-I-Azam University, Islamabad, Pakistan; 85 National Centre for Nuclear Research, Swierk, Poland; 86 Institute of Experimental Physics, Faculty of Physics, University of Warsaw, Warsaw, Poland; 87 Laboratório de Instrumentação e Física Experimental de Partículas, Lisbon, Portugal; 88 Joint Institute for Nuclear Research, Dubna, Russia; 89 Petersburg Nuclear Physics Institute, Gatchina (St. Petersburg), Russia; 90 Institute for Nuclear Research, Moscow, Russia; 91 Institute for Theoretical and Experimental Physics, Moscow, Russia; 92 P. N. Lebedev Physical Institute, Moscow, Russia; 93 Skobeltsyn Institute of Nuclear Physics, Lomonosov Moscow State University, Moscow, Russia; 94 State Research Center of Russian Federation, Institute for High Energy Physics, Protvino, Russia; 95 University of Belgrade, Faculty of Physics and Vinca Institute of Nuclear Sciences, Belgrade, Serbia; 96 Centro de Investigaciones Energéticas Medioambientales y Tecnológicas (CIEMAT), Madrid, Spain; 97 Universidad Autónoma de Madrid, Madrid, Spain; 98 Universidad de Oviedo, Oviedo, Spain; 99 Instituto de Física de Cantabria (IFCA), CSIC-Universidad de Cantabria, Santander, Spain; 100 CERN, European Organization for Nuclear Research, Geneva, Switzerland; 101 Paul Scherrer Institut, Villigen, Switzerland; 102 Institute for Particle Physics, ETH Zurich, Zurich, Switzerland; 103 Universität Zürich, Zurich, Switzerland; 104 National Central University, Chung-Li, Taiwan; 105 National Taiwan University (NTU), Taipei, Taiwan; 106 Chulalongkorn University, Faculty of Science, Department of Physics, Bangkok, Thailand; 107 Cukurova University, Adana, Turkey; 108 Physics Department, Middle East Technical University, Ankara, Turkey; 109 Bogazici University, Istanbul, Turkey; 110 Istanbul Technical University, Istanbul, Turkey; 111 National Scientific Center, Kharkov Institute of Physics and Technology, Kharkov, Ukraine; 112 University of Bristol, Bristol, UK; 113 Rutherford Appleton Laboratory, Didcot, UK; 114 Imperial College, London, UK; 115 Brunel University, Uxbridge, UK; 116 Baylor University, Waco, USA; 117 The University of Alabama, Tuscaloosa, USA; 118 Boston University, Boston, USA; 119 Brown University, Providence, USA; 120 University of California, Davis, USA; 121 University of California, Los Angeles, USA; 122 University of California, Riverside, Riverside, USA; 123 University of California, San Diego, La Jolla, USA; 124 University of California, Santa Barbara, Santa Barbara, USA; 125 California Institute of Technology, Pasadena, USA; 126 Carnegie Mellon University, Pittsburg, USA; 127 University of Colorado at Boulder, Boulder, USA; 128 Cornell University, Ithaca, USA; 129 Fairfield University, Fairfield, USA; 130 Fermi National Accelerator Laboratory, Batavia, USA; 131 University of Florida, Gainesville, USA; 132 Florida International University, Miami, USA; 133 Florida State University, Tallahassee, USA; 134 Florida Institute of Technology, Melbourne, USA; 135 University of Illinois at Chicago (UIC), Chicago, USA; 136 The University of Iowa, Iowa City, USA; 137 Johns Hopkins University, Baltimore, USA; 138 The University of Kansas, Lawrence, USA; 139 Kansas State University, Manhattan, USA; 140 Lawrence Livermore National Laboratory, Livermore, USA; 141 University of Maryland, College Park, USA; 142 Massachusetts Institute of Technology, Cambridge, USA; 143 University of Minnesota, Minneapolis, USA; 144 University of Mississippi, Oxford, USA; 145 University of Nebraska-Lincoln, Lincoln, USA; 146 State University of New York at Buffalo, Buffalo, USA; 147 Northeastern University, Boston, USA; 148 Northwestern University, Evanston, USA; 149 University of Notre Dame, Notre Dame, USA; 150 The Ohio State University, Columbus, USA; 151 Princeton University, Princeton, USA; 152 University of Puerto Rico, Mayagüez, USA; 153 Purdue University, West Lafayette, USA; 154 Purdue University Calumet, Hammond, USA; 155 Rice University, Houston, USA; 156 University of Rochester, Rochester, USA; 157 The Rockefeller University, New York, USA; 158 Rutgers, The State University of New Jersey, Piscataway, USA; 159 University of Tennessee, Knoxville, USA; 160 Texas A&M University, College Station, USA; 161 Texas Tech University, Lubbock, USA; 162 Vanderbilt University, Nashville, USA; 163 University of Virginia, Charlottesville, USA; 164 Wayne State University, Detroit, USA; 165 University of Wisconsin, Madison, USA; 166 CERN, 1211 Geneva 23, Switzerland

## Abstract

A search for heavy, right-handed neutrinos, $\mathrm {N}_{\ell }$ ($\ell = \mathrm {e}, \mu$), and right-handed $\mathrm {W}_{\mathrm {R}}$ bosons, which arise in the left-right symmetric extensions of the standard model, has been performed by the CMS experiment. The search was based on a sample of two lepton plus two jet events collected in proton–proton collisions at a center-of-mass energy of 8$\,\text {TeV}$ corresponding to an integrated luminosity of 19.7 $\mathrm{fb}^{-1}$. For models with strict left-right symmetry, and assuming only one $\mathrm {N}_{\ell }$ flavor contributes significantly to the $\mathrm {W}_{\mathrm {R}}$ decay width, the region in the two-dimensional $(M_{\mathrm {W}_{\mathrm {R}}}, M_{\mathrm {N}_{\ell }})$ mass plane excluded at a 95 % confidence level extends to approximately $M_{\mathrm {W}_{\mathrm {R}}} = 3.0\,\text {TeV}$ and covers a large range of neutrino masses below the $\mathrm {W}_{\mathrm {R}}$ boson mass, depending on the value of $M_{\mathrm {W}_{\mathrm {R}}}$. This search significantly extends the $(M_{\mathrm {W}_{\mathrm {R}}}, M_{\mathrm {N}_{\ell }})$ exclusion region beyond previous results.

**Electronic supplementary material:**

The online version of this article (doi:10.1140/epjc/s10052-014-3149-z) contains supplementary material, which is available to authorized users.

## Introduction

The standard model (SM) [1–3] explicitly incorporates the parity violation observed in weak interactions through the use of a left-handed chiral $SU_{L}(2)$ gauge group which includes the left-handed gauge bosons $\mathrm {W}_{\mathrm {L}} ^{\pm }$ and $\mathrm {Z}_{\mathrm {L}}$. One of the attractive features of left-right (LR) symmetric extensions [4–7] to the standard model is that these models explain parity violation in the SM as the consequence of spontaneous symmetry breaking of a larger gauge group to $SU_{L}(2) \times SU_{R}(2)$ at a multi-TeV mass scale. The LR extensions introduce an additional right-handed $SU_{R}(2)$ symmetry group to the SM, which includes heavy charged ($\mathrm {W}_{\mathrm {R}} ^{\pm }$) and neutral ($\mathrm {Z}_{\mathrm {R}}$) gauge bosons that could be produced at LHC energies.

In addition to addressing parity non-conservation in weak interactions, LR theories also provide an explanation for the mass of SM neutrinos. The observation of neutrino oscillations [8, 9] requires that neutrinos have mass, and the fact that the neutrino mass scale [10] is far below that of quarks and charged leptons suggests that the origin of neutrino mass may be distinct from the origin of mass for the other SM fermions. Heavy right-handed Majorana neutrinos ($\mathrm {N}_{\mathrm {e}}$, $\mathrm {N}_{\mathrm {\mu }}$, and $\mathrm {N}_{\tau }$), which are naturally present in LR models, provide a possible explanation for the mass of SM neutrinos through the see-saw mechanism [11, 12].

We search for $\mathrm {W}_{\mathrm {R}}$ bosons produced in a sample of proton–proton collisions at a center-of-mass energy $\sqrt{s} = 8$ $\,\text {TeV}$ and collected by the CMS detector at the CERN LHC. This search, which expands upon a previous search using $\sqrt{s} = 7$ $\,\text {TeV}$ data [13], assumes the production of a $\mathrm {W}_{\mathrm {R}}$ boson that decays to a charged lepton (we consider $\ell = \mathrm {e}, \mu$) and to a right-handed neutrino $\mathrm {N}_{\ell }$. The decay of the right-handed neutrino produces a second charged lepton of the same flavor together with a virtual right-handed charged boson $\mathrm {W}_{\mathrm {R}} ^{*}$. When the $\mathrm {W}_{\mathrm {R}} ^{*}$ decays to a pair of quarks, we arrive at the decay chain:

$$\begin{aligned} \mathrm {W}_{\mathrm {R}} \rightarrow \ell _1 \mathrm {N}_{\ell } \rightarrow \ell _1 \ell _2 \mathrm {W}_{\mathrm {R}} ^* \rightarrow \ell _1 \ell _2 \mathrm {q}\overline{\mathrm {q}}. \end{aligned}$$

The quarks hadronize into jets ($j$), resulting in an observable final state containing two same-flavor charged leptons and two jets. Although the potential Majorana nature of the right-handed neutrinos implies the final-state charged leptons can have the same sign, we do not impose any charge requirements on the final-state electrons or muons in this analysis.

This search is characterized by the masses of the $\mathrm {W}_{\mathrm {R}}$ boson ($M_{\mathrm {W}_{\mathrm {R}}}$) and the right-handed neutrino $\mathrm {N}_{\ell }$ ($M_{\mathrm {N}_{\ell }}$), which are allowed to vary independently. Although $M_{\mathrm {N}_{\ell }} > M_{\mathrm {W}_{\mathrm {R}}}$ is allowed in the LR symmetric model, it is not considered in this analysis in favor of the dominant $\mathrm {q}\overline{\mathrm {q}}' \rightarrow \mathrm {W}_{\mathrm {R}}$ production mechanism. As the branching fraction for $\mathrm {W}_{\mathrm {R}} \rightarrow \ell \mathrm {N}_{\ell }$ depends on the number of heavy-neutrino flavors accessible at LHC energies, results are first interpreted assuming that only one neutrino flavor, namely $\mathrm {N}_{\mathrm {e}}$ or $\mathrm {N}_{\mathrm {\mu }}$, is light enough to contribute significantly to the $\mathrm {W}_{\mathrm {R}}$ boson decay width. Results are then interpreted assuming degenerate $\mathrm {N}_{\mathrm {e}}$, $\mathrm {N}_{\mathrm {\mu }}$, and $\mathrm {N}_{\tau }$ masses.

For given $\mathrm {W}_{\mathrm {R}}$ boson and $\mathrm {N}_{\ell }$ mass assumptions, the signal cross section can be predicted from the value of the coupling constant $g_R$, which denotes the strength of the gauge interactions of $\mathrm {W}_{\mathrm {R}}$ bosons. We assume strict LR symmetry, such that $g_R$ is equal to the (left-handed) weak interaction coupling strength $g_L$ at $M_{\mathrm {W}_{\mathrm {R}}}$, and we also assume identical quark and neutrino mixing matrices for the left- and right-handed interactions. The $\mathrm {W}_{\mathrm {R}}$ boson production cross section can then be calculated by the fewz program [14] using the left-handed $\mathrm {W^{'}}$ model [15]. Finally, the left-right boson and lepton mixing angles are assumed to be small [16].

The theoretical lower limit on $\mathrm {W}_{\mathrm {R}}$ mass of $M_{\mathrm {W}_{\mathrm {R}}} \gtrsim 2.5$ $\,\text {TeV}$  [17, 18] is estimated from the measured size of the $\mathrm {K}_{\mathrm {L}} \text {--} \mathrm {K}_{\mathrm {S}}$ mass difference. Searches for $\mathrm {W}_{\mathrm {R}} \rightarrow \mathrm {t}\overline{\mathrm {b}}$ decays at the LHC using $\sqrt{s} = 7$ and 8$\,\text {TeV}$ data [19–21] have excluded $\mathrm {W}_{\mathrm {R}}$ boson masses below 2.05$\,\text {TeV}$ at 95 % confidence level (CL), and previous searches for $\mathrm {W}_{\mathrm {R}} \rightarrow \ell \mathrm {N}_{\ell }$ at the LHC excluded at 95 % CL a region in the two-dimensional parameter space $(M_{\mathrm {W}_{\mathrm {R}}},M_{\mathrm {N}_{\ell }})$ extending to nearly $M_{\mathrm {W}_{\mathrm {R}}} = 2.5$ $\,\text {TeV}$  [13, 22]. This paper describes the first direct search that is sensitive to $M_{\mathrm {W}_{\mathrm {R}}}$ values beyond the theoretical lower mass limit.

## The CMS detector

The central feature of the CMS apparatus is a superconducting solenoid, of 6 m internal diameter, providing a field of 3.8 T. Within the field volume are the silicon pixel and strip tracker, the PbWO$_4$ crystal electromagnetic calorimeter (ECAL) and the brass and scintillator hadron calorimeter (HCAL). Muons are measured in gas-ionization detectors embedded in the steel flux-return yoke. The ECAL has an energy resolution of better than 0.5 % for unconverted photons with transverse energies $E_{\mathrm {T}} \equiv E {/} \cosh \eta > 100$ $\,\text {GeV}$. The muons are measured in the pseudorapidity window $|\eta |< 2.4$, where $\eta = -\ln [\tan (\theta /2)]$ and $\theta$ is the polar angle with respect to the counterclockwise-beam direction. The muon system detection planes are made of three technologies: drift tubes, cathode strip chambers, and resistive-plate chambers. Matching the muons to the tracks measured in the silicon tracker results in a transverse momentum ($p_{\mathrm {T}} \equiv | p | {/} \cosh \eta$) resolution between 1 and 10 % for $p_{\mathrm {T}} < 1$ $\,\text {TeV}$. The inner tracker measures charged particles within the range $|\eta | < 2.5$ and provides an impact parameter resolution of $\sim$15 $\upmu$m and a $p_{\mathrm {T}}$ resolution of about 1.5 % for 100$\,\text {GeV}$ particles. The first level of the CMS trigger system, composed of custom hardware processors, uses information from the calorimeters and muon detectors to select up to 100 kHz of events of interest. The high-level trigger (HLT) processor farm uses information from all CMS subdetectors to further decrease the event rate to about 400 Hz before data storage. A more detailed description of the CMS detector, together with a definition of the coordinate system used and the relevant kinematic variables, can be found elsewhere [23].

The particle-flow event reconstruction technique [24, 25] used to reconstruct jets in this analysis consists in reconstructing and identifying each single particle with an optimized combination of all subdetector information. The energy of photons is directly obtained from the ECAL measurement, corrected for zero-suppression effects. The energy of electrons is determined from a combination of the track momentum at the main interaction vertex, the corresponding ECAL cluster energy, and the energy sum of all bremsstrahlung photons attached to the track. The energy of muons is obtained from the corresponding track momentum. The energy of charged hadrons is determined from a combination of the track momentum and the corresponding ECAL and HCAL energy, corrected for zero-suppression effects and for the response function of the calorimeters to hadronic showers. Finally, the energy of neutral hadrons is obtained from the corresponding corrected ECAL and HCAL energy.

## Data and Monte Carlo samples

The search for $\mathrm {W}_{\mathrm {R}}$ boson production described in this paper is performed using pp collision data collected with the CMS detector at $\sqrt{s} = 8$ $\,\text {TeV}$ in 2012. The data sample corresponds to an integrated luminosity of 19.7 $\mathrm{fb}^{-1}$. Candidate $\mathrm {W}_{\mathrm {R}} \rightarrow \mathrm {e}\mathrm {N}_{\mathrm {e}}$ events are collected using a double-electron trigger that requires two clusters in ECAL with $E_{\mathrm {T}} > 33$ $\,\text {GeV}$ each. These ECAL clusters are loosely matched at the HLT stage to tracks formed from hits in the pixel detector. To reject hadronic backgrounds, only a small amount of energy in the HCAL may be associated with the HLT electron candidates. Muon channel events are selected with a single-muon trigger that requires at least one candidate muon with $p_{\mathrm {T}} > 40$ $\,\text {GeV}$ and $|\eta |< 2.1$, as reconstructed by the HLT.

Simulated $\mathrm {W}_{\mathrm {R}} \rightarrow \ell \mathrm {N}_{\ell }$ signal samples are generated assuming $M_{\mathrm {N}_{\ell }} = \frac{1}{2} M_{\mathrm {W}_{\mathrm {R}}}$ using pythia 6.4.26 [26], a tree-level Monte Carlo (MC) generator, with CTEQ6L1 parton distribution functions (PDF) [27] and underlying event tune Z2* [28]. The MC generator includes the LR symmetric model with the assumptions previously mentioned. The final state leptons and jets in these signal events are sufficiently energetic to allow reconstruction effects to be addressed apart from the kinematic requirements discussed below. With this separation, the extension from $M_{\mathrm {N}_{\ell }} = \frac{1}{2} M_{\mathrm {W}_{\mathrm {R}}}$ to the full two-dimensional $(M_{\mathrm {W}_{\mathrm {R}}},M_{\mathrm {N}_{\ell }})$ mass plane for signal events is straight-forward, as is discussed in Sect. 7. The dominant backgrounds to $\mathrm {W}_{\mathrm {R}}$ boson production include SM processes with at least two charged leptons with large transverse momentum, namely $\mathrm {t}\overline{\mathrm {t}} \rightarrow \mathrm {b}\mathrm {W^+}\overline{\mathrm {b}}\mathrm {W^-}$ and Drell–Yan (DY)+jets processes. All remaining SM background events, which collectively contribute less than 10 % to the total background level, are dominated by diboson and single top quark processes. The $\mathrm {t}\overline{\mathrm {t}}$ background is estimated using control samples in data and a simulated sample of fully leptonic $\mathrm {t}\overline{\mathrm {t}}$ decays, which are generated using the tree-level matrix element MC generator MadGraph 5.1.4.8 [29]. The DY+jets background is estimated using exclusive DY+n jets ($n=0$, 1, 2, 3, 4) simulated samples generated with MadGraph 5.1.3.30. For the above MadGraph samples, parton showering, fragmentation, and the underlying event are handled by pythia. A statistically comparable sample of DY+jets events generated with the tree-level MC event generator sherpa 1.4.2 [30], which incorporates parton showering and other effects in addition to the hard process, is used to help quantify the systematic uncertainty in the DY+jets background estimation. Simulated diboson ($\mathrm {W}\mathrm {W}$, $\mathrm {W}$Z, and ZZ) events are generated using pythia 6.4.26, with the additional small contributions from diboson scattering processes generated with MadGraph 5.1.3.30. The simulated single top quark (namely, tW) background sample is generated via the next-to-leading-order MC generator powheg 1.0 [31–34]. Parton showering and other effects are handled by pythia for the diboson and single top quark background samples.

The generated signal and SM background events pass through a full CMS detector simulation based on Geant4 [35], and are reconstructed with the same software used to reconstruct collision data, unless otherwise noted. The simulation is compared to data using various control samples, and when necessary the simulation is adjusted to account for slight deviations seen with respect to data. Additional pp collisions in the same beam crossing (pileup) are also included for each simulated event to realistically describe the $\sqrt{s} = 8$ $\,\text {TeV}$ collision environment.

## Event selection and object reconstruction

We assemble $\mathrm {W}_{\mathrm {R}}$ boson candidates from the two highest-$p_{\mathrm {T}}$ (leading) jets and two highest-$p_{\mathrm {T}}$ same-flavor leptons (electrons or muons) reconstructed in collision data or simulation events. Candidate events are first selected by the CMS trigger system using the lepton triggers described previously. The electron and muon trigger efficiencies are determined using the “tag and probe” techniques applied to $\mathrm {Z}\rightarrow \ell \ell$ candidates [36–38]. Simple triggers, requiring a single ECAL cluster with $E_{\mathrm {T}} > 300$ $\,\text {GeV}$, collected events with high-$p_{\mathrm {T}}$ electrons to help evaluate the trigger efficiency for electron channel events with high dielectron mass [39]. Following the application of object and event selection requirements mentioned below, the trigger efficiency for $\mathrm {W}_{\mathrm {R}} \rightarrow \ell \mathrm {N}_{\ell }$ candidate events is greater than 99 % (98 %) in the electron (muon) channel.

Because of the large expected mass of the $\mathrm {W}_{\mathrm {R}}$ boson, electron and muon reconstruction and identification are performed using algorithms optimized for objects with large transverse momentum [36, 39]. Non-isolated muon backgrounds are suppressed by computing the transverse momentum sum of all additional tracks within a cone of $\Delta R < 0.3$ about the muon direction, where $\Delta R = \sqrt{{(\Delta \eta )^{2} + (\Delta \phi )^{2}}}$ (azimuthal angle $\phi$ in radians), and requiring the $p_{\mathrm {T}}$ sum to be less than 10 % of the muon transverse momentum. This isolation requirement is only weakly dependent on the number of pileup collisions in the event, as tracks with a large $\Delta {z}$ separation from the muon, i.e., tracks from other $\mathrm {p}\mathrm {p}$ collisions, are not included in the isolation sum. Electrons are expected to have minimal associated HCAL energy and also to appear isolated in both calorimeters and in the tracker. To minimize the effects of pileup, electrons must be associated with the primary vertex, which is the collision vertex with the highest $\sum p_{\mathrm {T}} ^{2}$ of all associated tracks. As pileup collisions also produce extra energy in the calorimeters and can make the electron appear non-isolated, calorimeter isolation for electron candidates is corrected for the average energy density in the event [40].

**Table 1:** The total numbers of events reconstructed in data, and the expected contributions from signal and background samples, after successive stages of the selection requirements are applied. For the first selection stage, all kinematic and identification requirements are imposed on the leptons and jets as described in the text. The “Signal” column indicates the expected contribution for $M_{\mathrm {W}_{\mathrm {R}}} = 2.5$ $\,\text {TeV}$, with $M_{\mathrm {N}_{\ell }} = 1.25$ $\,\text {TeV}$. The “Other” column represents the combined background contribution from diboson and single top quark processes. The uncertainties in the background expectation are derived for the final stage of selection and more details are given in Sect. 6. The total experimental uncertainty is summarized in the first signal uncertainty, and the second signal uncertainty represents the PDF cross section uncertainty. The yields from earlier stages of the selection have greater relative uncertainty than that for the final $M_{\ell \ell j j} > 600$ $\,\text {GeV}$ selection stage

	Data	Signal	SM Backgrounds			
			Total	$\mathrm {t}\overline{\mathrm {t}}$	DY+jets	Other
Two electrons, two jets	34506	30	34154	4725	28273	1156
$M_{\mathrm {e}\mathrm {e}} > 200$ $\,\text {GeV}$	1717	29	1747	1164	475	108
$M_{\mathrm {e}\mathrm {e}j j} > 600$ $\,\text {GeV}$	817	$29 \pm 1 \pm 3$	$783 \pm 51$	$476 \pm 42$	$252 \pm 24$	$55 \pm 12$
Two muons, two jets	42090	35	41204	5625	34220	1359
$M_{\mathrm {\mu }\mathrm {\mu }} > 200$ $\,\text {GeV}$	2042	35	2064	1382	549	133
$M_{\mathrm {\mu }\mathrm {\mu }j j} > 600$ $\,\text {GeV}$	951	$35 \pm 1 \pm 4$	$913 \pm 58$	$562 \pm 50$	$287 \pm 26$	$64 \pm 12$

Jets are reconstructed using the anti-$k_{\mathrm {T}}$ clustering algorithm [41] with a distance parameter of 0.5. Charged and neutral hadrons, photons, and leptons reconstructed with the CMS particle-flow technique are used as input to the jet clustering algorithm. To reduce the contribution to jet energy from pileup collisions, charged hadrons that do not originate from the primary vertex in the event are not used in jet clustering. After jet clustering, the pileup calorimeter energy contribution from neutral particles is removed by applying a residual average area-based correction [40, 42]. Jet identification requirements [43] suppress jets from calorimeter noise and beam halo, and the event is rejected if either of the two highest-$p_{\mathrm {T}}$ jet candidates fails the identification criteria. The jet four-momenta are corrected for zero-suppression effects and for the response function of the calorimeters to hadronic showers based on studies with simulation and data [44]. As the electrons and muons from $\mathrm {W}_{\mathrm {R}}$ boson decay are likely to be spatially separated from jets in the detector, we reject any lepton found within a cone of radius $\Delta R < 0.5$ from the jet axis for either of the two leading jets.

After selecting jets and isolated electrons or muons in the event, $\mathrm {W}_{\mathrm {R}} \rightarrow \ell \mathrm {N}_{\ell }$ candidates are formed using the two leading same-flavor leptons and the two leading jets that satisfy the selection criteria. The leading (subleading) lepton is required to have $p_{\mathrm {T}} > 60\;(40)$ $\,\text {GeV}$, while the $p_{\mathrm {T}}$ of each jet candidate must exceed 40$\,\text {GeV}$. Electrons and jets are reconstructed within the tracker acceptance ($|\eta |< 2.5$). Muon acceptance extends to $|\eta |< 2.4$, although at least one muon is restricted to $|\eta |< 2.1$ in order to be selected by the trigger.

We perform a shape-based analysis, searching for evidence of $\mathrm {W}_{\mathrm {R}}$ boson production using the four-object mass distribution ($M_{\ell \ell j j}$), where we consider events with $M_{\ell \ell j j} > 600$ $\,\text {GeV}$. To reduce the contribution from DY+jets and other SM backgrounds, we also impose a requirement of $M_{\ell \ell } > 200$ $\,\text {GeV}$ on the mass of the lepton pair associated with the $\mathrm {W}_{\mathrm {R}}$ boson candidate.

The decay of a $\mathrm {W}_{\mathrm {R}}$ boson tends to produce final-state objects that have high $p_{\mathrm {T}}$ and are separated in the detector. We define the signal acceptance to include the kinematic and detector acceptance requirements for the leptons and jets, lepton-jet separation, and the minimum $M_{\ell \ell }$ and $M_{\ell \ell j j}$ requirements. This signal acceptance, typically near 80 % at $M_{\mathrm {N}_{\ell }} \sim M_{\mathrm {W}_{\mathrm {R}}}/2$, varies by less than 1 % between the electron and muon channels because of differences in detector acceptance for leptons. Provided that the $\mathrm {W}_{\mathrm {R}}$ boson decay satisfies acceptance requirements, the ability to reconstruct all four final-state particles is near 75 % 2.8 (85 %) for the electron (muon) channel, with some dependence on $\mathrm {W}_{\mathrm {R}}$ boson and $\mathrm {N}_{\ell }$ masses. However, if the mass of the $\mathrm {W}_{\mathrm {R}}$ boson is sufficiently heavy compared to that of the right-handed neutrino, the $\mathrm {N}_{\ell } \rightarrow \ell jj$ decay products tend to overlap and it becomes difficult to reconstruct two distinct jets or find leptons outside of the jet cone. As a result, the signal acceptance as a function of $M_{\mathrm {N}_{\ell }}$ decreases rapidly as $M_{\mathrm {N}_{\ell }}$ drops below about 10 % of the $\mathrm {W}_{\mathrm {R}}$ boson mass.

## Standard model backgrounds

The $\mathrm {t}\overline{\mathrm {t}}$ background contribution to the $\mathrm {e}\mathrm {e}jj$ and $\mu \mu jj$ final states is estimated using a control sample of $\mathrm {e}\mu jj$ events reconstructed in data. Studies of simulated $\mathrm {t}\overline{\mathrm {t}} \rightarrow \mathrm {e}\mathrm {e}jj$, $\mu \mu jj$, and $\mathrm {e}\mu jj$ decays confirm that the $M_{\mathrm {e}\mathrm {e}j j}$ and $M_{\mathrm {\mu }\mathrm {\mu }j j}$ distributions can be modeled by the $M_{\mathrm {e}\mathrm {\mu }j j}$ distribution, so we apply selection requirements to $\mathrm {e}\mu jj$ events that parallel those applied to electron and muon channel events. The $\mathrm {e}\mu jj$ events are collected using the same HLT selection as $\mu \mu j j$ events, although in this case only one muon is available for selection by the trigger. This sample is dominated by $\mathrm {t}\overline{\mathrm {t}}$ events, and small contributions from other SM processes are subtracted using simulation. The relative fractions of $\mathrm {t}\overline{\mathrm {t}} \rightarrow \mathrm {e}\mathrm {e}jj$, $\mu \mu jj$, and $\mathrm {e}\mu jj$ events that pass the selection criteria are determined from simulation. Using this information, the $M_{\mathrm {e}\mathrm {\mu }j j}$ distribution for the $\mathrm {e}\mu jj$ control sample from data is scaled to match the expected $\mathrm {t}\overline{\mathrm {t}}$ background contribution to the $M_{\mathrm {e}\mathrm {e}j j}$ and $M_{\mathrm {\mu }\mathrm {\mu }j j}$ distributions. The scale factor derived from simulation is determined after requiring $M_{\mathrm {e}\mu } > 200$ $\,\text {GeV}$ and $M_{\mathrm {e}\mathrm {\mu }j j} > 600$ $\,\text {GeV}$, which is equivalent to the third and final selection stage in Table 1. The scale factors for the $\mathrm {t}\overline{\mathrm {t}}$ background sample are $0.524 \pm 0.007$ and $0.632 \pm 0.008$ in the electron and muon channels, respectively, where the uncertainty in the values reflects the number of simulated $\mathrm {t}\overline{\mathrm {t}}$ events that satisfy all object and event requirements. The trigger efficiency for $\mathrm {e}\mu j j$ events is over 90 % for events with central muons ($|\eta |< 0.9$) and decreases for events with more forward muons. Consequently, both the electron and muon scale factors are larger than the expected value of 0.5, given the relative branching fractions for $\mathrm {t}\overline{\mathrm {t}} \rightarrow \mathrm {e}\mathrm {e}jj$, $\mu \mu jj$, and $\mathrm {e}\mu jj$ decays.

The $\mathrm {t}\overline{\mathrm {t}}$ scale factors, determined from simulation, are checked using control regions in data. We first consider events in both simulation and data where one or both jets are identified as originating from a bottom quark. After all selection requirements are applied, reconstructed $\mathrm {t}\overline{\mathrm {t}}$ decays dominate the event samples. Accounting for contributions from other SM processes using simulation, we compute scale factors for $\mathrm {e}\mu j j$ events in data with $60 < M_{\mathrm {e}\mu } < 200$ $\,\text {GeV}$ to estimate the $\mathrm {t}\overline{\mathrm {t}}$ contribution to the SM background when one or both jets are tagged as b jets using the medium working point of the combined secondary vertex tagging algorithm [45]. The $M_{\mathrm {e}\mathrm {e}}$ and $M_{\mathrm {\mu }\mathrm {\mu }}$ distributions in b-tagged data agree with expectations based on simulation and the $\mathrm {e}\mu j j$ control sample, and the derived scale factors agree with those obtained from simulation within statistical precision. For another cross-check, we compute the scale factor based on the expectation that $\mathrm {t}\overline{\mathrm {t}} \rightarrow \mathrm {e}\mu j j$ should be twice the rate of $\mathrm {t}\overline{\mathrm {t}} \rightarrow \mathrm {e}\mathrm {e}jj$ or $\mathrm {t}\overline{\mathrm {t}} \rightarrow \mu \mu jj$. Deviation from this expected ratio depends primarily on the differences in electron and muon reconstruction and identification efficiencies. The number of electron and muon channel events in data in the $120 < M_{\ell \ell } < 200\,\text {GeV}$ control region are thus used to derive the relative efficiency difference between electrons and muons and then extract the $\mathrm {t}\overline{\mathrm {t}}$ scale factors. The scale factors determined from this control region in data are consistent with those derived from simulation, and the larger statistical uncertainty (2 %) of this cross-check is taken as the systematic uncertainty in the $\mathrm {t}\overline{\mathrm {t}}$ normalization.

**Fig. 1:**
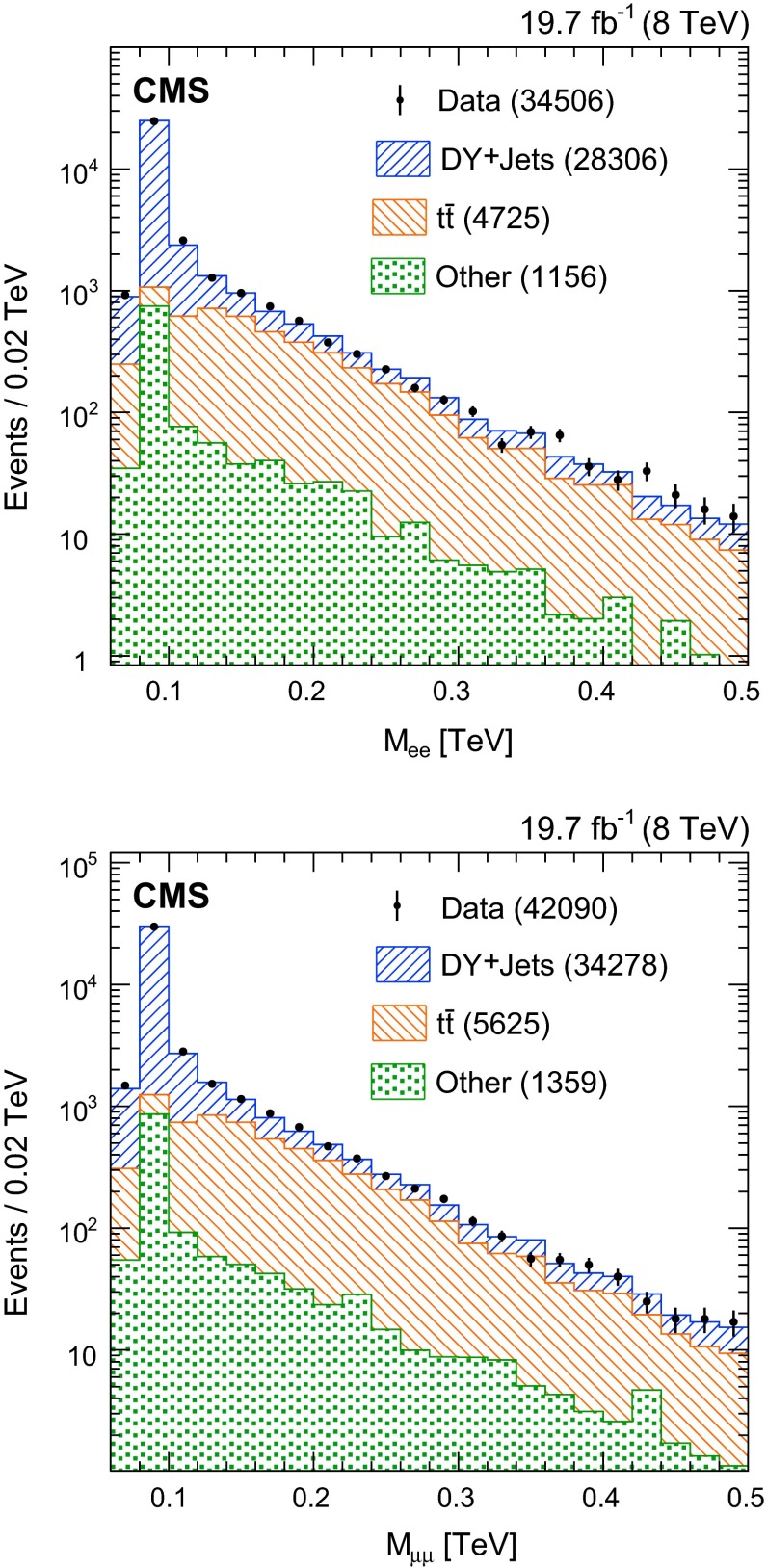
Distribution of the invariant mass $M_{\mathrm {e}\mathrm {e}}$ (*top*) and $M_{\mathrm {\mu }\mathrm {\mu }}$ (*bottom*) for events in data (*points with error bars*) with $p_{\mathrm {T}} > 60\;(40)$ $\,\text {GeV}$ for the leading (subleading) lepton and at least two jets with $p_{\mathrm {T}} > 40$ $\,\text {GeV}$, and for background contributions (*hatched stacked histograms*) from data control samples ($\mathrm {t}\overline{\mathrm {t}}$) and simulation. The numbers of events from each SM process are included in *parentheses in the legend*, where the contributions from diboson and single top quark processes have been collected in the “Other” background category

The DY+jets background contribution is estimated from $\mathrm {Z}/\gamma ^*\rightarrow \ell \ell$ decays reconstructed in simulation and data. The simulated DY+jets background contribution is normalized to data using events in the dilepton mass region $60 < M_{\ell \ell } < 120\,\text {GeV}$ after kinematic requirements are applied on the leptons and jets, which is the first selection stage indicated in Table 1. After removing the small contribution from other SM background processes, the simulated DY+jets distributions are normalized to data using scale factors of $1.000 \pm 0.007$ and $1.027 \pm 0.006$ for the electron and muon channels, respectively, relative to inclusive next-to-next-to-leading-order cross section calculations. The uncertainty in this value reflects the number of events from data with $60 < M_{\ell \ell } < 120\,\text {GeV}$. The shape of the $M_{\ell \ell }$ distribution in data is in agreement with SM expectations for $M_{\ell \ell } > 60$ $\,\text {GeV}$, as shown in Fig. 1.

The diboson and single top quark contributions to the total background are estimated from simulation, based on next-to-leading-order [46] and approximate next-to-next-to-leading-order [47] production cross sections, respectively. The background from $\mathrm {W}$+jets processes, also estimated from simulation, is negligible starting from the earliest selection stage. Finally, the background contribution from multijet processes is estimated using control samples in data and is also found to be negligible at every selection stage.

The observed and expected numbers of events surviving the selections are summarized in Table 1, which explicitly lists the contributions from $\mathrm {t}\overline{\mathrm {t}}$ and DY+jets processes while including all other SM background contributions in a single column. The yields reflect the numbers of background events surviving each selection stage, with normalization factors obtained from simulation and control sample studies or taken directly from simulation. The numbers of events observed at each selection stage agree with SM expectations in both channels.

## The $M_{\mathrm {W}_{\mathrm {R}}}$ distribution and systematic uncertainties

Once all object and event selection criteria are applied, the $M_{\ell \ell j j}$ distributions in data and simulation are used to search for evidence of $\mathrm {W}_{\mathrm {R}}$ boson production, where the expected SM $M_{\ell \ell j j}$ distribution is computed as the sum of the individual background $M_{\ell \ell j j}$ distributions. The $M_{\ell \ell j j}$ distribution is measured in 200$\,\text {GeV}$ wide bins up to 1.8$\,\text {TeV}$, as this bin width is comparable to the mass resolution of the $\mathrm {W}_{\mathrm {R}}$ boson for $M_{\mathrm {W}_{\mathrm {R}}} < 2.5$ $\,\text {TeV}$. Beyond 1.8$\,\text {TeV}$, events are summed in two bins, $1.8 < M_{\ell \ell j j} < 2.2$ $\,\text {TeV}$ and $M_{\ell \ell j j} > 2.2$ $\,\text {TeV}$, to account for the small number of background events in the simulated and data control samples at high mass. The $M_{\ell \ell j j}$ distributions for DY+jets, diboson, and single top quark processes are taken from simulation, with the normalization of each distribution as discussed previously. The $M_{\mathrm {e}\mathrm {\mu }j j}$ distribution from data is used to model the $\mathrm {t}\overline{\mathrm {t}}$ background contribution in the electron and muon channels.

**Fig. 2:**
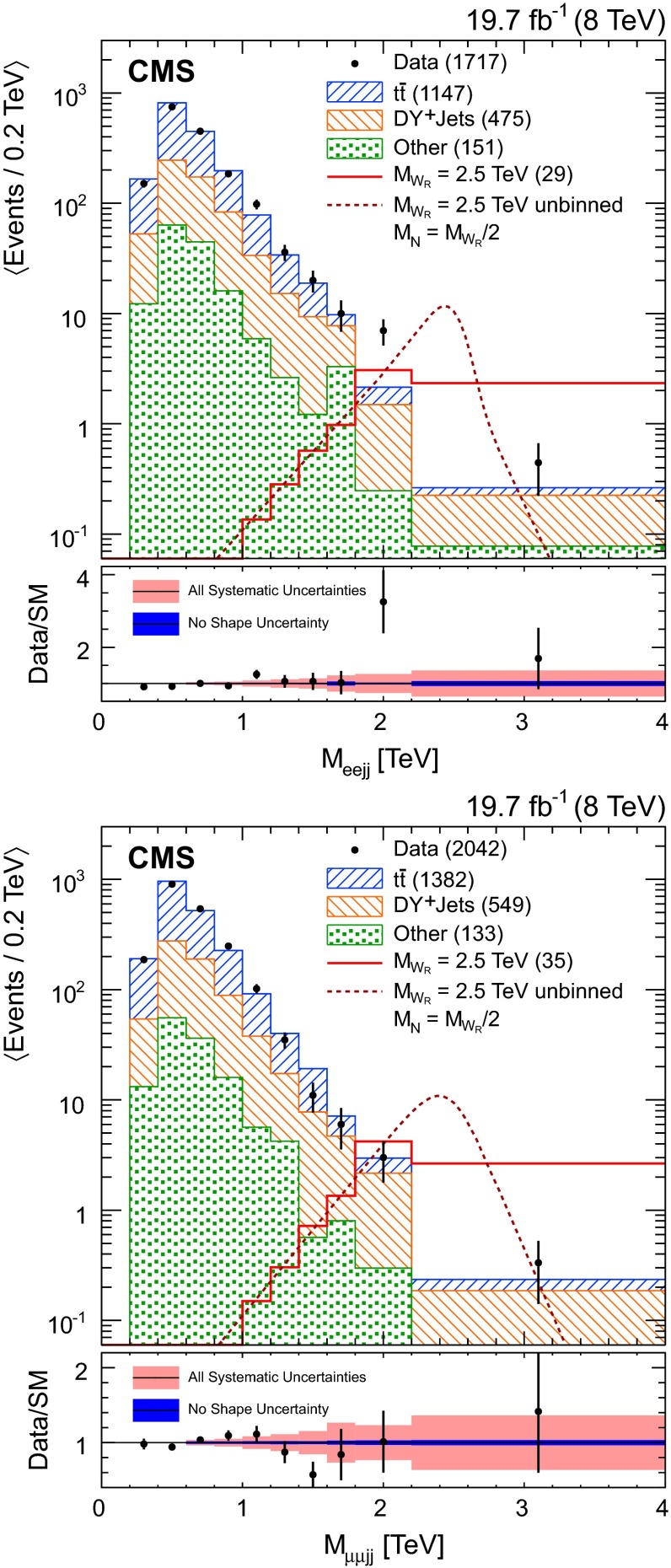
Distribution of the invariant mass $M_{\mathrm {e}\mathrm {e}j j}$ (*top*) and $M_{\mathrm {\mu }\mathrm {\mu }j j}$ (*bottom*) for events in data (*points with error bars*) with $M_{\ell \ell } > 200$ $\,\text {GeV}$ and for background contributions (*hatched stacked histograms*) from data control samples ($\mathrm {t}\overline{\mathrm {t}}$) and simulation. The signal mass point $M_{\mathrm {W}_{\mathrm {R}}}=2.5$ $\,\text {TeV}$, $M_{\mathrm {N}_{\ell }}=1.25$ $\,\text {TeV}$, is included for comparison (*open red histogram*, and also as a *dotted line* for the unbinned signal shape). The numbers of events from each background process (and the expected number of signal events) are included in *parentheses in the legend*, where the contributions from diboson and single top quark processes have been collected in the “Other” background category. The data are compared with SM expectations in the lower portion of the figure. The total background uncertainty (*light red band*) and the background uncertainty after neglecting the uncertainty due to background modeling (*dark blue band*) are included as a function of $M_{\ell \ell j j}$ for $M_{\ell \ell j j} > 600$ $\,\text {GeV}$ (*dashed line*)

In our previous search for $\mathrm {W}_{\mathrm {R}} \rightarrow \mu \mathrm {N}_{\mathrm {\mu }}$ production using 7$\,\text {TeV}$ collision data [13], we modeled the shape of each background $M_{\mathrm {\mu }\mathrm {\mu }j j}$ distribution using an exponential lineshape. For this search, we again find that an exponential function can be used to describe each background $M_{\ell \ell j j}$ distribution below 2$\,\text {TeV}$, but these $M_{\ell \ell j j}$ distributions begin to deviate from the assumed exponential shape at high mass. As a result, in this updated search we use the $M_{\ell \ell j j}$ distributions from each background process directly instead of relying on exponential fits to model the shape of the SM backgrounds.

As the $\mathrm {t}\overline{\mathrm {t}}$ background shape is taken from a control sample of $\mathrm {e}\mu j j$ events in data, we examine the shape of the $\mathrm {t}\overline{\mathrm {t}}$ background $M_{\mathrm {e}\mathrm {\mu }j j}$ distributions in both simulation and data. Based on the method to extract the background shape in our earlier search, we fit each $M_{\mathrm {e}\mathrm {\mu }j j}$ distribution to an exponential lineshape for events surviving all selection criteria for $\mathrm {e}\mu jj$ events. The $\mathrm {t}\overline{\mathrm {t}}$ background distribution is again expected to decrease exponentially as $M_{\ell \ell j j}$ increases, although we allow for deviations at high mass (beyond 2$\,\text {TeV}$) where the DY+jets background is more significant. The simulated $M_{\mathrm {e}\mathrm {\mu }j j}$ distribution agrees with the exponential lineshape for $M_{\mathrm {e}\mathrm {\mu }j j} < 2$ $\,\text {TeV}$, as expected, while we find that the $M_{\mathrm {e}\mathrm {\mu }j j}$ distribution in the data control sample noticeably deviates from fit expectations for $1.0 < M_{\mathrm {e}\mathrm {\mu }j j} < 1.2\,\text {TeV}$. While the fit expects 94 events, only 78 events are found in data in this region. As a result, we correct the $M_{\mathrm {e}\mathrm {\mu }j j}$ distribution from the data control sample to the expected number of events from the exponential fit for $1.0 < M_{\mathrm {e}\mathrm {\mu }j j} < 1.2\,\text {TeV}$, and this correction is reflected in Table 1. The size of the correction is taken as a systematic uncertainty in the shape of the $\mathrm {t}\overline{\mathrm {t}}$ $M_{\ell \ell j j}$ distribution.

The $M_{\ell \ell j j}$ distributions for events satisfying all selection criteria appear in Fig. 2. A comparison of the observed data to SM expectations yields a normalized $\chi ^2$ of 1.4 (0.9) for electron (muon) channel events. We observe an excess in the electron channel in the region $1.8 < M_{\mathrm {e}\mathrm {e}j j} < 2.2\,\text {TeV}$, where 14 events are observed compared to 4 events expected from SM backgrounds. This excess has a local significance of 2.8$\sigma$ estimated using the method discussed in Sect. 7. This excess does not appear to be consistent with $\mathrm {W}_{\mathrm {R}} \rightarrow \mathrm {e}\mathrm {N}_{\mathrm {e}}$ decay. We examined additional distributions for events with $1.8 < M_{\mathrm {e}\mathrm {e}j j} < 2.2\,\text {TeV}$, including the mass distributions $M_{\mathrm {e}jj}$ (for both the leading and subleading electrons), $M_{\mathrm {e}\mathrm {e}}$, and $M_{jj}$, as well as the $p_{\mathrm {T}}$ distributions for each of the final state particles. In this examination, we find no compelling evidence in favor of the signal hypothesis over the assumption of an excess of SM background events in this region. Examining the charge of the electrons used to build $\mathrm {W}_{\mathrm {R}}$ boson candidates in data events with $1.8 < M_{\mathrm {e}\mathrm {e}j j} < 2.2\,\text {TeV}$, we find same-sign electrons in one of the 14 reconstructed events. In this region, the same-sign SM background is expected to be on the order of half an event due to SM diboson processes and charge misidentification in DY+jets events. No same-sign events are observed in the same mass region of the distribution for the muon channel. For comparison, making plausible assumptions for the properties of a signal contributing in this region, one would expect half of the additional events to have electrons with the same sign.

The uncertainties in modeling the shape of the background $M_{\ell \ell j j}$ distributions dominate the background systematic uncertainty, as shown in Fig. 2. The background $M_{\ell \ell j j}$ uncertainty is determined in each mass bin based on the number of events surviving all selection criteria for each background sample. For the two dominant backgrounds, an additional shape uncertainty is included as part of the background shape uncertainty.

The additional $\mathrm {t}\overline{\mathrm {t}}$ shape uncertainty is included for the $1.0 < M_{\ell \ell j j} < 1.2$ $\,\text {TeV}$ mass region based on the previously discussed correction to the $M_{\mathrm {e}\mathrm {\mu }j j}$ distribution for $1.0 < M_{\mathrm {e}\mathrm {\mu }j j} < 1.2$ $\,\text {TeV}$. No additional $\mathrm {t}\overline{\mathrm {t}}$ shape uncertainty is applied at other $M_{\ell \ell j j}$ values as the $M_{\mathrm {e}\mathrm {\mu }j j}$ distributions in both data and simulation agree with the assumed exponential lineshape below 1.8$\,\text {TeV}$, and the statistical uncertainty of the $\mathrm {e}\mu j j$ control sample dominates at high mass. For the DY+jets background, the $M_{\ell \ell j j}$ shape uncertainty is determined using simulated samples from two different MC generators, MadGraph and sherpa. The difference between these two $M_{\ell \ell j j}$ distributions, computed as a function of mass, is taken as an additional systematic uncertainty in the DY+jets shape.

The uncertainty associated with the background normalization is taken as the quadratic sum of the uncertainty in the scale factors determined from the cross-check for $\mathrm {t}\overline{\mathrm {t}}$ background performed on a control region in data, the uncertainty estimated from the difference in the values obtained for DY+jets scale factors in the electron and muon channels, and the combined cross section and luminosity uncertainties for the remaining backgrounds. This overall background normalization uncertainty is small compared to the uncertainties determined for the background shape.

Lepton reconstruction and identification uncertainties, which also contribute to the total signal and background systematic uncertainty, are determined using $\mathrm {Z}\rightarrow \mathrm {e}\mathrm {e}, \mu \mu$ events reconstructed in both data and simulation. Uncertainties in the jet and lepton energy scales and resolutions also contribute to the systematic uncertainty. These uncertainties dominate the signal efficiency uncertainty, resulting in a total systematic uncertainty of up to 10 % for the signal efficiency, depending on the $\mathrm {W}_{\mathrm {R}}$ boson mass assumption. The combination of lepton and jet energy scale, resolution, and efficiency uncertainties is less than 5 % for the background estimates taken from simulation.

The systematic uncertainties related to pileup, uncertainties in the proton PDFs, and initial- or final-state radiation are computed for the simulated background samples and are found to be small when compared to the background shape uncertainty. Additional theoretical uncertainties for the SM background processes are covered by the shape uncertainty. The total uncertainty for signal and background is determined for the final selection stage and presented in Table 1. Figure 2 summarizes the background uncertainty as a function of $M_{\ell \ell j j}$ and displays the dominant background shape uncertainty relative to the total background uncertainty.

## Limits on $\mathrm {W}_{\mathrm {R}}$ boson production

We estimate limits on $\mathrm {W}_{\mathrm {R}}$ boson production using a multibin $\mathrm {CL}_\mathrm {S}$ limit setting technique [48–50]. The $M_{\ell \ell j j}$ distributions obtained from signal MC, each of the SM backgrounds, and the observed data all serve as limit inputs. The systematic uncertainties mentioned previously are included as nuisance parameters in the limit calculations. We estimate the 95 % CL upper limit on the $\mathrm {W}_{\mathrm {R}}$ boson cross section multiplied by the $\mathrm {W}_{\mathrm {R}} \rightarrow \ell \ell jj$ branching fraction as a function of $M_{\mathrm {W}_{\mathrm {R}}}$ and $M_{\mathrm {N}_{\ell }}$. These results [available in tabular form in the supplemental material] can be used for the evaluation of models other than those considered in this paper.

The limits are computed for a set of $\mathrm {W}_{\mathrm {R}}$ boson and $\mathrm {N}_{\ell }$ mass assumptions, where $M_{\mathrm {W}_{\mathrm {R}}}$ starts at 1$\,\text {TeV}$ and increases in 100$\,\text {GeV}$ steps and the $\mathrm {N}_{\ell }$ mass is taken to be half the $\mathrm {W}_{\mathrm {R}}$ boson mass. For these determinations, the $\mathrm {W}_{\mathrm {R}}$ boson signal samples include the full CMS detector simulation.

The procedure to determine the limits on $\mathrm {W}_{\mathrm {R}}$ boson production for a range of $\mathrm {N}_{\ell }$ mass assumptions ($M_{\mathrm {N}_{\ell }} < M_{\mathrm {W}_{\mathrm {R}}}$) proceeds as follows. For a fixed value of $M_{\mathrm {W}_{\mathrm {R}}}$, the limits on $\mathrm {W}_{\mathrm {R}} \rightarrow \ell \mathrm {N}_{\ell } \rightarrow \ell \ell j j$ are determined as a function of $M_{\mathrm {N}_{\ell }}$ (up to $M_{\mathrm {W}_{\mathrm {R}}}$) based on differences in kinematic acceptance, lepton-jet overlap, and $M_{\ell \ell j j}$ shape relative to $M_{\mathrm {N}_{\ell }} = \frac{1}{2} M_{\mathrm {W}_{\mathrm {R}}}$. As mentioned previously, the combined reconstruction and identification efficiency for the $\mathrm {W}_{\mathrm {R}}$ boson and $\mathrm {N}_{\ell }$ decay products varies by $\mathcal {O}(1~\%)$ as a function of $M_{\mathrm {W}_{\mathrm {R}}}$ once acceptance requirements are satisfied. Consequently, for $M_{\mathrm {N}_{\ell }}$ values other than $M_{\mathrm {N}_{\ell }} = \frac{1}{2} M_{\mathrm {W}_{\mathrm {R}}}$, the $\mathrm {W}_{\mathrm {R}}$ boson production cross section limits are computed using information from signal samples that do not include the simulated detector response.

The cross section limit calculation based on the kinematic acceptance is compared with the results for fully simulated samples using a spectrum of $\mathrm {N}_{\ell }$ mass assumptions for $M_{\mathrm {W}_{\mathrm {R}}} =1$, 1.5, 2, and 3$\,\text {TeV}$. The difference between the two methods is at the percent level or smaller for $M_{\mathrm {N}_{\ell }}$ masses greater than 10–20 % of the generated $\mathrm {W}_{\mathrm {R}}$ boson mass. Differences grow to $\mathcal {O}(10)$ % for lighter right-handed neutrinos. The ratio of the products of efficiency and acceptance for the two approaches is computed as a function of $M_{\mathrm {N}_{\ell }} / M_{\mathrm {W}_{\mathrm {R}}}$, and a global fit to this distribution is used to correct the cross section limits determined as a function of $M_{\mathrm {N}_{\ell }}$ for all $M_{\mathrm {W}_{\mathrm {R}}}$ values.

The uncertainty in this correction is computed using the maximum difference in the efficiency times acceptance ratio for the set of simulated samples as a function of $M_{\mathrm {N}_{\ell }} / M_{\mathrm {W}_{\mathrm {R}}}$, unless the statistical uncertainty in the ratio calculation dominates. The impact of this uncertainty on signal acceptance is propagated to the cross section limit calculations. The overall effect on the limits from this uncertainty is negligible for most $M_{\mathrm {N}_{\ell }}$ values, but can degrade the cross section limit by 5–10 % for $\mathrm {N}_{\ell }$ masses below 10 % of $M_{\mathrm {W}_{\mathrm {R}}}$.

Finally, we account for variations in the shape of the $M_{\ell \ell j j}$ distribution. As $M_{\mathrm {N}_{\ell }} \rightarrow 0$, neutrino production via a virtual $\mathrm {W}_{\mathrm {R}}$ boson becomes more significant. As a result, the shape of the signal $M_{\ell \ell j j}$ distribution is expected to vary as a function of both $M_{\mathrm {W}_{\mathrm {R}}}$ and $M_{\mathrm {N}_{\ell }}$. This effect is included in the limit calculations.

The largest uncertainty related to the $\mathrm {W}_{\mathrm {R}} \rightarrow \ell \mathrm {N}_{\ell }$ production estimation arises from the variation in the predicted signal production cross section as a result of the uncertainties in the proton PDFs, where we use the CTEQ6L1 PDF set for signal events. The cross section uncertainty, which is not considered in the limit calculations, ranges from 5 % for $M_{\mathrm {W}_{\mathrm {R}}} = 1$ $\,\text {TeV}$ to 26 % for $M_{\mathrm {W}_{\mathrm {R}}} = 3$ $\,\text {TeV}$ and is computed following the PDF4LHC prescriptions [51, 52] for the CT10 [53], MSTW2008 [54], and NNPDF2.1 [55] PDF sets. The PDF uncertainties in the signal acceptance, which are small compared to the systematic uncertainties for signal events mentioned previously, are included in the limit calculations.

**Fig. 3:**
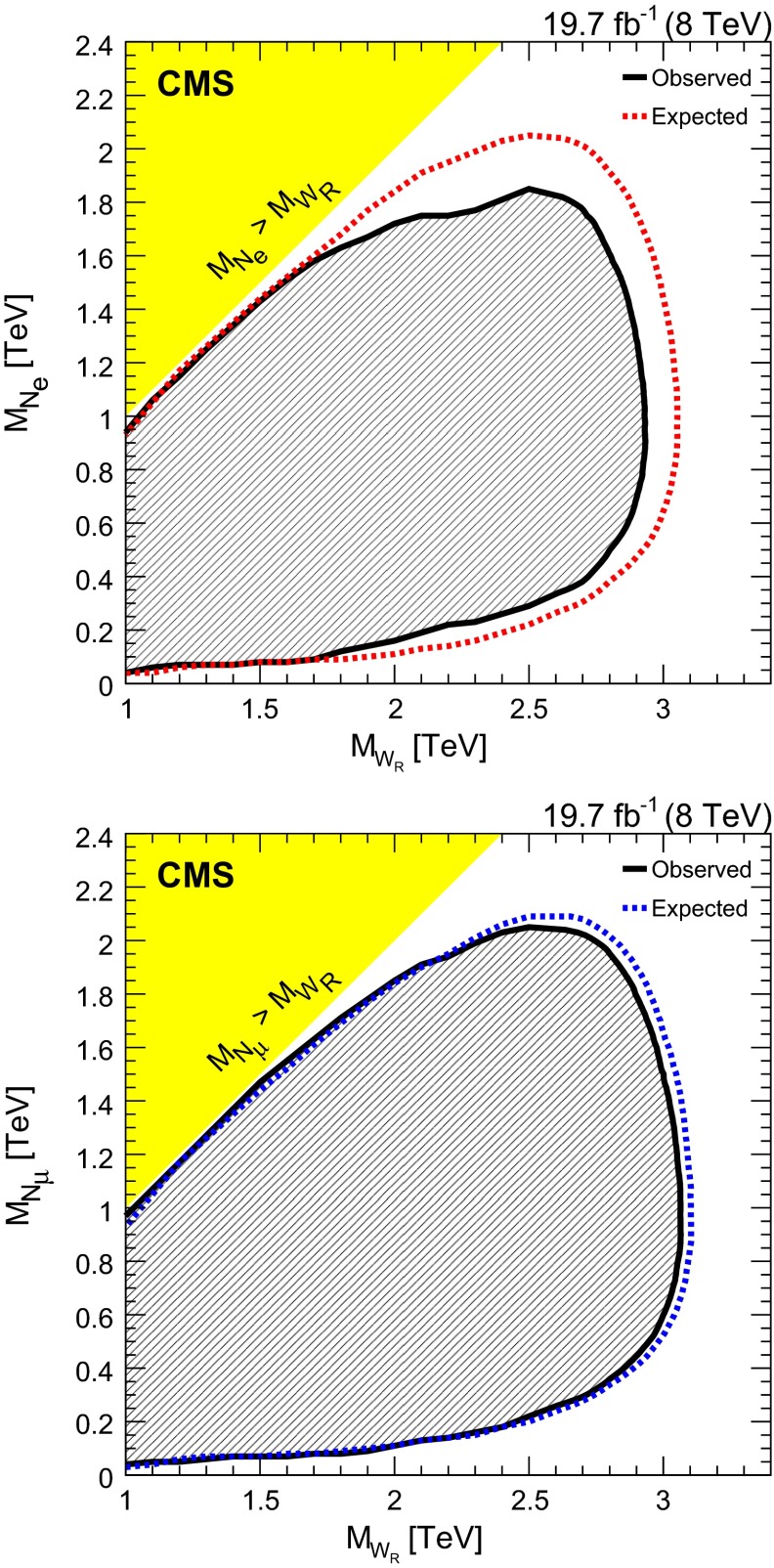
The 95 % CL exclusion region (*hatched*) in the $(M_{\mathrm {W}_{\mathrm {R}}},M_{\mathrm {N}_{\ell }})$ plane, assuming the model described in the text (see Sect. 1), for the electron (*top*) and muon (*bottom*) channels. Neutrino masses greater than $M_{\mathrm {W}_{\mathrm {R}}}$ (*yellow shaded region*) are not considered in this search

**Fig. 4:**
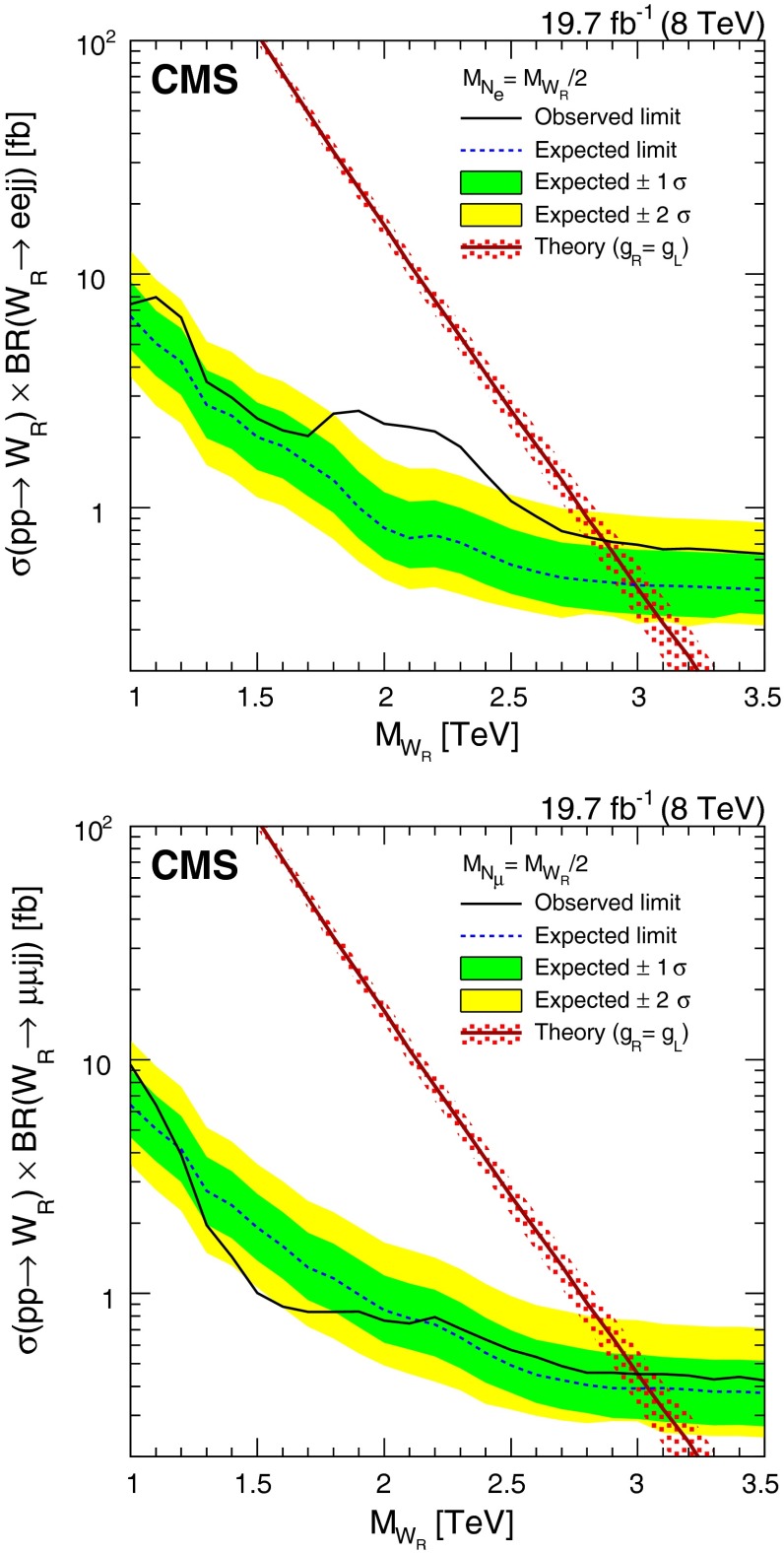
The 95 % CL exclusion for $\mathrm {W}_{\mathrm {R}}$ boson production cross section times branching fraction, computed as a function of $M_{\mathrm {W}_{\mathrm {R}}}$ assuming the right-handed neutrino has half the mass of the $\mathrm {W}_{\mathrm {R}}$ boson, for the electron (*top*) and muon (*bottom*) channels. The signal cross section PDF uncertainties (*red band* surrounding the theoretical $\mathrm {W}_{\mathrm {R}}$-boson production cross section curve) are included for illustration purposes only

For the results presented in Fig. 3, we indicate a range of $\mathrm {N}_{\ell }$ masses that are excluded as a function of $M_{\mathrm {W}_{\mathrm {R}}}$ assuming that only one heavy neutrino flavor (electron or muon) is accessible from 8$\,\text {TeV}$ pp collisions, with the other $\mathrm {N}_{\ell '}$ ($\ell ' = \mathrm {e}, \mu , \tau$, with $\ell ' \ne \ell$) too heavy to be produced. These $(M_{\mathrm {W}_{\mathrm {R}}}, M_{\mathrm {N}_{\ell }})$ limits are obtained by comparing the observed and expected cross section upper limits with the expected cross section for each mass point. The limits extend to roughly $M_{\mathrm {W}_{\mathrm {R}}} = 3.0$ $\,\text {TeV}$ in each channel and exclude a wide range of heavy neutrino masses for $\mathrm {W}_{\mathrm {R}}$ boson mass assumptions below this maximal value. The inclusion of the results from the previous iteration of this analysis [13], which searched for $\mathrm {W}_{\mathrm {R}}$ boson production in the $\mu \mu jj$ final state using 7$\,\text {TeV}$ data, does not significantly affect the limit results. The excess in the electron channel at approximately 2$\,\text {TeV}$ has a local significance of 2.8$\sigma$ for a $\mathrm {W}_{\mathrm {R}}$ boson candidate with a mass of 2.1$\,\text {TeV}$. Assuming contributions from SM backgrounds only, the p value for the local excess in the $M_{\mathrm {e}\mathrm {e}j j}$ distribution is 0.0050. We also present limits as a function of $\mathrm {W}_{\mathrm {R}}$ boson mass for a right-handed neutrino with $M_{\mathrm {N}_{\ell }} = \frac{1}{2} M_{\mathrm {W}_{\mathrm {R}}}$ in Fig. 4. For the electron (muon) channel, we exclude $\mathrm {W}_{\mathrm {R}}$ bosons with $M_{\mathrm {W}_{\mathrm {R}}} < 2.87\;(3.00)$ $\,\text {TeV}$, with an expected exclusion of 2.99 (3.04)$\,\text {TeV}$.

**Fig. 5:**
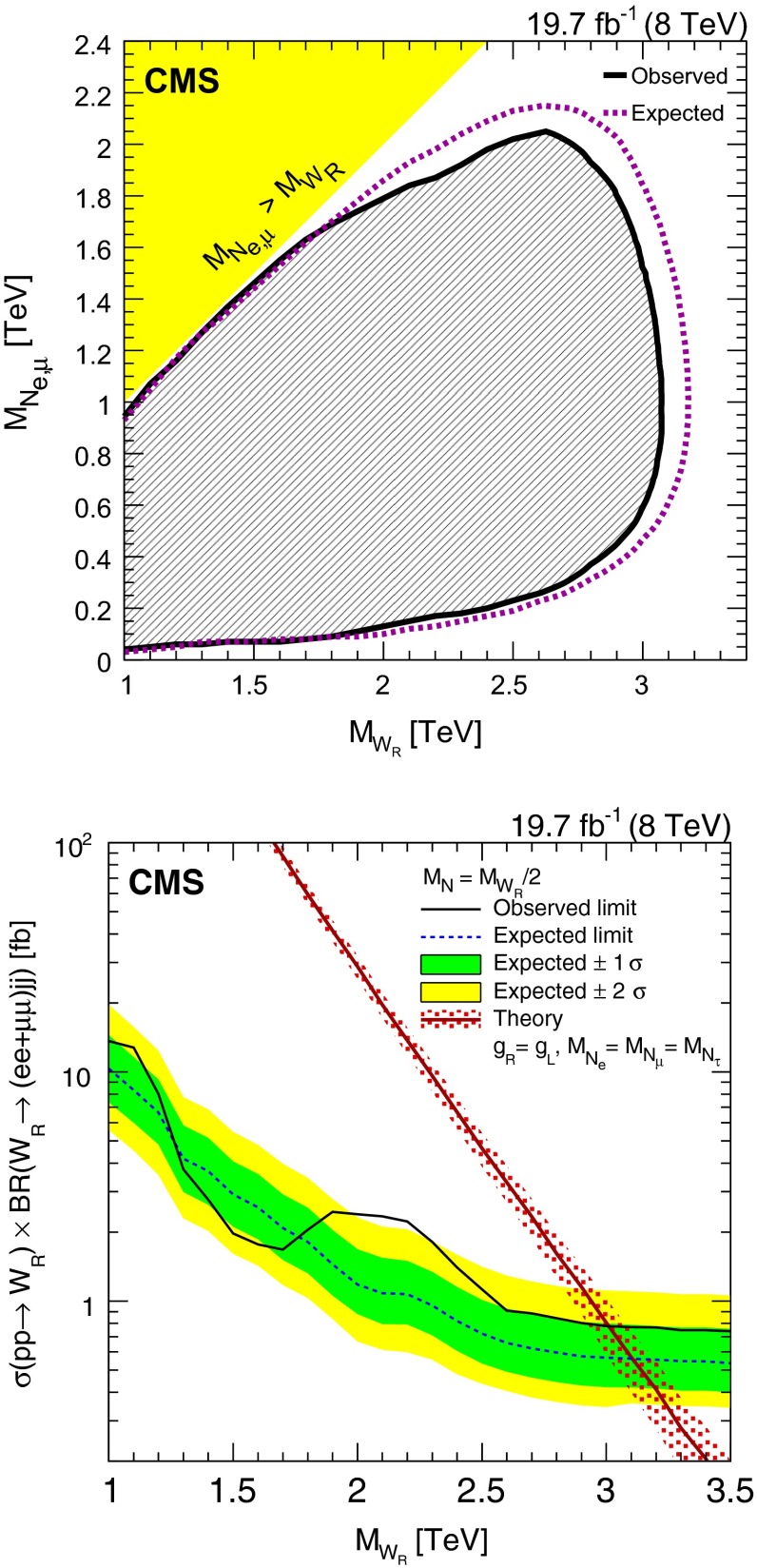
The 95 % CL exclusion region in the $(M_{\mathrm {W}_{\mathrm {R}}},M_{\mathrm {N}_{\ell }})$ plane (*top*), and as a function of $\mathrm {W}_{\mathrm {R}}$ boson mass with $M_N = \frac{1}{2} M_{\mathrm {W}_{\mathrm {R}}}$ (*bottom*) obtained combining the electron and muon channels. The signal cross section PDF uncertainties (*red band* surrounding the theoretical $\mathrm {W}_{\mathrm {R}}$-boson production cross section curve) are included for illustration purposes only. Neutrino masses greater than $M_{\mathrm {W}_{\mathrm {R}}}$ (*yellow shaded region in the top figure*) are not considered in this search

We additionally consider the case where all $\mathrm {N}_{\ell }$ masses are degenerate and can be produced via $\mathrm {W}_{\mathrm {R}}$ boson production and decay in 8$\,\text {TeV}$ pp collisions. In this case, the electron and muon results can be combined as shown in Fig. 5. The $(M_{\mathrm {W}_{\mathrm {R}}},M_{\mathrm {N}_{\ell }})$ exclusion for the combination extends slightly further than the single-channel exclusion limits, with an observed (expected) exclusion for the combined channel of $M_{\mathrm {W}_{\mathrm {R}}} < 3.01~(3.10)$ $\,\text {TeV}$ for $M_{\mathrm {N}_{\ell }} = \frac{1}{2} M_{\mathrm {W}_{\mathrm {R}}}$.

## Summary

A search for right-handed bosons ($\mathrm {W}_{\mathrm {R}}$) and heavy right-handed neutrinos ($\mathrm {N}_{\ell }$) in the left-right symmetric extension of the standard model has been presented. The data sample is in agreement with expectations from standard model processes in the $\mu \mu j j$ final state. An excess is observed in the electron channel with a local significance of 2.8$\sigma$ at $M_{\mathrm {e}\mathrm {e}j j} \approx 2.1$ $\,\text {TeV}$. The excess does not appear to be consistent with expectations from left-right symmetric theory. Considering $\mathrm {W}_{\mathrm {R}} \rightarrow \mathrm {e}\mathrm {N}_{\mathrm {e}}$ and $\mathrm {W}_{\mathrm {R}} \rightarrow \mu \mathrm {N}_{\mathrm {\mu }}$ searches separately, regions in the $(M_{\mathrm {W}_{\mathrm {R}}},M_{\mathrm {N}_{\ell }})$ mass space are excluded at 95 % confidence level that extend up to $M_{\mathrm {W}_{\mathrm {R}}} < 3.0$ $\,\text {TeV}$ for both channels. Assuming $\mathrm {W}_{\mathrm {R}} \rightarrow \ell \mathrm {N}_{\ell }$ with degenerate $\mathrm {N}_{\ell }$ mass for $\ell = \mathrm {e}, \mu$, $\mathrm {W}_{\mathrm {R}}$ boson production is excluded at 95 % confidence level up to $M_{\mathrm {W}_{\mathrm {R}}} < 3.0$ $\,\text {TeV}$. This search has significantly extended the exclusion region in the two-dimensional $(M_{\mathrm {W}_{\mathrm {R}}},M_{\mathrm {N}_{\ell }})$ mass plane compared to previous searches, and for the first time this search has excluded $M_{\mathrm {W}_{\mathrm {R}}}$ values beyond the theoretical lower mass limit of $M_{\mathrm {W}_{\mathrm {R}}} \gtrsim 2.5$ $\,\text {TeV}$.

## Electronic supplementary material

Below is the link to the electronic supplementary material.

Supplementary material 1 (pdf 220 KB)
