# Supplementary material for: Search for heavy neutrinos and W bosons with right-handed couplings in proton–proton collisions at √s = 8 TeV
Source: Eur Phys J C Part Fields. 2014 Nov 26;74(11):3149. doi: 10.1140/epjc/s10052-014-3149-z (PMC4371073; doi:10.1140/epjc/s10052-014-3149-z)
Supplement: Supplementary file 1 — Supplementary material 1 (pdf 220 KB) [file 10052_2014_3149_MOESM1_ESM.pdf]

e-mail: cms-publication-committee-chair@cern.ch

Key words. CMS,physics

Search for heavy neutrinos and W_R bosons with right-handed couplings in a left-right symmetric model in pp collisions at $\sqrt{s}$ = 8 TeV

—Supplemental Material—

The CMS Collaboration

CERN

October 1, 2014

Table A.1. The 95% CL observed (Obs.) and expected (Exp.) exclusion limits (in fb) on the W_R production cross section times branching fraction for $W_R \to eejj$ as a function of W_R and N_e mass (in GeV) for $1000 \leq M_{W_R} \leq 1600$ GeV. The signal acceptance (Acc.) is also included for each (M_{W_R}, M_{N_e}) entry.

M_{W_R}	M_{N_e}	Obs.	Exp.	Acc.	M_{W_R}	M_{N_e}	Obs.	Exp.	Acc.
1000	100	65.7	58.5	0.073 ± 0.110	1400	100	60.1	50.2	0.043 ± 0.164
1000	200	14.6	13.0	0.298 ± 0.031	1400	200	9.07	7.57	0.241 ± 0.061
1000	300	9.77	8.69	0.447 ± 0.014	1400	300	4.91	4.09	0.428 ± 0.027
1000	400	8.17	7.27	0.549 ± 0.011	1400	400	3.76	3.13	0.549 ± 0.015
1000	500	7.44	6.63	0.596 ± 0.009	1400	500	3.29	2.75	0.630 ± 0.011
1000	600	7.14	6.35	0.620 ± 0.009	1400	600	3.09	2.58	0.688 ± 0.010
1000	700	7.16	6.38	0.617 ± 0.009	1400	700	2.97	2.48	0.711 ± 0.011
1000	800	7.71	6.86	0.573 ± 0.009	1400	800	2.89	2.41	0.729 ± 0.009
1000	900	10.2	9.05	0.435 ± 0.010	1400	900	2.89	2.41	0.729 ± 0.009
1100	100	97.0	61.6	0.062 ± 0.124	1400	1000	2.90	2.42	0.725 ± 0.009
1100	200	17.6	11.2	0.290 ± 0.038	1400	1100	2.96	2.47	0.712 ± 0.009
1100	300	10.3	6.56	0.441 ± 0.016	1400	1200	3.17	2.64	0.664 ± 0.009
1100	400	8.61	5.47	0.548 ± 0.011	1400	1300	3.77	3.15	0.558 ± 0.010
1100	500	8.05	5.11	0.609 ± 0.010	1500	100	59.4	49.4	0.038 ± 0.176
1100	600	7.69	4.89	0.655 ± 0.010	1500	200	8.23	6.86	0.221 ± 0.070
1100	700	7.59	4.82	0.663 ± 0.009	1500	300	3.97	3.31	0.411 ± 0.031
1100	800	7.82	4.96	0.644 ± 0.010	1500	400	3.00	2.50	0.545 ± 0.017
1100	900	8.30	5.27	0.606 ± 0.009	1500	500	2.64	2.20	0.625 ± 0.012
1100	1000	10.6	6.75	0.473 ± 0.010	1500	600	2.49	2.07	0.686 ± 0.010
1200	100	91.5	59.2	0.052 ± 0.139	1500	700	2.41	2.01	0.716 ± 0.010
1200	200	16.2	10.5	0.275 ± 0.045	1500	800	2.37	1.98	0.739 ± 0.011
1200	300	9.76	6.32	0.442 ± 0.019	1500	900	2.35	1.95	0.746 ± 0.009
1200	400	7.81	5.05	0.556 ± 0.012	1500	1000	2.32	1.93	0.755 ± 0.009
1200	500	6.98	4.52	0.627 ± 0.010	1500	1100	2.37	1.97	0.739 ± 0.009
1200	600	6.53	4.23	0.671 ± 0.009	1500	1200	2.46	2.05	0.711 ± 0.009
1200	700	6.39	4.14	0.684 ± 0.009	1500	1300	2.59	2.16	0.675 ± 0.009
1200	800	6.36	4.12	0.687 ± 0.009	1500	1400	3.06	2.55	0.573 ± 0.009
1200	900	6.48	4.20	0.674 ± 0.009	1600	100	58.8	50.4	0.033 ± 0.187
1200	1000	6.98	4.52	0.626 ± 0.009	1600	200	8.08	6.93	0.212 ± 0.078
1200	1100	8.71	5.64	0.502 ± 0.009	1600	300	3.91	3.35	0.402 ± 0.036
1300	100	61.7	49.2	0.047 ± 0.152	1600	400	2.89	2.48	0.536 ± 0.019
1300	200	9.48	7.55	0.254 ± 0.054	1600	500	2.49	2.13	0.627 ± 0.017
1300	300	4.99	3.97	0.446 ± 0.023	1600	600	2.27	1.95	0.680 ± 0.010
1300	400	3.95	3.14	0.558 ± 0.013	1600	700	2.21	1.89	0.723 ± 0.009
1300	500	3.60	2.86	0.635 ± 0.010	1600	800	2.14	1.84	0.747 ± 0.010
1300	600	3.46	2.76	0.684 ± 0.010	1600	900	2.10	1.80	0.760 ± 0.009
1300	700	3.41	2.72	0.699 ± 0.009	1600	1000	2.09	1.79	0.763 ± 0.009
1300	800	3.32	2.65	0.716 ± 0.009	1600	1100	2.10	1.80	0.761 ± 0.009
1300	900	3.35	2.67	0.710 ± 0.009	1600	1200	2.13	1.83	0.749 ± 0.009
1300	1000	3.41	2.72	0.698 ± 0.009	1600	1300	2.18	1.87	0.732 ± 0.009
1300	1100	3.66	2.92	0.650 ± 0.009	1600	1400	2.30	1.97	0.695 ± 0.012
1300	1200	4.43	3.53	0.536 ± 0.009	1600	1500	2.70	2.31	0.592 ± 0.009

Table A.2. The 95% CL observed (Obs.) and expected (Exp.) exclusion limits (in fb) on the W_R production cross section times branching fraction for $W_R \to eejj$ as a function of W_R and N_e mass (in GeV) for $1700 \leq M_{W_R} \leq 2000$ GeV. The signal acceptance (Acc.) is also included for each (M_{W_R}, M_{N_e}) entry.

M_{W_R}	M_{N_e}	Obs.	Exp.	Acc.	M_{W_R}	M_{N_e}	Obs.	Exp.	Acc.
1700	100	76.5	58.3	0.032 ± 0.197	1900	100	131	50.7	0.025 ± 0.216
1700	200	9.12	6.95	0.195 ± 0.086	1900	200	14.3	5.51	0.161 ± 0.103
1700	300	3.87	2.95	0.383 ± 0.040	1900	300	5.83	2.25	0.348 ± 0.055
1700	400	2.71	2.07	0.526 ± 0.022	1900	400	3.91	1.51	0.502 ± 0.028
1700	500	2.30	1.75	0.616 ± 0.015	1900	500	3.23	1.25	0.605 ± 0.025
1700	600	2.14	1.63	0.676 ± 0.011	1900	600	2.89	1.12	0.674 ± 0.013
1700	700	2.09	1.59	0.719 ± 0.010	1900	700	2.74	1.06	0.715 ± 0.011
1700	800	2.02	1.54	0.751 ± 0.011	1900	800	2.67	1.03	0.747 ± 0.010
1700	900	2.00	1.52	0.767 ± 0.009	1900	900	2.61	1.01	0.769 ± 0.009
1700	1000	1.98	1.51	0.774 ± 0.009	1900	1000	2.56	0.987	0.782 ± 0.011
1700	1100	1.97	1.50	0.776 ± 0.009	1900	1100	2.55	0.983	0.784 ± 0.011
1700	1200	1.98	1.51	0.770 ± 0.009	1900	1200	2.54	0.980	0.787 ± 0.009
1700	1300	2.01	1.53	0.762 ± 0.011	1900	1300	2.53	0.978	0.788 ± 0.009
1700	1400	2.07	1.58	0.739 ± 0.009	1900	1400	2.54	0.979	0.787 ± 0.009
1700	1500	2.18	1.66	0.702 ± 0.009	1900	1500	2.60	1.00	0.770 ± 0.009
1700	1600	2.51	1.91	0.609 ± 0.010	1900	1600	2.64	1.02	0.756 ± 0.009
1800	100	105	54.5	0.027 ± 0.207	1900	1700	2.79	1.08	0.715 ± 0.009
1800	200	13.0	6.74	0.178 ± 0.095	1900	1800	3.19	1.23	0.626 ± 0.010
1800	300	5.61	2.91	0.363 ± 0.045	2000	100	139	49.5	0.023 ± 0.225
1800	400	3.86	2.00	0.514 ± 0.026	2000	200	15.7	5.62	0.150 ± 0.110
1800	500	3.14	1.63	0.605 ± 0.016	2000	300	6.08	2.17	0.328 ± 0.056
1800	600	2.82	1.46	0.671 ± 0.012	2000	400	3.87	1.38	0.483 ± 0.031
1800	700	2.66	1.38	0.720 ± 0.010	2000	500	3.03	1.08	0.593 ± 0.020
1800	800	2.56	1.33	0.748 ± 0.009	2000	600	2.66	0.952	0.665 ± 0.014
1800	900	2.53	1.31	0.765 ± 0.009	2000	700	2.50	0.892	0.709 ± 0.011
1800	1000	2.49	1.29	0.776 ± 0.009	2000	800	2.38	0.850	0.743 ± 0.010
1800	1100	2.46	1.28	0.784 ± 0.015	2000	900	2.33	0.832	0.766 ± 0.009
1800	1200	2.46	1.28	0.784 ± 0.009	2000	1000	2.29	0.820	0.784 ± 0.009
1800	1300	2.47	1.28	0.783 ± 0.009	2000	1100	2.27	0.811	0.791 ± 0.009
1800	1400	2.51	1.30	0.769 ± 0.009	2000	1200	2.24	0.800	0.802 ± 0.009
1800	1500	2.58	1.34	0.749 ± 0.010	2000	1300	2.23	0.798	0.803 ± 0.009
1800	1600	2.73	1.41	0.709 ± 0.015	2000	1400	2.25	0.805	0.796 ± 0.009
1800	1700	3.15	1.63	0.613 ± 0.009	2000	1500	2.27	0.812	0.789 ± 0.009
					2000	1600	2.30	0.822	0.779 ± 0.009
					2000	1700	2.35	0.838	0.764 ± 0.009
					2000	1800	2.49	0.889	0.720 ± 0.011
					2000	1900	2.84	1.02	0.631 ± 0.010

Table A.3. The 95% CL observed (Obs.) and expected (Exp.) exclusion limits (in fb) on the W_R production cross section times branching fraction for $W_R \rightarrow eejj$ as a function of W_R and N_e mass (in GeV) for $2100 \leq M_{W_R} \leq 2300$ GeV. The signal acceptance (Acc.) is also included for each (M_{W_R}, M_{N_e}) entry.

M_{W_R}	M_{N_e}	Obs.	Exp.	Acc.	M_{W_R}	M_{N_e}	Obs.	Exp.	Acc.
2100	100	173	57.8	0.024 ± 0.233	2300	100	158	61.5	0.022 ± 0.251
2100	200	18.1	6.07	0.142 ± 0.117	2300	200	15.5	6.00	0.134 ± 0.133
2100	300	6.94	2.32	0.315 ± 0.061	2300	300	5.76	2.24	0.297 ± 0.072
2100	400	4.26	1.42	0.469 ± 0.034	2300	400	3.42	1.33	0.438 ± 0.042
2100	500	3.25	1.09	0.586 ± 0.021	2300	500	2.62	1.02	0.554 ± 0.026
2100	600	2.77	0.927	0.657 ± 0.015	2300	600	2.26	0.879	0.651 ± 0.018
2100	700	2.52	0.843	0.710 ± 0.012	2300	700	2.06	0.798	0.691 ± 0.014
2100	800	2.38	0.796	0.745 ± 0.010	2300	800	1.96	0.759	0.736 ± 0.011
2100	900	2.29	0.767	0.759 ± 0.010	2300	900	1.90	0.738	0.760 ± 0.010
2100	1000	2.24	0.750	0.785 ± 0.009	2300	1000	1.85	0.716	0.779 ± 0.010
2100	1100	2.21	0.738	0.791 ± 0.009	2300	1100	1.82	0.704	0.793 ± 0.010
2100	1200	2.19	0.732	0.797 ± 0.011	2300	1200	1.81	0.703	0.801 ± 0.012
2100	1300	2.16	0.724	0.806 ± 0.011	2300	1300	1.79	0.695	0.809 ± 0.032
2100	1400	2.17	0.726	0.802 ± 0.011	2300	1400	1.78	0.691	0.813 ± 0.009
2100	1500	2.18	0.728	0.800 ± 0.009	2300	1500	1.78	0.689	0.815 ± 0.009
2100	1600	2.20	0.734	0.793 ± 0.009	2300	1600	1.78	0.692	0.812 ± 0.009
2100	1700	2.24	0.748	0.779 ± 0.011	2300	1700	1.80	0.697	0.806 ± 0.010
2100	1800	2.29	0.767	0.759 ± 0.010	2300	1800	1.81	0.701	0.801 ± 0.030
2100	1900	2.39	0.801	0.727 ± 0.009	2300	1900	1.83	0.711	0.790 ± 0.010
2100	2000	2.71	0.906	0.643 ± 0.010	2300	2000	1.89	0.734	0.765 ± 0.009
2200	100	161	57.9	0.024 ± 0.242	2300	2100	1.98	0.767	0.732 ± 0.013
2200	200	17.3	6.23	0.135 ± 0.125	2300	2200	2.21	0.856	0.656 ± 0.011
2200	300	6.67	2.40	0.300 ± 0.067					
2200	400	4.17	1.50	0.459 ± 0.038					
2200	500	3.23	1.16	0.568 ± 0.023					
2200	600	2.75	0.990	0.647 ± 0.016					
2200	700	2.48	0.894	0.704 ± 0.013					
2200	800	2.33	0.838	0.735 ± 0.012					
2200	900	2.24	0.804	0.765 ± 0.011					
2200	1000	2.17	0.780	0.784 ± 0.009					
2200	1100	2.12	0.763	0.793 ± 0.010					
2200	1200	2.10	0.757	0.799 ± 0.009					
2200	1300	2.07	0.744	0.812 ± 0.009					
2200	1400	2.10	0.754	0.801 ± 0.010					
2200	1500	2.08	0.747	0.808 ± 0.010					
2200	1600	2.08	0.749	0.806 ± 0.009					
2200	1700	2.10	0.756	0.798 ± 0.009					
2200	1800	2.13	0.768	0.786 ± 0.009					
2200	1900	2.19	0.786	0.768 ± 0.009					
2200	2000	2.30	0.826	0.730 ± 0.010					
2200	2100	2.57	0.925	0.653 ± 0.030					

Table A.4. The 95% CL observed (Obs.) and expected (Exp.) exclusion limits (in fb) on the W_R production cross section times branching fraction for $W_R \to eejj$ as a function of W_R and N_e mass (in GeV) for $2400 \leq M_{W_R} \leq 2500$ GeV. The signal acceptance (Acc.) is also included for each (M_{W_R}, M_{N_e}) entry.

M_{W_R}	M_{N_e}	Obs.	Exp.	Acc.	M_{W_R}	M_{N_e}	Obs.	Exp.	Acc.
2400	100	160	73.2	0.025 ± 0.262	2500	100	157	83.8	0.027 ± 0.270
2400	200	14.3	6.57	0.127 ± 0.139	2500	200	13.6	7.27	0.125 ± 0.149
2400	300	5.09	2.33	0.278 ± 0.078	2500	300	4.63	2.47	0.265 ± 0.085
2400	400	2.93	1.34	0.429 ± 0.060	2500	400	2.60	1.39	0.414 ± 0.059
2400	500	2.16	0.990	0.548 ± 0.028	2500	500	1.82	0.972	0.539 ± 0.032
2400	600	1.79	0.821	0.634 ± 0.019	2500	600	1.50	0.800	0.622 ± 0.021
2400	700	1.63	0.745	0.691 ± 0.015	2500	700	1.32	0.705	0.683 ± 0.015
2400	800	1.52	0.696	0.732 ± 0.012	2500	800	1.22	0.651	0.729 ± 0.013
2400	900	1.54	0.706	0.757 ± 0.011	2500	900	1.15	0.613	0.750 ± 0.011
2400	1000	1.49	0.683	0.778 ± 0.010	2500	1000	1.12	0.598	0.776 ± 0.010
2400	1100	1.43	0.654	0.794 ± 0.009	2500	1100	1.09	0.581	0.790 ± 0.010
2400	1200	1.39	0.636	0.807 ± 0.010	2500	1200	1.08	0.574	0.803 ± 0.010
2400	1300	1.38	0.630	0.813 ± 0.012	2500	1300	1.07	0.569	0.809 ± 0.010
2400	1400	1.38	0.631	0.811 ± 0.009	2500	1400	1.06	0.565	0.814 ± 0.009
2400	1500	1.37	0.629	0.814 ± 0.009	2500	1500	1.05	0.560	0.820 ± 0.011
2400	1600	1.37	0.626	0.817 ± 0.009	2500	1600	1.04	0.555	0.827 ± 0.009
2400	1700	1.37	0.626	0.817 ± 0.011	2500	1700	1.06	0.563	0.815 ± 0.012
2400	1800	1.38	0.634	0.807 ± 0.011	2500	1800	1.06	0.565	0.812 ± 0.009
2400	1900	1.39	0.638	0.801 ± 0.009	2500	1900	1.06	0.563	0.814 ± 0.019
2400	2000	1.41	0.646	0.792 ± 0.009	2500	2000	1.07	0.569	0.806 ± 0.009
2400	2100	1.44	0.660	0.775 ± 0.009	2500	2100	1.08	0.576	0.797 ± 0.009
2400	2200	1.51	0.693	0.738 ± 0.011	2500	2200	1.12	0.594	0.772 ± 0.012
2400	2300	1.69	0.772	0.662 ± 0.014	2500	2300	1.16	0.620	0.740 ± 0.011
					2500	2400	1.30	0.691	0.664 ± 0.011

Table A.5. The 95% CL observed (Obs.) and expected (Exp.) exclusion limits (in fb) on the W_R production cross section times branching fraction for $W_R \to eejj$ as a function of W_R and N_e mass (in GeV) for $2600 \leq M_{W_R} \leq 2700$ GeV. The signal acceptance (Acc.) is also included for each (M_{W_R}, M_{N_e}) entry.

M_{W_R}	M_{N_e}	Obs.	Exp.	Acc.	M_{W_R}	M_{N_e}	Obs.	Exp.	Acc.
2600	100	148	86.1	0.023 ± 0.276	2700	100	131	83.0	0.026 ± 0.288
2600	200	13.9	8.07	0.122 ± 0.157	2700	200	13.2	8.35	0.127 ± 0.165
2600	300	4.60	2.67	0.256 ± 0.090	2700	300	4.36	2.76	0.257 ± 0.098
2600	400	2.46	1.43	0.404 ± 0.054	2700	400	2.29	1.45	0.394 ± 0.059
2600	500	1.71	0.993	0.521 ± 0.034	2700	500	1.56	0.986	0.513 ± 0.037
2600	600	1.37	0.794	0.616 ± 0.024	2700	600	1.22	0.770	0.613 ± 0.025
2600	700	1.19	0.689	0.682 ± 0.018	2700	700	1.07	0.678	0.674 ± 0.018
2600	800	1.09	0.632	0.722 ± 0.013	2700	800	0.993	0.628	0.719 ± 0.015
2600	900	1.03	0.596	0.744 ± 0.013	2700	900	0.918	0.580	0.748 ± 0.012
2600	1000	0.977	0.568	0.771 ± 0.010	2700	1000	0.851	0.538	0.774 ± 0.012
2600	1100	0.941	0.547	0.783 ± 0.010	2700	1100	0.824	0.521	0.785 ± 0.011
2600	1200	0.947	0.550	0.803 ± 0.010	2700	1200	0.776	0.491	0.799 ± 0.010
2600	1300	0.918	0.534	0.807 ± 0.010	2700	1300	0.781	0.494	0.810 ± 0.009
2600	1400	0.912	0.530	0.812 ± 0.010	2700	1400	0.794	0.502	0.814 ± 0.010
2600	1500	0.907	0.527	0.816 ± 0.012	2700	1500	0.787	0.498	0.820 ± 0.014
2600	1600	0.897	0.521	0.825 ± 0.011	2700	1600	0.781	0.494	0.826 ± 0.014
2600	1700	0.896	0.521	0.825 ± 0.019	2700	1700	0.784	0.496	0.823 ± 0.034
2600	1800	0.901	0.523	0.821 ± 0.010	2700	1800	0.781	0.494	0.826 ± 0.016
2600	1900	0.903	0.525	0.819 ± 0.011	2700	1900	0.783	0.495	0.823 ± 0.010
2600	2000	0.908	0.528	0.814 ± 0.010	2700	2000	0.783	0.495	0.823 ± 0.010
2600	2100	0.914	0.531	0.808 ± 0.011	2700	2100	0.787	0.498	0.819 ± 0.016
2600	2200	0.927	0.539	0.797 ± 0.017	2700	2200	0.801	0.506	0.805 ± 0.011
2600	2300	0.950	0.552	0.778 ± 0.009	2700	2300	0.811	0.513	0.795 ± 0.013
2600	2400	1.00	0.581	0.739 ± 0.010	2700	2400	0.830	0.525	0.776 ± 0.009
2600	2500	1.11	0.645	0.666 ± 0.013	2700	2500	0.872	0.551	0.739 ± 0.010
					2700	2600	0.954	0.603	0.676 ± 0.012

Table A.6. The 95% CL observed (Obs.) and expected (Exp.) exclusion limits (in fb) on the W_R production cross section times branching fraction for $W_R \to eejj$ as a function of W_R and N_e mass (in GeV) for $2800 \leq M_{W_R} \leq 2900$ GeV. The signal acceptance (Acc.) is also included for each (M_{W_R}, M_{N_e}) entry.

M_{W_R}	M_{N_e}	Obs.	Exp.	Acc.	M_{W_R}	M_{N_e}	Obs.	Exp.	Acc.
2800	100	138	90.4	0.028 ± 0.300	2900	100	136	91.3	0.030 ± 0.307
2800	200	15.2	9.93	0.130 ± 0.175	2900	200	16.8	11.2	0.130 ± 0.187
2800	300	5.01	3.27	0.255 ± 0.108	2900	300	5.42	3.62	0.247 ± 0.115
2800	400	2.56	1.67	0.385 ± 0.064	2900	400	2.71	1.81	0.391 ± 0.071
2800	500	1.69	1.10	0.507 ± 0.041	2900	500	1.74	1.16	0.498 ± 0.045
2800	600	1.30	0.851	0.601 ± 0.027	2900	600	1.32	0.880	0.595 ± 0.029
2800	700	1.09	0.713	0.666 ± 0.020	2900	700	1.10	0.735	0.658 ± 0.022
2800	800	1.00	0.655	0.708 ± 0.015	2900	800	0.966	0.646	0.701 ± 0.016
2800	900	0.900	0.587	0.739 ± 0.012	2900	900	0.882	0.590	0.737 ± 0.014
2800	1000	0.851	0.556	0.765 ± 0.012	2900	1000	0.830	0.555	0.758 ± 0.013
2800	1100	0.814	0.531	0.779 ± 0.018	2900	1100	0.790	0.528	0.778 ± 0.027
2800	1200	0.785	0.512	0.799 ± 0.013	2900	1200	0.761	0.509	0.795 ± 0.013
2800	1300	0.762	0.497	0.808 ± 0.010	2900	1300	0.737	0.493	0.810 ± 0.011
2800	1400	0.751	0.490	0.816 ± 0.010	2900	1400	0.721	0.482	0.815 ± 0.010
2800	1500	0.743	0.485	0.823 ± 0.010	2900	1500	0.714	0.478	0.817 ± 0.011
2800	1600	0.738	0.482	0.828 ± 0.011	2900	1600	0.706	0.472	0.826 ± 0.011
2800	1700	0.735	0.480	0.830 ± 0.011	2900	1700	0.705	0.472	0.826 ± 0.012
2800	1800	0.736	0.480	0.829 ± 0.010	2900	1800	0.698	0.467	0.834 ± 0.019
2800	1900	0.738	0.481	0.827 ± 0.009	2900	1900	0.699	0.468	0.833 ± 0.011
2800	2000	0.739	0.482	0.826 ± 0.009	2900	2000	0.698	0.467	0.834 ± 0.037
2800	2100	0.740	0.483	0.824 ± 0.012	2900	2100	0.710	0.475	0.819 ± 0.010
2800	2200	0.750	0.489	0.813 ± 0.012	2900	2200	0.706	0.472	0.824 ± 0.010
2800	2300	0.757	0.494	0.806 ± 0.010	2900	2300	0.712	0.476	0.817 ± 0.010
2800	2400	0.764	0.498	0.799 ± 0.010	2900	2400	0.718	0.480	0.811 ± 0.018
2800	2500	0.783	0.511	0.779 ± 0.009	2900	2500	0.727	0.486	0.801 ± 0.010
2800	2600	0.821	0.536	0.743 ± 0.017	2900	2600	0.748	0.500	0.778 ± 0.015
2800	2700	0.898	0.586	0.680 ± 0.013	2900	2700	0.782	0.523	0.744 ± 0.010
					2900	2800	0.846	0.566	0.688 ± 0.016

Table A.7. The 95% CL observed (Obs.) and expected (Exp.) exclusion limits (in fb) on the W_R production cross section times branching fraction for $W_R \to eejj$ as a function of W_R and N_e mass (in GeV) for $3000 \leq M_{W_R} \leq 3100$ GeV. The signal acceptance (Acc.) is also included for each (M_{W_R}, M_{N_e}) entry.

M_{W_R}	M_{N_e}	Obs.	Exp.	Acc.	M_{W_R}	M_{N_e}	Obs.	Exp.	Acc.
3000	100	134	90.2	0.030 ± 0.314	3100	100	134	94.2	0.036 ± 0.318
3000	200	18.8	12.6	0.131 ± 0.196	3100	200	20.6	14.4	0.145 ± 0.204
3000	300	5.90	3.96	0.256 ± 0.124	3100	300	6.52	4.57	0.261 ± 0.136
3000	400	2.94	1.97	0.382 ± 0.082	3100	400	3.12	2.19	0.387 ± 0.089
3000	500	1.86	1.25	0.496 ± 0.052	3100	500	1.97	1.38	0.489 ± 0.057
3000	600	1.37	0.923	0.586 ± 0.033	3100	600	1.42	0.996	0.578 ± 0.037
3000	700	1.13	0.756	0.648 ± 0.022	3100	700	1.15	0.806	0.646 ± 0.028
3000	800	0.984	0.661	0.695 ± 0.019	3100	800	0.989	0.693	0.695 ± 0.018
3000	900	0.891	0.598	0.729 ± 0.017	3100	900	0.888	0.622	0.733 ± 0.019
3000	1000	0.831	0.558	0.765 ± 0.013	3100	1000	0.823	0.576	0.752 ± 0.013
3000	1100	0.786	0.528	0.779 ± 0.017	3100	1100	0.771	0.540	0.774 ± 0.012
3000	1200	0.755	0.506	0.790 ± 0.013	3100	1200	0.740	0.518	0.792 ± 0.028
3000	1300	0.730	0.490	0.799 ± 0.018	3100	1300	0.713	0.499	0.803 ± 0.020
3000	1400	0.711	0.477	0.813 ± 0.012	3100	1400	0.689	0.482	0.813 ± 0.033
3000	1500	0.695	0.466	0.825 ± 0.010	3100	1500	0.671	0.470	0.815 ± 0.010
3000	1600	0.693	0.465	0.826 ± 0.015	3100	1600	0.664	0.465	0.827 ± 0.010
3000	1700	0.687	0.461	0.833 ± 0.009	3100	1700	0.659	0.461	0.833 ± 0.010
3000	1800	0.688	0.462	0.831 ± 0.011	3100	1800	0.658	0.461	0.833 ± 0.010
3000	1900	0.688	0.462	0.831 ± 0.022	3100	1900	0.659	0.461	0.832 ± 0.013
3000	2000	0.684	0.459	0.836 ± 0.011	3100	2000	0.654	0.458	0.838 ± 0.017
3000	2100	0.686	0.460	0.833 ± 0.010	3100	2100	0.659	0.461	0.831 ± 0.024
3000	2200	0.691	0.464	0.827 ± 0.040	3100	2200	0.657	0.460	0.834 ± 0.018
3000	2300	0.691	0.464	0.826 ± 0.011	3100	2300	0.659	0.462	0.830 ± 0.011
3000	2400	0.696	0.467	0.821 ± 0.013	3100	2400	0.664	0.465	0.825 ± 0.011
3000	2500	0.704	0.473	0.811 ± 0.015	3100	2500	0.670	0.469	0.817 ± 0.011
3000	2600	0.714	0.479	0.800 ± 0.009	3100	2600	0.674	0.472	0.813 ± 0.009
3000	2700	0.729	0.489	0.784 ± 0.010	3100	2700	0.683	0.478	0.801 ± 0.010
3000	2800	0.764	0.513	0.747 ± 0.011	3100	2800	0.701	0.491	0.781 ± 0.012
3000	2900	0.826	0.555	0.691 ± 0.013	3100	2900	0.730	0.511	0.750 ± 0.010
					3100	3000	0.789	0.553	0.693 ± 0.019

Table A.8. The 95% CL observed (Obs.) and expected (Exp.) exclusion limits (in fb) on the W_R production cross section times branching fraction for $W_R \to eejj$ as a function of W_R and N_e mass (in GeV) for $M_{W_R} = 3200\,GeV$. The signal acceptance (Acc.) is also included for each (M_{W_R}, M_{N_e}) entry.

M_{W_R}	M_{N_e}	Obs.	Exp.	Acc.
3200	100	139	95.7	0.039 ± 0.320
3200	200	23.5	16.3	0.147 ± 0.210
3200	300	7.35	5.08	0.277 ± 0.143
3200	400	3.56	2.46	0.391 ± 0.096
3200	500	2.19	1.51	0.485 ± 0.066
3200	600	1.57	1.08	0.574 ± 0.041
3200	700	1.25	0.861	0.639 ± 0.032
3200	800	1.06	0.731	0.690 ± 0.022
3200	900	0.936	0.646	0.721 ± 0.025
3200	1000	0.860	0.594	0.749 ± 0.019
3200	1100	0.804	0.555	0.774 ± 0.016
3200	1200	0.760	0.524	0.787 ± 0.012
3200	1300	0.731	0.504	0.800 ± 0.017
3200	1400	0.705	0.487	0.809 ± 0.021
3200	1500	0.684	0.472	0.818 ± 0.014
3200	1600	0.669	0.462	0.827 ± 0.014
3200	1700	0.667	0.461	0.828 ± 0.011
3200	1800	0.662	0.457	0.834 ± 0.019
3200	1900	0.661	0.456	0.835 ± 0.011
3200	2000	0.659	0.455	0.837 ± 0.033
3200	2100	0.664	0.459	0.831 ± 0.010
3200	2200	0.658	0.454	0.838 ± 0.018
3200	2300	0.662	0.457	0.833 ± 0.011
3200	2400	0.666	0.460	0.828 ± 0.010
3200	2500	0.671	0.463	0.822 ± 0.011
3200	2600	0.676	0.467	0.816 ± 0.010
3200	2700	0.678	0.468	0.813 ± 0.015
3200	2800	0.690	0.476	0.800 ± 0.010
3200	2900	0.709	0.489	0.778 ± 0.013
3200	3000	0.730	0.504	0.755 ± 0.012
3200	3100	0.794	0.548	0.695 ± 0.028

Table A.9. The 95% CL observed (Obs.) and expected (Exp.) exclusion limits (in fb) on the W_R production cross section times branching fraction for $W_R \to \mu\mu jj$ as a function of W_R and N_μ mass (in GeV) for $1000 \leq M_{W_R} \leq 1600\,\text{GeV}$. This signal acceptance (Acc.) is also included for each (M_{W_R}, M_{N_μ}) entry.

M_{W_R}	M_{N_μ}	Obs.	Exp.	Acc.	M_{W_R}	M_{N_μ}	Obs.	Exp.	Acc.
1000	100	79.0	53.1	0.0664 ± 0.0685	1400	100	26.9	44.7	0.037 ± 0.140
1000	200	18.1	12.1	0.283 ± 0.017	1400	200	4.17	6.93	0.224 ± 0.029
1000	300	12.4	8.31	0.435 ± 0.014	1400	300	2.31	3.83	0.410 ± 0.016
1000	400	10.4	7.00	0.539 ± 0.014	1400	400	1.79	2.97	0.533 ± 0.015
1000	500	9.53	6.40	0.586 ± 0.015	1400	500	1.58	2.63	0.618 ± 0.015
1000	600	9.12	6.13	0.612 ± 0.014	1400	600	1.49	2.48	0.676 ± 0.014
1000	700	9.16	6.15	0.609 ± 0.014	1400	700	1.43	2.38	0.702 ± 0.014
1000	800	9.83	6.61	0.567 ± 0.014	1400	800	1.40	2.32	0.720 ± 0.014
1000	900	13.0	8.71	0.430 ± 0.015	1400	900	1.40	2.32	0.721 ± 0.014
1100	100	72.9	57.5	0.0550 ± 0.0851	1400	1000	1.40	2.33	0.717 ± 0.014
1100	200	13.7	10.8	0.275 ± 0.020	1400	1100	1.43	2.37	0.705 ± 0.014
1100	300	8.18	6.46	0.428 ± 0.015	1400	1200	1.53	2.54	0.658 ± 0.015
1100	400	6.88	5.43	0.536 ± 0.014	1400	1300	1.82	3.02	0.553 ± 0.014
1100	500	6.47	5.10	0.599 ± 0.014	1500	100	22.8	43.5	0.033 ± 0.160
1100	600	6.17	4.87	0.647 ± 0.014	1500	200	3.26	6.20	0.205 ± 0.035
1100	700	6.10	4.82	0.654 ± 0.014	1500	300	1.60	3.05	0.391 ± 0.018
1100	800	6.29	4.96	0.635 ± 0.014	1500	400	1.23	2.34	0.528 ± 0.014
1100	900	6.67	5.26	0.599 ± 0.014	1500	500	1.09	2.08	0.611 ± 0.014
1100	1000	8.53	6.73	0.468 ± 0.015	1500	600	1.03	1.97	0.675 ± 0.014
1200	100	51.3	54.3	0.047 ± 0.103	1500	700	1.01	1.91	0.707 ± 0.014
1200	200	9.38	9.92	0.259 ± 0.022	1500	800	0.988	1.88	0.731 ± 0.014
1200	300	5.76	6.10	0.426 ± 0.015	1500	900	0.978	1.86	0.738 ± 0.014
1200	400	4.66	4.93	0.543 ± 0.014	1500	1000	0.968	1.84	0.746 ± 0.014
1200	500	4.19	4.43	0.617 ± 0.014	1500	1100	0.986	1.88	0.732 ± 0.014
1200	600	3.93	4.16	0.661 ± 0.014	1500	1200	1.02	1.95	0.705 ± 0.015
1200	700	3.86	4.08	0.674 ± 0.014	1500	1300	1.08	2.05	0.670 ± 0.014
1200	800	3.82	4.04	0.680 ± 0.014	1500	1400	1.27	2.42	0.568 ± 0.014
1200	900	3.90	4.13	0.666 ± 0.014	1600	100	22.1	40.2	0.029 ± 0.179
1200	1000	4.19	4.43	0.621 ± 0.014	1600	200	3.13	5.68	0.196 ± 0.041
1200	1100	5.23	5.53	0.498 ± 0.014	1600	300	1.55	2.81	0.382 ± 0.018
1300	100	32.5	45.4	0.042 ± 0.121	1600	400	1.16	2.11	0.517 ± 0.015
1300	200	5.13	7.18	0.239 ± 0.026	1600	500	1.01	1.83	0.612 ± 0.015
1300	300	2.76	3.86	0.428 ± 0.015	1600	600	0.924	1.68	0.668 ± 0.014
1300	400	2.21	3.09	0.543 ± 0.014	1600	700	0.901	1.64	0.713 ± 0.014
1300	500	2.03	2.84	0.622 ± 0.015	1600	800	0.877	1.59	0.737 ± 0.014
1300	600	1.96	2.74	0.674 ± 0.014	1600	900	0.861	1.57	0.751 ± 0.014
1300	700	1.93	2.71	0.688 ± 0.014	1600	1000	0.855	1.56	0.755 ± 0.014
1300	800	1.88	2.63	0.707 ± 0.015	1600	1100	0.857	1.56	0.754 ± 0.014
1300	900	1.90	2.65	0.702 ± 0.014	1600	1200	0.870	1.58	0.743 ± 0.014
1300	1000	1.93	2.70	0.690 ± 0.015	1600	1300	0.890	1.62	0.726 ± 0.014
1300	1100	2.07	2.89	0.644 ± 0.014	1600	1400	0.936	1.70	0.690 ± 0.014
1300	1200	2.51	3.51	0.531 ± 0.017	1600	1500	1.10	2.00	0.588 ± 0.014

Table A.10. The 95% CL observed (Obs.) and expected (Exp.) exclusion limits (in fb) on the W_R production cross section times branching fraction for $W_R \to \mu\mu jj$ as a function of W_R and N_μ mass (in GeV) for $1700 \leq M_{W_R} \leq 2000$ GeV. This signal acceptance (Acc.) is also included for each (M_{W_R}, M_{N_μ}) entry.

M_{W_R}	M_{N_μ}	Obs.	Exp.	Acc.	M_{W_R}	M_{N_μ}	Obs.	Exp.	Acc.
1700	100	28.6	44.3	0.028 ± 0.198	1900	100	38.2	45.2	0.020 ± 0.235
1700	200	3.53	5.46	0.179 ± 0.048	1900	200	4.32	5.11	0.148 ± 0.063
1700	300	1.53	2.36	0.364 ± 0.020	1900	300	1.80	2.12	0.327 ± 0.028
1700	400	1.09	1.68	0.507 ± 0.016	1900	400	1.22	1.45	0.482 ± 0.023
1700	500	0.929	1.44	0.601 ± 0.014	1900	500	1.02	1.21	0.587 ± 0.014
1700	600	0.869	1.35	0.664 ± 0.015	1900	600	0.922	1.09	0.659 ± 0.016
1700	700	0.853	1.32	0.709 ± 0.014	1900	700	0.879	1.04	0.703 ± 0.015
1700	800	0.827	1.28	0.743 ± 0.014	1900	800	0.857	1.01	0.738 ± 0.014
1700	900	0.815	1.26	0.759 ± 0.014	1900	900	0.838	0.991	0.759 ± 0.014
1700	1000	0.809	1.25	0.765 ± 0.014	1900	1000	0.824	0.974	0.774 ± 0.016
1700	1100	0.806	1.25	0.768 ± 0.015	1900	1100	0.820	0.970	0.777 ± 0.014
1700	1200	0.812	1.26	0.762 ± 0.014	1900	1200	0.818	0.967	0.780 ± 0.014
1700	1300	0.819	1.27	0.755 ± 0.014	1900	1300	0.816	0.964	0.782 ± 0.016
1700	1400	0.844	1.31	0.733 ± 0.014	1900	1400	0.817	0.966	0.781 ± 0.014
1700	1500	0.887	1.37	0.698 ± 0.015	1900	1500	0.835	0.987	0.763 ± 0.014
1700	1600	1.02	1.58	0.605 ± 0.014	1900	1600	0.848	1.00	0.752 ± 0.014
1800	100	31.5	43.8	0.023 ± 0.216	1900	1700	0.896	1.06	0.711 ± 0.015
1800	200	4.03	5.61	0.163 ± 0.055	1900	1800	1.02	1.21	0.622 ± 0.015
1800	300	1.77	2.46	0.343 ± 0.022	2000	100	41.5	46.1	0.019 ± 0.253
1800	400	1.24	1.72	0.494 ± 0.015	2000	200	4.92	5.46	0.137 ± 0.068
1800	500	1.02	1.42	0.589 ± 0.019	2000	300	1.93	2.15	0.308 ± 0.028
1800	600	0.922	1.28	0.657 ± 0.014	2000	400	1.25	1.39	0.461 ± 0.017
1800	700	0.872	1.21	0.709 ± 0.014	2000	500	0.992	1.10	0.573 ± 0.015
1800	800	0.841	1.17	0.738 ± 0.014	2000	600	0.878	0.975	0.648 ± 0.014
1800	900	0.833	1.16	0.756 ± 0.014	2000	700	0.826	0.918	0.696 ± 0.014
1800	1000	0.820	1.14	0.768 ± 0.015	2000	800	0.790	0.878	0.733 ± 0.014
1800	1100	0.810	1.13	0.777 ± 0.014	2000	900	0.775	0.861	0.757 ± 0.014
1800	1200	0.811	1.13	0.777 ± 0.014	2000	1000	0.764	0.849	0.776 ± 0.014
1800	1300	0.812	1.13	0.775 ± 0.014	2000	1100	0.755	0.839	0.785 ± 0.018
1800	1400	0.825	1.15	0.764 ± 0.014	2000	1200	0.746	0.829	0.794 ± 0.014
1800	1500	0.848	1.18	0.743 ± 0.014	2000	1300	0.744	0.827	0.796 ± 0.015
1800	1600	0.894	1.24	0.704 ± 0.014	2000	1400	0.752	0.835	0.788 ± 0.014
1800	1700	1.03	1.44	0.610 ± 0.015	2000	1500	0.758	0.842	0.782 ± 0.015
					2000	1600	0.767	0.852	0.772 ± 0.014
					2000	1700	0.781	0.868	0.758 ± 0.015
					2000	1800	0.828	0.920	0.715 ± 0.015
					2000	1900	0.946	1.05	0.627 ± 0.014

Table A.11. The 95% CL observed (Obs.) and expected (Exp.) exclusion limits (in fb) on the W_R production cross section times branching fraction for $W_R \to \mu\mu jj$ as a function of W_R and N_μ mass (in GeV) for $2100 \leq M_{W_R} \leq 2300$ GeV. This signal acceptance (Acc.) is also included for each (M_{W_R}, M_{N_μ}) entry.

M_{W_R}	M_{N_μ}	Obs.	Exp.	Acc.	M_{W_R}	M_{N_μ}	Obs.	Exp.	Acc.
2100	100	51.7	54.3	0.020 ± 0.271	2300	100	54.0	49.5	0.018 ± 0.306
2100	200	5.70	5.99	0.129 ± 0.076	2300	200	5.59	5.12	0.121 ± 0.095
2100	300	2.22	2.33	0.294 ± 0.029	2300	300	2.11	1.94	0.275 ± 0.037
2100	400	1.38	1.45	0.448 ± 0.018	2300	400	1.27	1.17	0.413 ± 0.020
2100	500	1.07	1.12	0.566 ± 0.025	2300	500	0.987	0.905	0.533 ± 0.016
2100	600	0.917	0.964	0.640 ± 0.014	2300	600	0.862	0.791	0.632 ± 0.015
2100	700	0.839	0.881	0.696 ± 0.014	2300	700	0.788	0.722	0.675 ± 0.014
2100	800	0.794	0.835	0.733 ± 0.015	2300	800	0.752	0.690	0.723 ± 0.014
2100	900	0.767	0.806	0.749 ± 0.014	2300	900	0.734	0.673	0.750 ± 0.014
2100	1000	0.751	0.789	0.775 ± 0.014	2300	1000	0.714	0.654	0.769 ± 0.014
2100	1100	0.740	0.777	0.783 ± 0.014	2300	1100	0.703	0.644	0.784 ± 0.014
2100	1200	0.734	0.771	0.789 ± 0.014	2300	1200	0.701	0.643	0.794 ± 0.014
2100	1300	0.725	0.762	0.798 ± 0.014	2300	1300	0.693	0.636	0.802 ± 0.014
2100	1400	0.728	0.764	0.796 ± 0.014	2300	1400	0.690	0.633	0.806 ± 0.014
2100	1500	0.729	0.766	0.794 ± 0.014	2300	1500	0.690	0.632	0.806 ± 0.015
2100	1600	0.735	0.773	0.787 ± 0.014	2300	1600	0.690	0.633	0.806 ± 0.015
2100	1700	0.749	0.787	0.773 ± 0.014	2300	1700	0.695	0.637	0.800 ± 0.014
2100	1800	0.768	0.807	0.754 ± 0.014	2300	1800	0.700	0.642	0.795 ± 0.014
2100	1900	0.801	0.841	0.723 ± 0.014	2300	1900	0.709	0.650	0.785 ± 0.014
2100	2000	0.906	0.952	0.639 ± 0.014	2300	2000	0.731	0.671	0.760 ± 0.016
2200	100	53.2	49.5	0.018 ± 0.288	2300	2100	0.764	0.700	0.728 ± 0.014
2200	200	6.05	5.62	0.123 ± 0.085	2300	2200	0.853	0.782	0.652 ± 0.014
2200	300	2.36	2.20	0.279 ± 0.033					
2200	400	1.50	1.40	0.435 ± 0.019					
2200	500	1.17	1.09	0.547 ± 0.016					
2200	600	1.01	0.941	0.629 ± 0.015					
2200	700	0.919	0.854	0.689 ± 0.014					
2200	800	0.865	0.804	0.721 ± 0.014					
2200	900	0.832	0.774	0.754 ± 0.014					
2200	1000	0.808	0.751	0.775 ± 0.014					
2200	1100	0.791	0.736	0.783 ± 0.015					
2200	1200	0.782	0.727	0.792 ± 0.040					
2200	1300	0.770	0.716	0.804 ± 0.015					
2200	1400	0.780	0.725	0.794 ± 0.015					
2200	1500	0.774	0.719	0.801 ± 0.015					
2200	1600	0.775	0.720	0.800 ± 0.015					
2200	1700	0.782	0.727	0.792 ± 0.014					
2200	1800	0.795	0.739	0.780 ± 0.017					
2200	1900	0.814	0.756	0.762 ± 0.016					
2200	2000	0.854	0.794	0.725 ± 0.015					
2200	2100	0.955	0.888	0.649 ± 0.016					

Table A.12. The 95% CL observed (Obs.) and expected (Exp.) exclusion limits (in fb) on the W_R production cross section times branching fraction for $W_R \to \mu\mu jj$ as a function of W_R and N_μ mass (in GeV) for $2400 \leq M_{W_R} \leq 2500$ GeV. This signal acceptance (Acc.) is also included for each (M_{W_R}, M_{N_μ}) entry.

M_{W_R}	M_{N_μ}	Obs.	Exp.	Acc.	M_{W_R}	M_{N_μ}	Obs.	Exp.	Acc.
2400	100	63.9	56.1	0.020 ± 0.325	2500	100	72.8	62.7	0.021 ± 0.345
2400	200	6.12	5.36	0.116 ± 0.105	2500	200	6.78	5.83	0.112 ± 0.116
2400	300	2.20	1.93	0.258 ± 0.041	2500	300	2.33	2.01	0.245 ± 0.047
2400	400	1.29	1.13	0.407 ± 0.022	2500	400	1.33	1.14	0.389 ± 0.024
2400	500	0.961	0.843	0.526 ± 0.016	2500	500	0.944	0.813	0.515 ± 0.017
2400	600	0.804	0.705	0.614 ± 0.015	2500	600	0.784	0.675	0.602 ± 0.016
2400	700	0.735	0.645	0.675 ± 0.014	2500	700	0.697	0.600	0.665 ± 0.014
2400	800	0.690	0.605	0.718 ± 0.014	2500	800	0.647	0.557	0.714 ± 0.014
2400	900	0.702	0.616	0.745 ± 0.016	2500	900	0.611	0.526	0.738 ± 0.014
2400	1000	0.681	0.597	0.768 ± 0.014	2500	1000	0.597	0.514	0.766 ± 0.014
2400	1100	0.653	0.572	0.784 ± 0.014	2500	1100	0.581	0.500	0.781 ± 0.014
2400	1200	0.635	0.557	0.800 ± 0.014	2500	1200	0.574	0.494	0.794 ± 0.014
2400	1300	0.631	0.553	0.805 ± 0.015	2500	1300	0.569	0.490	0.802 ± 0.014
2400	1400	0.632	0.554	0.804 ± 0.014	2500	1400	0.565	0.486	0.807 ± 0.014
2400	1500	0.630	0.552	0.806 ± 0.014	2500	1500	0.561	0.483	0.813 ± 0.015
2400	1600	0.626	0.549	0.811 ± 0.015	2500	1600	0.556	0.479	0.820 ± 0.016
2400	1700	0.627	0.550	0.810 ± 0.022	2500	1700	0.564	0.485	0.809 ± 0.019
2400	1800	0.634	0.556	0.802 ± 0.021	2500	1800	0.566	0.487	0.806 ± 0.018
2400	1900	0.639	0.561	0.795 ± 0.014	2500	1900	0.564	0.485	0.810 ± 0.016
2400	2000	0.646	0.566	0.786 ± 0.014	2500	2000	0.570	0.491	0.800 ± 0.014
2400	2100	0.660	0.579	0.769 ± 0.014	2500	2100	0.576	0.496	0.791 ± 0.014
2400	2200	0.692	0.607	0.734 ± 0.014	2500	2200	0.595	0.512	0.766 ± 0.016
2400	2300	0.771	0.676	0.659 ± 0.017	2500	2300	0.621	0.534	0.735 ± 0.015
					2500	2400	0.691	0.594	0.661 ± 0.015

Table A.13. The 95% CL observed (Obs.) and expected (Exp.) exclusion limits (in fb) on the W_R production cross section times branching fraction for $W_R \to \mu\mu jj$ as a function of W_R and N_μ mass (in GeV) for $2600 \leq M_{W_R} \leq 2700$ GeV. This signal acceptance (Acc.) is also included for each (M_{W_R}, M_{N_μ}) entry.

M_{W_R}	M_{N_μ}	Obs.	Exp.	Acc.	M_{W_R}	M_{N_μ}	Obs.	Exp.	Acc.
2600	100	74.3	62.3	0.019 ± 0.359	2700	100	68.8	59.9	0.021 ± 0.378
2600	200	7.52	6.30	0.110 ± 0.127	2700	200	7.51	6.55	0.114 ± 0.142
2600	300	2.53	2.12	0.236 ± 0.053	2700	300	2.52	2.20	0.237 ± 0.060
2600	400	1.37	1.15	0.379 ± 0.029	2700	400	1.34	1.17	0.369 ± 0.032
2600	500	0.963	0.807	0.498 ± 0.018	2700	500	0.923	0.805	0.487 ± 0.019
2600	600	0.777	0.651	0.594 ± 0.015	2700	600	0.728	0.635	0.589 ± 0.015
2600	700	0.680	0.570	0.663 ± 0.014	2700	700	0.646	0.564	0.652 ± 0.015
2600	800	0.627	0.526	0.707 ± 0.014	2700	800	0.602	0.525	0.701 ± 0.014
2600	900	0.593	0.497	0.732 ± 0.014	2700	900	0.558	0.487	0.733 ± 0.014
2600	1000	0.567	0.475	0.760 ± 0.014	2700	1000	0.519	0.453	0.761 ± 0.014
2600	1100	0.547	0.458	0.773 ± 0.015	2700	1100	0.504	0.439	0.775 ± 0.019
2600	1200	0.551	0.462	0.794 ± 0.014	2700	1200	0.475	0.414	0.789 ± 0.016
2600	1300	0.535	0.448	0.799 ± 0.014	2700	1300	0.479	0.417	0.802 ± 0.016
2600	1400	0.531	0.445	0.805 ± 0.015	2700	1400	0.487	0.424	0.806 ± 0.025
2600	1500	0.529	0.443	0.808 ± 0.014	2700	1500	0.482	0.421	0.813 ± 0.014
2600	1600	0.522	0.438	0.818 ± 0.017	2700	1600	0.479	0.418	0.818 ± 0.014
2600	1700	0.522	0.438	0.819 ± 0.014	2700	1700	0.481	0.419	0.816 ± 0.014
2600	1800	0.525	0.440	0.814 ± 0.035	2700	1800	0.479	0.417	0.820 ± 0.015
2600	1900	0.526	0.441	0.812 ± 0.014	2700	1900	0.480	0.419	0.817 ± 0.014
2600	2000	0.529	0.444	0.807 ± 0.014	2700	2000	0.480	0.419	0.816 ± 0.014
2600	2100	0.533	0.446	0.802 ± 0.014	2700	2100	0.482	0.420	0.813 ± 0.016
2600	2200	0.540	0.452	0.792 ± 0.016	2700	2200	0.491	0.428	0.799 ± 0.014
2600	2300	0.553	0.464	0.772 ± 0.015	2700	2300	0.497	0.433	0.790 ± 0.022
2600	2400	0.582	0.487	0.735 ± 0.015	2700	2400	0.509	0.444	0.771 ± 0.014
2600	2500	0.645	0.541	0.662 ± 0.015	2700	2500	0.533	0.465	0.736 ± 0.015
					2700	2600	0.583	0.508	0.673 ± 0.017

Table A.14. The 95% CL observed (Obs.) and expected (Exp.) exclusion limits (in fb) on the W_R production cross section times branching fraction for $W_R \to \mu\mu jj$ as a function of W_R and N_μ mass (in GeV) for $2800 \leq M_{W_R} \leq 2900$ GeV. This signal acceptance (Acc.) is also included for each (M_{W_R}, M_{N_μ}) entry.

M_{W_R}	M_{N_μ}	Obs.	Exp.	Acc.	M_{W_R}	M_{N_μ}	Obs.	Exp.	Acc.
2800	100	71.6	63.4	0.023 ± 0.398	2900	100	73.4	63.2	0.024 ± 0.412
2800	200	8.59	7.62	0.116 ± 0.154	2900	200	9.90	8.53	0.117 ± 0.169
2800	300	2.87	2.55	0.235 ± 0.066	2900	300	3.25	2.80	0.227 ± 0.079
2800	400	1.49	1.32	0.360 ± 0.036	2900	400	1.65	1.42	0.365 ± 0.045
2800	500	0.992	0.879	0.482 ± 0.021	2900	500	1.07	0.919	0.471 ± 0.040
2800	600	0.774	0.686	0.576 ± 0.016	2900	600	0.817	0.703	0.570 ± 0.017
2800	700	0.654	0.580	0.645 ± 0.015	2900	700	0.688	0.593	0.637 ± 0.015
2800	800	0.604	0.535	0.691 ± 0.014	2900	800	0.608	0.524	0.683 ± 0.014
2800	900	0.544	0.482	0.724 ± 0.014	2900	900	0.558	0.481	0.721 ± 0.014
2800	1000	0.516	0.458	0.752 ± 0.017	2900	1000	0.527	0.454	0.744 ± 0.014
2800	1100	0.494	0.438	0.768 ± 0.015	2900	1100	0.503	0.433	0.767 ± 0.014
2800	1200	0.478	0.424	0.789 ± 0.015	2900	1200	0.485	0.417	0.784 ± 0.018
2800	1300	0.464	0.412	0.800 ± 0.014	2900	1300	0.470	0.405	0.800 ± 0.014
2800	1400	0.457	0.406	0.807 ± 0.014	2900	1400	0.461	0.397	0.807 ± 0.015
2800	1500	0.453	0.402	0.815 ± 0.015	2900	1500	0.455	0.392	0.810 ± 0.014
2800	1600	0.450	0.399	0.820 ± 0.014	2900	1600	0.450	0.387	0.820 ± 0.015
2800	1700	0.448	0.397	0.824 ± 0.014	2900	1700	0.450	0.388	0.820 ± 0.015
2800	1800	0.449	0.398	0.823 ± 0.014	2900	1800	0.446	0.384	0.827 ± 0.028
2800	1900	0.450	0.399	0.821 ± 0.016	2900	1900	0.447	0.385	0.825 ± 0.017
2800	2000	0.450	0.399	0.820 ± 0.014	2900	2000	0.445	0.383	0.828 ± 0.017
2800	2100	0.451	0.400	0.818 ± 0.014	2900	2100	0.454	0.391	0.813 ± 0.014
2800	2200	0.457	0.406	0.807 ± 0.015	2900	2200	0.451	0.388	0.819 ± 0.016
2800	2300	0.461	0.409	0.801 ± 0.015	2900	2300	0.454	0.391	0.812 ± 0.016
2800	2400	0.465	0.412	0.794 ± 0.015	2900	2400	0.458	0.395	0.805 ± 0.014
2800	2500	0.477	0.423	0.774 ± 0.014	2900	2500	0.464	0.399	0.796 ± 0.014
2800	2600	0.500	0.443	0.739 ± 0.015	2900	2600	0.477	0.411	0.773 ± 0.014
2800	2700	0.546	0.484	0.676 ± 0.016	2900	2700	0.499	0.429	0.740 ± 0.014
					2900	2800	0.539	0.464	0.684 ± 0.020

Table A.15. The 95% CL observed (Obs.) and expected (Exp.) exclusion limits (in fb) on the W_R production cross section times branching fraction for $W_R \to \mu\mu jj$ as a function of W_R and N_μ mass (in GeV) for $3000 \leq M_{W_R} \leq 3100$ GeV. This signal acceptance (Acc.) is also included for each (M_{W_R}, M_{N_μ}) entry.

M_{W_R}	M_{N_μ}	Obs.	Exp.	Acc.	M_{W_R}	M_{N_μ}	Obs.	Exp.	Acc.
3000	100	72.8	63.2	0.024 ± 0.428	3100	100	75.6	65.9	0.029 ± 0.439
3000	200	11.3	9.79	0.117 ± 0.185	3100	200	12.9	11.2	0.130 ± 0.197
3000	300	3.59	3.12	0.234 ± 0.094	3100	300	4.15	3.62	0.240 ± 0.104
3000	400	1.81	1.57	0.356 ± 0.052	3100	400	2.01	1.75	0.359 ± 0.060
3000	500	1.16	1.01	0.469 ± 0.031	3100	500	1.28	1.12	0.461 ± 0.037
3000	600	0.864	0.750	0.561 ± 0.019	3100	600	0.935	0.815	0.552 ± 0.024
3000	700	0.714	0.620	0.627 ± 0.016	3100	700	0.763	0.665	0.623 ± 0.021
3000	800	0.628	0.545	0.675 ± 0.015	3100	800	0.660	0.575	0.674 ± 0.016
3000	900	0.571	0.496	0.713 ± 0.016	3100	900	0.595	0.519	0.715 ± 0.015
3000	1000	0.535	0.465	0.750 ± 0.014	3100	1000	0.554	0.483	0.738 ± 0.015
3000	1100	0.507	0.440	0.767 ± 0.015	3100	1100	0.521	0.454	0.762 ± 0.018
3000	1200	0.488	0.424	0.780 ± 0.020	3100	1200	0.500	0.436	0.781 ± 0.017
3000	1300	0.473	0.410	0.790 ± 0.015	3100	1300	0.483	0.421	0.793 ± 0.015
3000	1400	0.461	0.400	0.805 ± 0.018	3100	1400	0.467	0.407	0.805 ± 0.023
3000	1500	0.451	0.391	0.816 ± 0.016	3100	1500	0.455	0.397	0.808 ± 0.018
3000	1600	0.449	0.390	0.819 ± 0.014	3100	1600	0.451	0.393	0.818 ± 0.032
3000	1700	0.445	0.387	0.826 ± 0.017	3100	1700	0.448	0.390	0.825 ± 0.017
3000	1800	0.446	0.387	0.824 ± 0.018	3100	1800	0.447	0.390	0.825 ± 0.032
3000	1900	0.446	0.387	0.824 ± 0.015	3100	1900	0.448	0.390	0.825 ± 0.018
3000	2000	0.443	0.385	0.829 ± 0.031	3100	2000	0.445	0.387	0.831 ± 0.014
3000	2100	0.444	0.385	0.828 ± 0.015	3100	2100	0.448	0.390	0.825 ± 0.041
3000	2200	0.448	0.389	0.820 ± 0.015	3100	2200	0.446	0.389	0.828 ± 0.015
3000	2300	0.448	0.389	0.821 ± 0.026	3100	2300	0.448	0.390	0.824 ± 0.020
3000	2400	0.451	0.392	0.815 ± 0.014	3100	2400	0.451	0.393	0.819 ± 0.015
3000	2500	0.456	0.396	0.806 ± 0.017	3100	2500	0.455	0.396	0.812 ± 0.015
3000	2600	0.462	0.401	0.795 ± 0.015	3100	2600	0.457	0.399	0.807 ± 0.016
3000	2700	0.471	0.409	0.780 ± 0.014	3100	2700	0.463	0.404	0.797 ± 0.017
3000	2800	0.494	0.429	0.744 ± 0.014	3100	2800	0.475	0.414	0.777 ± 0.014
3000	2900	0.535	0.464	0.688 ± 0.024	3100	2900	0.494	0.431	0.747 ± 0.015
					3100	3000	0.535	0.466	0.690 ± 0.019

Table A.16. The 95% CL observed (Obs.) and expected (Exp.) exclusion limits (in fb) on the W_R production cross section times branching fraction for $W_R \rightarrow \mu\mu jj$ as a function of W_R and N_μ mass (in GeV) for $M_{W_R} = 3200\,\text{GeV}$. This signal acceptance (Acc.) is also included for each (M_{W_R}, M_{N_μ}) entry.

M_{W_R}	M_{N_μ}	Obs.	Exp.	Acc.
3200	100	75.8	66.0	0.031 ± 0.449
3200	200	14.4	12.5	0.131 ± 0.206
3200	300	4.58	3.99	0.254 ± 0.111
3200	400	2.24	1.95	0.363 ± 0.070
3200	500	1.39	1.21	0.458 ± 0.050
3200	600	1.01	0.877	0.546 ± 0.029
3200	700	0.808	0.704	0.614 ± 0.020
3200	800	0.691	0.602	0.668 ± 0.016
3200	900	0.614	0.535	0.703 ± 0.018
3200	1000	0.567	0.494	0.734 ± 0.014
3200	1100	0.531	0.463	0.762 ± 0.027
3200	1200	0.503	0.438	0.776 ± 0.015
3200	1300	0.485	0.422	0.790 ± 0.026
3200	1400	0.468	0.408	0.799 ± 0.015
3200	1500	0.455	0.396	0.808 ± 0.016
3200	1600	0.445	0.388	0.819 ± 0.015
3200	1700	0.444	0.387	0.820 ± 0.030
3200	1800	0.441	0.384	0.827 ± 0.018
3200	1900	0.440	0.383	0.829 ± 0.031
3200	2000	0.438	0.382	0.832 ± 0.023
3200	2100	0.442	0.385	0.825 ± 0.018
3200	2200	0.438	0.382	0.831 ± 0.019
3200	2300	0.441	0.385	0.826 ± 0.019
3200	2400	0.443	0.386	0.822 ± 0.015
3200	2500	0.446	0.389	0.817 ± 0.018
3200	2600	0.449	0.391	0.811 ± 0.017
3200	2700	0.451	0.393	0.808 ± 0.021
3200	2800	0.459	0.399	0.795 ± 0.021
3200	2900	0.471	0.410	0.774 ± 0.022
3200	3000	0.485	0.423	0.751 ± 0.015
3200	3100	0.527	0.459	0.691 ± 0.039

Table A.17. The 95% CL observed (Obs.) and expected (Exp.) exclusion limits (in fb) on the W_R production cross section times branching fraction for $W_R \to (ee + \mu\mu)jj$ as a function of W_R and N_ℓ mass (in GeV) for $1000 \leq M_{W_R} \leq 1600$ GeV. The signal acceptance (Acc.) is also included for each (M_{W_R}, M_{N_ℓ}) entry.

M_{W_R}	M_{N_ℓ}	Obs.	Exp.	Acc.	M_{W_R}	M_{N_ℓ}	Obs.	Exp.	Acc.
1000	100	117	88.5	0.0695 ± 0.0650	1400	100	54.0	71.9	0.040 ± 0.109
1000	200	26.3	19.9	0.291 ± 0.018	1400	200	8.26	11.0	0.232 ± 0.034
1000	300	17.8	13.5	0.441 ± 0.010	1400	300	4.52	6.02	0.419 ± 0.016
1000	400	15.0	11.3	0.544 ± 0.009	1400	400	3.49	4.64	0.541 ± 0.011
1000	500	13.7	10.3	0.591 ± 0.009	1400	500	3.07	4.08	0.624 ± 0.009
1000	600	13.1	9.90	0.616 ± 0.008	1400	600	2.89	3.84	0.682 ± 0.009
1000	700	13.1	9.94	0.613 ± 0.008	1400	700	2.77	3.69	0.707 ± 0.009
1000	800	14.1	10.7	0.570 ± 0.008	1400	800	2.70	3.60	0.725 ± 0.008
1000	900	18.6	14.1	0.433 ± 0.009	1400	900	2.70	3.59	0.725 ± 0.008
1100	100	150	98.2	0.0582 ± 0.0760	1400	1000	2.71	3.61	0.721 ± 0.008
1100	200	27.8	18.1	0.282 ± 0.021	1400	1100	2.76	3.68	0.708 ± 0.008
1100	300	16.4	10.7	0.435 ± 0.011	1400	1200	2.96	3.93	0.661 ± 0.009
1100	400	13.8	9.00	0.542 ± 0.009	1400	1300	3.52	4.68	0.555 ± 0.009
1100	500	12.9	8.43	0.604 ± 0.009	1500	100	46.7	69.1	0.035 ± 0.119
1100	600	12.3	8.04	0.651 ± 0.009	1500	200	6.57	9.72	0.212 ± 0.039
1100	700	12.2	7.95	0.659 ± 0.008	1500	300	3.20	4.74	0.401 ± 0.018
1100	800	12.5	8.19	0.639 ± 0.009	1500	400	2.44	3.61	0.536 ± 0.011
1100	900	13.3	8.68	0.602 ± 0.009	1500	500	2.15	3.19	0.618 ± 0.009
1100	1000	17.0	11.1	0.470 ± 0.009	1500	600	2.04	3.01	0.680 ± 0.009
1200	100	108	89.9	0.0495 ± 0.0869	1500	700	1.98	2.92	0.711 ± 0.009
1200	200	19.5	16.2	0.267 ± 0.025	1500	800	1.94	2.87	0.735 ± 0.009
1200	300	11.9	9.84	0.434 ± 0.012	1500	900	1.92	2.84	0.742 ± 0.008
1200	400	9.54	7.92	0.549 ± 0.009	1500	1000	1.90	2.81	0.750 ± 0.008
1200	500	8.56	7.10	0.622 ± 0.009	1500	1100	1.94	2.87	0.735 ± 0.008
1200	600	8.01	6.65	0.666 ± 0.008	1500	1200	2.01	2.98	0.708 ± 0.009
1200	700	7.86	6.52	0.679 ± 0.008	1500	1300	2.11	3.13	0.673 ± 0.008
1200	800	7.79	6.46	0.684 ± 0.008	1500	1400	2.50	3.69	0.570 ± 0.008
1200	900	7.95	6.60	0.670 ± 0.009	1600	100	46.4	67.3	0.031 ± 0.130
1200	1000	8.53	7.08	0.624 ± 0.009	1600	200	6.48	9.39	0.204 ± 0.044
1200	1100	10.6	8.83	0.500 ± 0.008	1600	300	3.17	4.59	0.392 ± 0.020
1300	100	64.6	72.0	0.0445 ± 0.0977	1600	400	2.36	3.43	0.527 ± 0.012
1300	200	10.1	11.2	0.246 ± 0.030	1600	500	2.04	2.96	0.620 ± 0.011
1300	300	5.36	5.97	0.437 ± 0.014	1600	600	1.87	2.71	0.674 ± 0.009
1300	400	4.26	4.75	0.551 ± 0.010	1600	700	1.82	2.64	0.718 ± 0.008
1300	500	3.90	4.35	0.628 ± 0.009	1600	800	1.77	2.56	0.742 ± 0.009
1300	600	3.76	4.19	0.679 ± 0.009	1600	900	1.74	2.52	0.755 ± 0.008
1300	700	3.71	4.13	0.694 ± 0.008	1600	1000	1.72	2.50	0.759 ± 0.008
1300	800	3.61	4.02	0.712 ± 0.009	1600	1100	1.73	2.50	0.758 ± 0.008
1300	900	3.64	4.05	0.706 ± 0.008	1600	1200	1.75	2.54	0.746 ± 0.008
1300	1000	3.70	4.12	0.694 ± 0.009	1600	1300	1.79	2.60	0.729 ± 0.008
1300	1100	3.96	4.42	0.647 ± 0.008	1600	1400	1.89	2.74	0.692 ± 0.009
1300	1200	4.81	5.36	0.534 ± 0.009	1600	1500	2.22	3.21	0.590 ± 0.009

Table A.18. The 95% CL observed (Obs.) and expected (Exp.) exclusion limits (in fb) on the W_R production cross section times branching fraction for $W_R \to (ee + \mu\mu)jj$ as a function of W_R and N_ℓ mass (in GeV) for $1700 \leq M_{W_R} \leq 2000$ GeV. The signal acceptance (Acc.) is also included for each (M_{W_R}, M_{N_ℓ}) entry.

M_{W_R}	M_{N_ℓ}	Obs.	Exp.	Acc.	M_{W_R}	M_{N_ℓ}	Obs.	Exp.	Acc.
1700	100	60.3	75.0	0.029 ± 0.140	1900	100	117	69.5	0.022 ± 0.159
1700	200	7.31	9.09	0.187 ± 0.049	1900	200	13.0	7.71	0.154 ± 0.061
1700	300	3.13	3.90	0.373 ± 0.023	1900	300	5.37	3.17	0.338 ± 0.031
1700	400	2.21	2.75	0.516 ± 0.014	1900	400	3.63	2.14	0.492 ± 0.018
1700	500	1.88	2.34	0.609 ± 0.010	1900	500	3.01	1.78	0.596 ± 0.015
1700	600	1.76	2.19	0.670 ± 0.009	1900	600	2.71	1.60	0.667 ± 0.010
1700	700	1.72	2.14	0.714 ± 0.009	1900	700	2.58	1.52	0.709 ± 0.009
1700	800	1.67	2.07	0.747 ± 0.009	1900	800	2.51	1.48	0.743 ± 0.009
1700	900	1.64	2.04	0.763 ± 0.008	1900	900	2.45	1.45	0.764 ± 0.009
1700	1000	1.63	2.03	0.770 ± 0.008	1900	1000	2.41	1.42	0.778 ± 0.010
1700	1100	1.62	2.02	0.772 ± 0.009	1900	1100	2.40	1.42	0.781 ± 0.009
1700	1200	1.63	2.03	0.766 ± 0.008	1900	1200	2.39	1.41	0.783 ± 0.008
1700	1300	1.65	2.05	0.758 ± 0.009	1900	1300	2.38	1.41	0.785 ± 0.009
1700	1400	1.70	2.11	0.736 ± 0.008	1900	1400	2.39	1.41	0.784 ± 0.008
1700	1500	1.79	2.22	0.700 ± 0.009	1900	1500	2.44	1.44	0.767 ± 0.008
1700	1600	2.06	2.56	0.607 ± 0.009	1900	1600	2.48	1.47	0.754 ± 0.008
1800	100	81.1	71.6	0.025 ± 0.150	1900	1700	2.62	1.55	0.713 ± 0.009
1800	200	10.2	9.02	0.170 ± 0.055	1900	1800	2.99	1.77	0.624 ± 0.009
1800	300	4.45	3.93	0.353 ± 0.025	2000	100	137	67.6	0.021 ± 0.169
1800	400	3.08	2.72	0.504 ± 0.015	2000	200	15.9	7.84	0.143 ± 0.065
1800	500	2.52	2.23	0.597 ± 0.013	2000	300	6.20	3.06	0.318 ± 0.032
1800	600	2.28	2.01	0.664 ± 0.009	2000	400	3.97	1.96	0.472 ± 0.018
1800	700	2.15	1.90	0.715 ± 0.009	2000	500	3.14	1.55	0.583 ± 0.012
1800	800	2.07	1.83	0.743 ± 0.008	2000	600	2.77	1.36	0.656 ± 0.010
1800	900	2.05	1.81	0.760 ± 0.008	2000	700	2.60	1.28	0.702 ± 0.009
1800	1000	2.02	1.78	0.772 ± 0.009	2000	800	2.48	1.22	0.738 ± 0.009
1800	1100	1.99	1.76	0.781 ± 0.010	2000	900	2.43	1.20	0.762 ± 0.008
1800	1200	1.99	1.76	0.780 ± 0.008	2000	1000	2.40	1.18	0.780 ± 0.008
1800	1300	2.00	1.76	0.779 ± 0.008	2000	1100	2.37	1.17	0.788 ± 0.010
1800	1400	2.03	1.79	0.766 ± 0.008	2000	1200	2.34	1.15	0.798 ± 0.008
1800	1500	2.08	1.84	0.746 ± 0.008	2000	1300	2.33	1.15	0.799 ± 0.009
1800	1600	2.20	1.94	0.706 ± 0.010	2000	1400	2.36	1.16	0.792 ± 0.008
1800	1700	2.54	2.24	0.612 ± 0.009	2000	1500	2.37	1.17	0.785 ± 0.009
					2000	1600	2.40	1.19	0.776 ± 0.008
					2000	1700	2.45	1.21	0.761 ± 0.009
					2000	1800	2.59	1.28	0.718 ± 0.009
					2000	1900	2.96	1.46	0.629 ± 0.009

Table A.19. The 95% CL observed (Obs.) and expected (Exp.) exclusion limits (in fb) on the W_R production cross section times branching fraction for $W_R \to (ee + \mu\mu)jj$ as a function of W_R and N_ℓ mass (in GeV) for $2100 \leq M_{W_R} \leq 2300$ GeV. The signal acceptance (Acc.) is also included for each (M_{W_R}, M_{N_ℓ}) entry.

M_{W_R}	M_{N_ℓ}	Obs.	Exp.	Acc.	M_{W_R}	M_{N_ℓ}	Obs.	Exp.	Acc.
2100	100	172	79.3	0.022 ± 0.178	2300	100	147	77.7	0.020 ± 0.197
2100	200	18.5	8.55	0.135 ± 0.070	2300	200	14.8	7.81	0.127 ± 0.082
2100	300	7.14	3.30	0.305 ± 0.034	2300	300	5.57	2.94	0.286 ± 0.041
2100	400	4.41	2.04	0.458 ± 0.019	2300	400	3.33	1.76	0.425 ± 0.023
2100	500	3.39	1.57	0.576 ± 0.016	2300	500	2.56	1.35	0.543 ± 0.015
2100	600	2.90	1.34	0.648 ± 0.010	2300	600	2.23	1.18	0.641 ± 0.011
2100	700	2.65	1.22	0.703 ± 0.009	2300	700	2.03	1.07	0.683 ± 0.010
2100	800	2.50	1.16	0.739 ± 0.009	2300	800	1.94	1.02	0.730 ± 0.009
2100	900	2.42	1.12	0.754 ± 0.009	2300	900	1.89	0.994	0.755 ± 0.009
2100	1000	2.36	1.09	0.780 ± 0.008	2300	1000	1.83	0.966	0.774 ± 0.008
2100	1100	2.33	1.07	0.787 ± 0.008	2300	1100	1.80	0.950	0.788 ± 0.009
2100	1200	2.31	1.07	0.793 ± 0.009	2300	1200	1.80	0.948	0.797 ± 0.009
2100	1300	2.28	1.05	0.802 ± 0.009	2300	1300	1.78	0.937	0.806 ± 0.017
2100	1400	2.29	1.06	0.799 ± 0.009	2300	1400	1.77	0.933	0.810 ± 0.008
2100	1500	2.29	1.06	0.797 ± 0.008	2300	1500	1.77	0.932	0.811 ± 0.009
2100	1600	2.31	1.07	0.790 ± 0.008	2300	1600	1.77	0.933	0.809 ± 0.009
2100	1700	2.35	1.09	0.776 ± 0.009	2300	1700	1.78	0.939	0.803 ± 0.009
2100	1800	2.41	1.12	0.756 ± 0.009	2300	1800	1.79	0.946	0.798 ± 0.017
2100	1900	2.52	1.16	0.725 ± 0.008	2300	1900	1.82	0.958	0.787 ± 0.009
2100	2000	2.85	1.32	0.641 ± 0.009	2300	2000	1.87	0.988	0.763 ± 0.009
2200	100	159	76.4	0.021 ± 0.187	2300	2100	1.96	1.03	0.730 ± 0.010
2200	200	17.6	8.46	0.129 ± 0.076	2300	2200	2.19	1.15	0.654 ± 0.009
2200	300	6.82	3.28	0.289 ± 0.037					
2200	400	4.30	2.07	0.446 ± 0.021					
2200	500	3.35	1.61	0.557 ± 0.014					
2200	600	2.87	1.38	0.637 ± 0.011					
2200	700	2.60	1.25	0.696 ± 0.010					
2200	800	2.44	1.17	0.728 ± 0.009					
2200	900	2.35	1.13	0.760 ± 0.009					
2200	1000	2.27	1.09	0.780 ± 0.008					
2200	1100	2.23	1.07	0.788 ± 0.009					
2200	1200	2.20	1.06	0.796 ± 0.021					
2200	1300	2.17	1.04	0.808 ± 0.009					
2200	1400	2.19	1.06	0.798 ± 0.009					
2200	1500	2.18	1.05	0.804 ± 0.009					
2200	1600	2.18	1.05	0.803 ± 0.009					
2200	1700	2.20	1.06	0.795 ± 0.008					
2200	1800	2.23	1.07	0.783 ± 0.009					
2200	1900	2.29	1.10	0.765 ± 0.009					
2200	2000	2.40	1.16	0.728 ± 0.009					
2200	2100	2.69	1.29	0.651 ± 0.017					

Table A.20. The 95% CL observed (Obs.) and expected (Exp.) exclusion limits (in fb) on the W_R production cross section times branching fraction for $W_R \to (ee+\mu\mu)jj$ as a function of W_R and N_ℓ mass (in GeV) for $2400 \leq M_{W_R} \leq 2500$ GeV. The signal acceptance (Acc.) is also included for each (M_{W_R}, M_{N_ℓ}) entry.

M_{W_R}	M_{N_ℓ}	Obs.	Exp.	Acc.	M_{W_R}	M_{N_ℓ}	Obs.	Exp.	Acc.
2400	100	151	88.1	0.022 ± 0.207	2500	100	153	98.3	0.024 ± 0.217
2400	200	14.0	8.18	0.121 ± 0.088	2500	200	13.8	8.85	0.119 ± 0.095
2400	300	5.00	2.93	0.268 ± 0.044	2500	300	4.71	3.02	0.254 ± 0.049
2400	400	2.90	1.69	0.418 ± 0.032	2500	400	2.67	1.71	0.401 ± 0.032
2400	500	2.15	1.26	0.537 ± 0.016	2500	500	1.88	1.21	0.527 ± 0.018
2400	600	1.79	1.05	0.624 ± 0.012	2500	600	1.56	0.999	0.612 ± 0.013
2400	700	1.63	0.956	0.683 ± 0.010	2500	700	1.38	0.884	0.674 ± 0.011
2400	800	1.53	0.895	0.725 ± 0.009	2500	800	1.28	0.818	0.722 ± 0.009
2400	900	1.55	0.909	0.751 ± 0.010	2500	900	1.20	0.772	0.744 ± 0.009
2400	1000	1.50	0.880	0.773 ± 0.009	2500	1000	1.17	0.753	0.771 ± 0.009
2400	1100	1.44	0.843	0.789 ± 0.008	2500	1100	1.14	0.733	0.785 ± 0.009
2400	1200	1.40	0.820	0.803 ± 0.009	2500	1200	1.13	0.724	0.798 ± 0.009
2400	1300	1.39	0.815	0.809 ± 0.009	2500	1300	1.12	0.717	0.805 ± 0.008
2400	1400	1.39	0.815	0.807 ± 0.008	2500	1400	1.11	0.712	0.811 ± 0.008
2400	1500	1.39	0.813	0.810 ± 0.008	2500	1500	1.10	0.707	0.816 ± 0.009
2400	1600	1.38	0.809	0.814 ± 0.009	2500	1600	1.09	0.700	0.824 ± 0.009
2400	1700	1.38	0.809	0.813 ± 0.012	2500	1700	1.11	0.710	0.812 ± 0.011
2400	1800	1.40	0.818	0.804 ± 0.012	2500	1800	1.11	0.713	0.809 ± 0.010
2400	1900	1.41	0.825	0.798 ± 0.009	2500	1900	1.11	0.710	0.812 ± 0.012
2400	2000	1.43	0.834	0.789 ± 0.008	2500	2000	1.12	0.718	0.803 ± 0.008
2400	2100	1.46	0.852	0.772 ± 0.008	2500	2100	1.13	0.726	0.794 ± 0.008
2400	2200	1.53	0.894	0.736 ± 0.009	2500	2200	1.17	0.750	0.769 ± 0.010
2400	2300	1.70	0.995	0.660 ± 0.011	2500	2300	1.22	0.781	0.737 ± 0.009
					2500	2400	1.36	0.869	0.662 ± 0.009

Table A.21. The 95% CL observed (Obs.) and expected (Exp.) exclusion limits (in fb) on the W_R production cross section times branching fraction for $W_R \to (ee+\mu\mu)jj$ as a function of W_R and N_ℓ mass (in GeV) for $2600 \leq M_{W_R} \leq 2700$ GeV. The signal acceptance (Acc.) is also included for each (M_{W_R}, M_{N_ℓ}) entry.

M_{W_R}	M_{N_ℓ}	Obs.	Exp.	Acc.	M_{W_R}	M_{N_ℓ}	Obs.	Exp.	Acc.
2600	100	136	98.1	0.021 ± 0.225	2700	100	134	94.6	0.023 ± 0.236
2600	200	13.3	9.58	0.115 ± 0.101	2700	200	14.1	9.94	0.120 ± 0.109
2600	300	4.42	3.19	0.246 ± 0.053	2700	300	4.70	3.31	0.247 ± 0.058
2600	400	2.38	1.72	0.392 ± 0.031	2700	400	2.48	1.75	0.381 ± 0.034
2600	500	1.66	1.20	0.509 ± 0.019	2700	500	1.70	1.20	0.500 ± 0.021
2600	600	1.34	0.966	0.605 ± 0.014	2700	600	1.34	0.940	0.601 ± 0.015
2600	700	1.17	0.842	0.672 ± 0.011	2700	700	1.18	0.831	0.663 ± 0.012
2600	800	1.07	0.774	0.715 ± 0.010	2700	800	1.10	0.772	0.710 ± 0.010
2600	900	1.01	0.731	0.738 ± 0.010	2700	900	1.02	0.715	0.740 ± 0.009
2600	1000	0.965	0.698	0.765 ± 0.009	2700	1000	0.943	0.664	0.768 ± 0.009
2600	1100	0.931	0.672	0.778 ± 0.009	2700	1100	0.914	0.643	0.780 ± 0.011
2600	1200	0.937	0.677	0.799 ± 0.009	2700	1200	0.861	0.606	0.794 ± 0.009
2600	1300	0.909	0.657	0.803 ± 0.009	2700	1300	0.867	0.611	0.806 ± 0.009
2600	1400	0.903	0.652	0.808 ± 0.009	2700	1400	0.882	0.621	0.810 ± 0.013
2600	1500	0.899	0.649	0.812 ± 0.009	2700	1500	0.874	0.615	0.817 ± 0.010
2600	1600	0.888	0.641	0.821 ± 0.010	2700	1600	0.868	0.611	0.822 ± 0.010
2600	1700	0.887	0.641	0.822 ± 0.012	2700	1700	0.870	0.613	0.819 ± 0.019
2600	1800	0.891	0.644	0.818 ± 0.018	2700	1800	0.866	0.610	0.823 ± 0.011
2600	1900	0.894	0.646	0.816 ± 0.009	2700	1900	0.869	0.612	0.820 ± 0.009
2600	2000	0.899	0.650	0.810 ± 0.009	2700	2000	0.870	0.612	0.820 ± 0.008
2600	2100	0.905	0.654	0.805 ± 0.009	2700	2100	0.873	0.614	0.816 ± 0.011
2600	2200	0.917	0.663	0.794 ± 0.011	2700	2200	0.888	0.625	0.802 ± 0.009
2600	2300	0.940	0.679	0.775 ± 0.009	2700	2300	0.899	0.633	0.792 ± 0.013
2600	2400	0.988	0.714	0.737 ± 0.009	2700	2400	0.921	0.648	0.774 ± 0.008
2600	2500	1.10	0.792	0.664 ± 0.010	2700	2500	0.965	0.679	0.738 ± 0.009
					2700	2600	1.05	0.743	0.674 ± 0.010

Table A.22. The 95% CL observed (Obs.) and expected (Exp.) exclusion limits (in fb) on the W_R production cross section times branching fraction for $W_R \to (ee+\mu\mu)jj$ as a function of W_R and N_ℓ mass (in GeV) for $2800 \leq M_{W_R} \leq 2900$ GeV. The signal acceptance (Acc.) is also included for each (M_{W_R}, M_{N_ℓ}) entry.

M_{W_R}	M_{N_ℓ}	Obs.	Exp.	Acc.	M_{W_R}	M_{N_ℓ}	Obs.	Exp.	Acc.
2800	100	143	101	0.025 ± 0.248	2900	100	140	100	0.027 ± 0.255
2800	200	16.5	11.6	0.123 ± 0.117	2900	200	18.1	12.9	0.123 ± 0.126
2800	300	5.46	3.86	0.245 ± 0.063	2900	300	5.90	4.21	0.236 ± 0.070
2800	400	2.81	1.99	0.372 ± 0.037	2900	400	2.97	2.12	0.377 ± 0.042
2800	500	1.86	1.32	0.494 ± 0.023	2900	500	1.91	1.37	0.485 ± 0.030
2800	600	1.45	1.02	0.589 ± 0.016	2900	600	1.46	1.04	0.582 ± 0.017
2800	700	1.22	0.861	0.655 ± 0.013	2900	700	1.22	0.873	0.647 ± 0.014
2800	800	1.12	0.793	0.699 ± 0.010	2900	800	1.08	0.769	0.692 ± 0.011
2800	900	1.01	0.713	0.731 ± 0.010	2900	900	0.986	0.704	0.729 ± 0.010
2800	1000	0.955	0.675	0.758 ± 0.011	2900	1000	0.930	0.664	0.751 ± 0.009
2800	1100	0.913	0.646	0.773 ± 0.011	2900	1100	0.886	0.632	0.772 ± 0.015
2800	1200	0.882	0.624	0.794 ± 0.010	2900	1200	0.853	0.609	0.790 ± 0.011
2800	1300	0.857	0.606	0.804 ± 0.009	2900	1300	0.827	0.590	0.805 ± 0.009
2800	1400	0.844	0.597	0.811 ± 0.009	2900	1400	0.810	0.578	0.811 ± 0.009
2800	1500	0.836	0.591	0.819 ± 0.009	2900	1500	0.800	0.571	0.814 ± 0.009
2800	1600	0.830	0.587	0.824 ± 0.009	2900	1600	0.791	0.565	0.823 ± 0.009
2800	1700	0.826	0.584	0.827 ± 0.009	2900	1700	0.791	0.565	0.823 ± 0.010
2800	1800	0.827	0.585	0.826 ± 0.009	2900	1800	0.783	0.559	0.831 ± 0.017
2800	1900	0.829	0.586	0.824 ± 0.009	2900	1900	0.785	0.561	0.829 ± 0.010
2800	2000	0.830	0.587	0.823 ± 0.009	2900	2000	0.782	0.559	0.831 ± 0.021
2800	2100	0.832	0.588	0.821 ± 0.009	2900	2100	0.797	0.569	0.816 ± 0.009
2800	2200	0.843	0.596	0.810 ± 0.009	2900	2200	0.792	0.565	0.821 ± 0.010
2800	2300	0.850	0.601	0.803 ± 0.009	2900	2300	0.798	0.570	0.815 ± 0.010
2800	2400	0.857	0.606	0.796 ± 0.009	2900	2400	0.805	0.575	0.808 ± 0.011
2800	2500	0.879	0.622	0.776 ± 0.008	2900	2500	0.814	0.581	0.798 ± 0.009
2800	2600	0.921	0.651	0.741 ± 0.011	2900	2600	0.838	0.598	0.776 ± 0.010
2800	2700	1.01	0.711	0.678 ± 0.010	2900	2700	0.876	0.625	0.742 ± 0.009
					2900	2800	0.947	0.676	0.686 ± 0.013

Table A.23. The 95% CL observed (Obs.) and expected (Exp.) exclusion limits (in fb) on the W_R production cross section times branching fraction for $W_R \to (ee+\mu\mu)jj$ as a function of W_R and N_ℓ mass (in GeV) for $3000 \le M_{W_R} \le 3100$ GeV. The signal acceptance (Acc.) is also included for each (M_{W_R}, M_{N_ℓ}) entry.

M_{W_R}	M_{N_ℓ}	Obs.	Exp.	Acc.	M_{W_R}	M_{N_ℓ}	Obs.	Exp.	Acc.
3000	100	137	99.7	0.027 ± 0.264	3100	100	141	103	0.032 ± 0.269
3000	200	20.3	14.7	0.124 ± 0.135	3100	200	23.0	16.7	0.137 ± 0.142
3000	300	6.40	4.66	0.245 ± 0.078	3100	300	7.33	5.32	0.250 ± 0.086
3000	400	3.21	2.33	0.369 ± 0.048	3100	400	3.53	2.56	0.373 ± 0.054
3000	500	2.04	1.49	0.482 ± 0.030	3100	500	2.24	1.63	0.475 ± 0.034
3000	600	1.52	1.10	0.573 ± 0.019	3100	600	1.63	1.18	0.565 ± 0.022
3000	700	1.25	0.908	0.637 ± 0.014	3100	700	1.32	0.959	0.634 ± 0.018
3000	800	1.09	0.796	0.685 ± 0.012	3100	800	1.14	0.827	0.685 ± 0.012
3000	900	0.993	0.722	0.721 ± 0.011	3100	900	1.02	0.744	0.724 ± 0.012
3000	1000	0.928	0.675	0.757 ± 0.010	3100	1000	0.952	0.691	0.745 ± 0.010
3000	1100	0.879	0.640	0.773 ± 0.011	3100	1100	0.893	0.648	0.768 ± 0.011
3000	1200	0.845	0.614	0.785 ± 0.012	3100	1200	0.857	0.622	0.787 ± 0.016
3000	1300	0.818	0.595	0.795 ± 0.012	3100	1300	0.827	0.600	0.798 ± 0.013
3000	1400	0.797	0.579	0.809 ± 0.011	3100	1400	0.799	0.580	0.809 ± 0.020
3000	1500	0.779	0.567	0.820 ± 0.009	3100	1500	0.779	0.565	0.812 ± 0.010
3000	1600	0.775	0.564	0.823 ± 0.010	3100	1600	0.772	0.560	0.823 ± 0.017
3000	1700	0.769	0.560	0.829 ± 0.010	3100	1700	0.765	0.556	0.829 ± 0.010
3000	1800	0.771	0.561	0.827 ± 0.011	3100	1800	0.765	0.555	0.829 ± 0.017
3000	1900	0.771	0.561	0.827 ± 0.013	3100	1900	0.765	0.555	0.828 ± 0.011
3000	2000	0.766	0.557	0.833 ± 0.016	3100	2000	0.760	0.552	0.834 ± 0.011
3000	2100	0.767	0.558	0.831 ± 0.009	3100	2100	0.765	0.555	0.828 ± 0.024
3000	2200	0.774	0.563	0.823 ± 0.022	3100	2200	0.762	0.554	0.831 ± 0.012
3000	2300	0.773	0.563	0.824 ± 0.014	3100	2300	0.765	0.556	0.827 ± 0.011
3000	2400	0.779	0.567	0.818 ± 0.010	3100	2400	0.770	0.559	0.822 ± 0.009
3000	2500	0.788	0.573	0.808 ± 0.011	3100	2500	0.777	0.564	0.814 ± 0.009
3000	2600	0.798	0.581	0.798 ± 0.009	3100	2600	0.782	0.567	0.810 ± 0.009
3000	2700	0.814	0.592	0.782 ± 0.009	3100	2700	0.792	0.575	0.799 ± 0.010
3000	2800	0.853	0.621	0.746 ± 0.009	3100	2800	0.812	0.590	0.779 ± 0.009
3000	2900	0.923	0.672	0.689 ± 0.013	3100	2900	0.845	0.613	0.749 ± 0.009
					3100	3000	0.915	0.664	0.692 ± 0.013

Table A.24. The 95% CL observed (Obs.) and expected (Exp.) exclusion limits (in fb) on the W_R production cross section times branching fraction for $W_R \to (ee + \mu\mu)jj$ as a function of W_R and N_ℓ mass (in GeV) for $M_{W_R} = 3200\,GeV$. The signal acceptance (Acc.) is also included for each (M_{W_R}, M_{N_ℓ}) entry.

M_{W_R}	M_{N_ℓ}	Obs.	Exp.	Acc.
3200	100	144	104	0.035 ± 0.274
3200	200	25.9	18.7	0.139 ± 0.147
3200	300	8.16	5.90	0.265 ± 0.091
3200	400	3.98	2.88	0.376 ± 0.059
3200	500	2.46	1.78	0.471 ± 0.041
3200	600	1.77	1.28	0.560 ± 0.025
3200	700	1.41	1.02	0.626 ± 0.019
3200	800	1.20	0.871	0.679 ± 0.014
3200	900	1.07	0.772	0.712 ± 0.016
3200	1000	0.983	0.711	0.741 ± 0.012
3200	1100	0.920	0.665	0.768 ± 0.016
3200	1200	0.870	0.629	0.781 ± 0.010
3200	1300	0.838	0.606	0.795 ± 0.016
3200	1400	0.809	0.585	0.804 ± 0.013
3200	1500	0.785	0.568	0.813 ± 0.011
3200	1600	0.769	0.556	0.823 ± 0.010
3200	1700	0.767	0.555	0.824 ± 0.016
3200	1800	0.760	0.550	0.831 ± 0.013
3200	1900	0.758	0.548	0.832 ± 0.016
3200	2000	0.756	0.547	0.834 ± 0.020
3200	2100	0.762	0.551	0.828 ± 0.010
3200	2200	0.756	0.547	0.835 ± 0.013
3200	2300	0.762	0.551	0.829 ± 0.011
3200	2400	0.765	0.553	0.825 ± 0.009
3200	2500	0.770	0.557	0.819 ± 0.010
3200	2600	0.775	0.560	0.814 ± 0.010
3200	2700	0.778	0.563	0.811 ± 0.013
3200	2800	0.791	0.572	0.797 ± 0.012
3200	2900	0.813	0.588	0.776 ± 0.013
3200	3000	0.837	0.605	0.753 ± 0.010
3200	3100	0.909	0.658	0.693 ± 0.024
